# High-Temperature-Resistant Mid-Infrared Capability for Extreme Energy Systems

**DOI:** 10.1007/s40820-026-02290-w

**Published:** 2026-07-09

**Authors:** Jingru Huang, Jiamu Feng, Mincan Yang, Keshuai Liu, Bin Hu, Weilin Xu, Jun Wan

**Affiliations:** 1 School of Chemistry and Chemical Engineering, Wuhan Textile University, Wuhan, 430200 People’s Republic of China; 2 State Key Laboratory of New Textile Materials and Advanced Processing, Wuhan, 430200 People’s Republic of China; 3 Wuhan National Laboratory for Optoelectronics, School of Optical and Electronic Information, Huazhong University of Science and Technology, Wuhan, 430074 People’s Republic of China

**Keywords:** Mid-infrared, High-temperature-resistant materials, Structure engineering, Solid-state chemistry, Thermal photonics

## Abstract

Extreme thermal environments encountered in hypersonic flight, space infrastructure, high-temperature energy conversion, and harsh industrial processes place mid-infrared radiation at the center of heat exchange, sensing, and energy management. As operating temperatures and environmental aggressiveness increase, conventional room temperature optical design becomes an unreliable predictor of in-service behavior, while the stability of radiative properties under coupled thermal, chemical, and mechanical loads emerges as a critical bottleneck. Despite extensive progress in individual material systems, a unified understanding that links material structure, degradation pathways, and durable mid-infrared functionality remains lacking. This review provides a structure-informed framework for high-temperature-resistant mid-infrared materials, resolving the field into three functional classes of high reflectance, high absorptance or emittance, and high transmittance, and interpreting their performance through four governing structural determinants spanning electronic and defect structure, crystallography and phase stability, microstructure and mesostructure, and high-temperature surface and interface evolution. By consolidating material classes, spectral bands, functional metrics, and temperature limits into a coherent dataset, the review enables like-for-like comparison and the extraction of transferable design descriptors. This perspective couples radiative function with thermal survivability, offering design rules and a roadmap for developing predictable, field-qualified mid-infrared technologies capable of operating at the limits of temperature and energy flux.

## Highlights

- Structure-informed rules linking carriers, phonons, microstructure, and surfaces to mid-infrared (MIR) performance at high temperature.
- Unified taxonomy of high-temperature-resistant MIR reflectors, emitters, and transmitters with a consolidated dataset.
- Design principles that stabilize optical constants under heat and reactive atmospheres for hypersonic windows and radiative skins.

## Graphical abstract

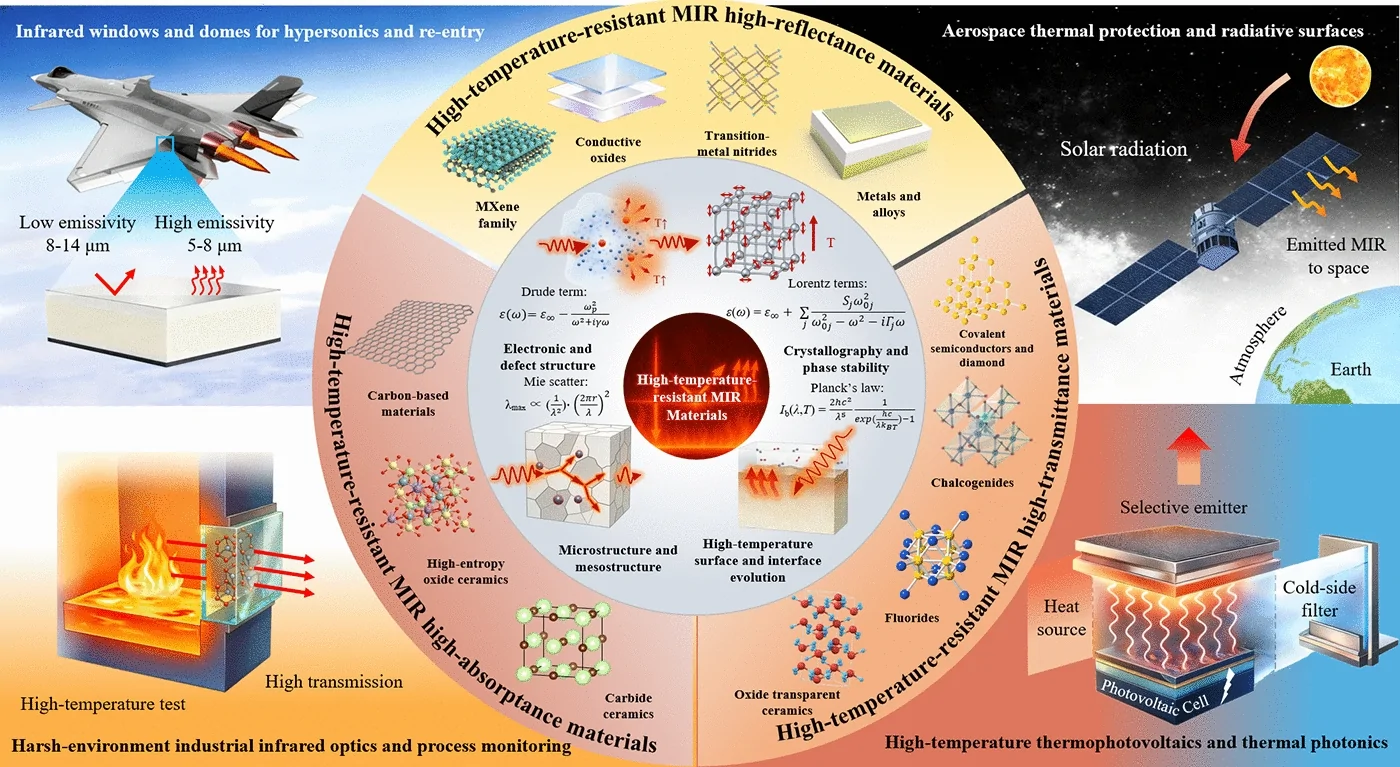

## Introduction

Extreme thermal environments define the operational boundaries of many of today’s most advanced energy and technological systems, including hypersonic flight [1] and atmospheric re-entry [2], space-based infrastructure, high-temperature energy conversion [3], and harsh-environment industrial processes [4]. In these regimes, heat transport and thermal radiation are not secondary considerations but primary determinants of system efficiency, functionality, and lifetime. As temperatures rise beyond the limits of conventional materials, the mid-infrared (MIR) spectral range becomes the dominant channel for energy exchange, governing radiative heat loss, thermal camouflage, sensing, and energy harvesting [5, 6]. The ability to manipulate MIR radiation under sustained thermal, chemical, and mechanical stress therefore underpins the performance of a broad class of extreme systems, positioning high-temperature MIR materials at the intersection of energy management, thermal protection, and functional optics [7].

Unlike optical materials designed for ambient conditions, MIR materials operating at elevated temperature are subject to a fundamental coupling between radiative function and thermal degradation. Optical performance is no longer determined solely by intrinsic electronic or vibrational structure at room temperature, but by the evolution of complex permittivity as carrier populations, phonon lifetimes, defect concentrations, and surface states continuously adapt to temperature and atmosphere [8]. Through Kirchhoff’s law, emissivity is intrinsically linked to absorptance, such that any temperature-induced change in free-carrier scattering, phonon damping [9], or defect-assisted absorption simultaneously reshapes thermal emission. Drude–Lorentz responses shift as carrier density and mobility evolve, while Reststrahlen bands in polar crystals broaden and migrate with increasing anharmonicity [10]. These mechanisms define three distinct radiative control modes: suppression of radiative loss dominated by reflection [11], heat evacuation dominated by emission [12], and infrared access dominated by transmission [13], while simultaneously imposing inherent trade-offs that become more severe at elevated temperatures. Processes such as oxidation, volatilization, non-stoichiometry drift, interdiffusion, grain coarsening, and surface roughening progressively distort optical constants and inflate apparent emissivity, rendering room temperature optimization a poor predictor of in-service behavior.

From a design perspective, the challenge is not merely to identify materials that exhibit desirable MIR spectra, but to sustain those spectra under extreme operating conditions [14]. High reflectance with low emittance requires maintaining a plasma frequency well above the MIR band together with minimal damping, which becomes increasingly sensitive to carrier scattering, impurities, and grain boundaries within the electromagnetic skin depth at high temperature [15]. High or selective emittance relies on controlled free-carrier loss or strong phonon–polariton resonances, yet spectral selectivity degrades as oscillator strength and linewidth evolve with temperature, defect formation, and chemical disorder [16]. High transmittance imposes an additional and often competing constraint set, demanding suppression of free-carrier and multi-phonon absorption [17], high densification to minimize scattering, and long-term resistance to color center formation and surface damage [18]. Although numerous material strategies, including doping, phase and texture control, densification, surface passivation, and device-level patterning, have been demonstrated to address specific aspects of these requirements, these approaches are generally optimized within narrowly defined temperature and atmospheric regimes and are reported using non-uniform performance metrics. Consequently, previous reviews have mainly focused on thermal radiation devices, radiative cooling, thermophotovoltaics, infrared photonics, or specific material families, while the coupled relationship between MIR function, structural evolution, and high-temperature degradation across material classes remains insufficiently clarified. As a result, the current understanding remains fragmented, and predictive relationships connecting structure, degradation pathways, and MIR performance across material classes and operating regimes are still limited.

**Fig. 1:**
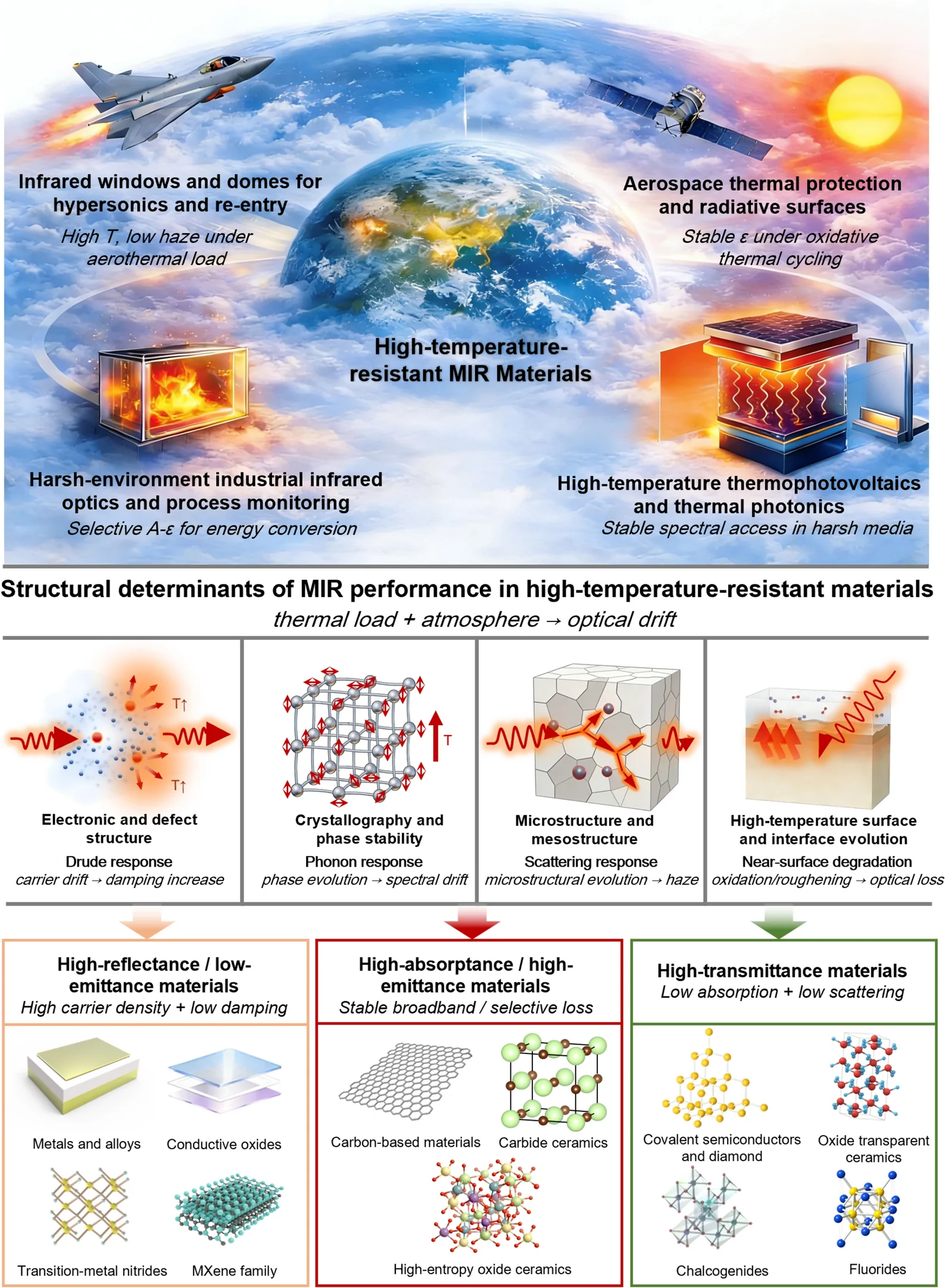
Schematic overview of high-temperature-resistant MIR materials and applications

This review addresses these challenges by establishing a structure-informed framework for high-temperature MIR materials that connects radiative function directly to thermal survivability (Fig. 1). Rather than classifying materials purely by composition, the field is organized into three functional categories, namely high reflectance, high absorptance or high emittance, and high transmittance, and is systematically interpreted through four governing structural determinants. These include electronic and defect structures characterized by carrier concentration and non-stoichiometry; crystallography and phase stability defined by symmetry, anisotropy, and phonon spectra; microstructural and mesostructural features such as densification, grain size and boundaries, porosity, and texture; and the evolution of surfaces and interfaces under high-temperature conditions, including oxide scale formation, roughness development, and electromagnetic skin effects. By mapping metals and alloys, conductive oxides, transition metal nitrides, carbon-based materials, oxide and carbide ceramics, covalent semiconductors, and infrared-transparent compounds within this unified framework, the review enables like-for-like comparison across material families and operating conditions. Application-oriented analyses further link these structure–property relationships to hypersonic and re-entry apertures, aerospace thermal protection and radiative surfaces, high-temperature thermophotovoltaics and thermal photonics, and harsh-environment industrial optics. By coupling MIR spectral control with structural stability under extreme conditions, this perspective aims to deliver transferable design principles, guide materials selection and processing, and accelerate the translation of laboratory concepts into field-qualified technologies operating at the limits of temperature and energy flux.

## High-Temperature-Resistant Mid-Infrared Materials

### Definitions and Classification Frameworks of High-Temperature-Resistant Mid-Infrared Materials

**Fig. 2:**
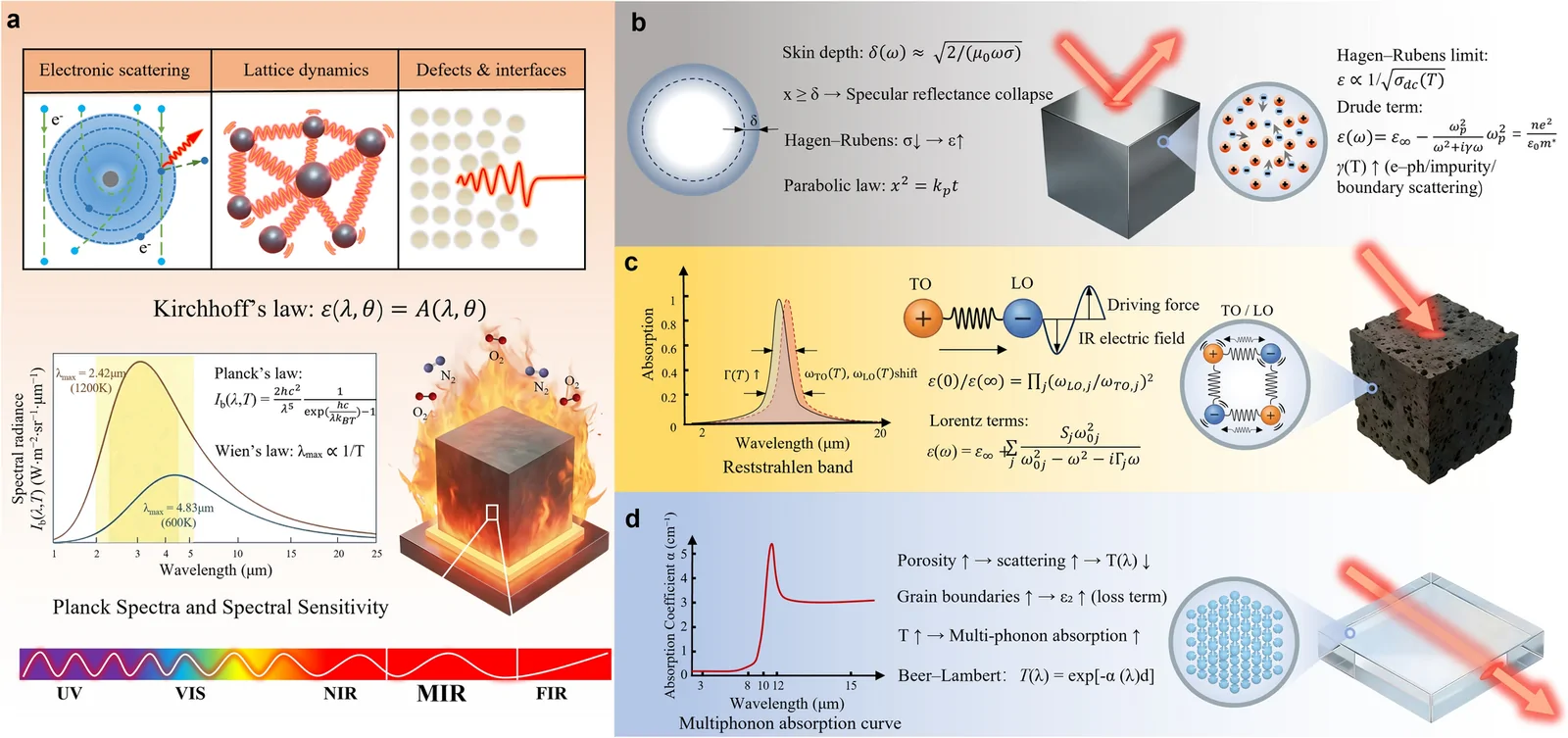
Temperature-dependent mid-infrared performance and mechanistic stability of optical components. **a** Volution of spectral reflectance for mirrors governed by plasma frequency, damping rate, and surface oxidation. **b** Drift of phonon resonances and emission linewidths in selective emitters under thermal and structural evolution. **c** Broadband transmittance of window materials limited by free-carrier density, multi-phonon absorption, and scattering. **d** Correlation between surface roughness, scale thickness, and long-wave emissivity over the intended thermal dwell

In this review, mid-infrared refers mainly to the 2.5–25 μm spectral region, with emphasis on the 3–5 and 8–12 μm atmospheric windows, while high-temperature-resistant describes the retention of targeted MIR reflectance, emittance, or transmittance within material-specific temperature-atmosphere windows. Mid-infrared optical behavior under such conditions is governed by two coupled constraints **(**Fig. 2**)**: radiative energy balance, which defines the boundary conditions of spectral response, and temperature-dependent dielectric evolution under thermal, chemical, and mechanical loading. For any material surface, radiative exchange satisfies $R\left(\lambda ,\theta \right)+A\left(\lambda ,\theta \right)+T\left(\lambda ,\theta \right)=1$. At thermal equilibrium, Kirchhoff’s law gives $\varepsilon (\lambda ,\theta )=A(\lambda ,\theta )$ [19]. In practical applications, however, performance is usually evaluated by spectrally integrated hemispherical quantities rather than monochromatic values, because net emission or absorption depends on wavelength, angle, and temperature-weighted radiance [20]. This distinction is especially important for rough, textured, or curved surfaces, where angular redistribution can be substantial. Therefore, angle-resolved relations are used here as the physical basis, while hemispherical averages are emphasized as practical comparison metrics [21].

At the microscopic level, mid-infrared optical response is determined by the complex dielectric function$\varepsilon \left(\omega \right)={\varepsilon}_{1}\left(\omega \right)+i{\varepsilon}_{2}(\omega )$, or equivalently by the complex refractive index. In metals, transition metal nitrides, and some carbides, free carriers dominate the response and are commonly described by the Drude model, $\varepsilon \left(\omega \right)={\varepsilon}_{\infty }-\frac{{\omega}_{p}^{2}}{{\omega }^{2}+i\gamma \omega }$ [22], where the plasma frequency and damping rate govern mid-infrared reflectance and emissivity [23]. High reflectance or low emissivity therefore requires a plasma frequency above the target spectral band and sufficiently low damping. The Drude description is most appropriate for systems with delocalized carriers and quasi-free-electron transport; in strongly correlated, highly disordered, or defect-rich materials, deviations may arise from carrier localization, interband transitions, polaronic effects, or enhanced defect scattering. Its use should therefore be regarded as a limiting framework rather than a universal description.

In polar crystals and most oxide ceramics, free-carrier contributions are weaker, and lattice vibrations become the dominant mechanism. Their dielectric response is better described by Lorentz oscillators, $\varepsilon \left( \omega \right) = \mathop \sum \limits_{j} \frac{{S_{j} \omega_{0j}^{2} }}{{\omega_{0j}^{2} - \omega^{2} - i{\Gamma}_{j} \omega }}$, where phonon–polariton coupling produces Reststrahlen bands between transverse and longitudinal optical phonon frequencies [24], The Lyddane–Sachs–Teller relation, $\varepsilon \left( 0 \right)/\varepsilon \left( \infty \right) = \mathop \prod \limits_{j} \left( {\omega_{LO,j} /\omega_{TO,j} } \right)^{2}$, links dielectric constants to phonon spectra [25]. For bulk media, internal transmittance follows the Beer–Lambert relation, while interface reflection is governed by Fresnel relations [26, 27]. In this review, these expressions are introduced not for detailed derivation, but to identify the main intrinsic design variables: carrier density, damping rate, phonon resonance position and linewidth, absorption coefficient, refractive index, and thickness.

Temperature modifies these variables through two distinct but often coupled routes. Intrinsic thermal effects originate from lattice and carrier dynamics, including increased phonon populations, enhanced electron–phonon or phonon–phonon scattering, and anharmonic broadening of phonon resonances; these effects usually cause gradual and partly reversible changes in dielectric response, especially in vacuum or inert atmospheres. As temperature increases [28],, carrier mobility generally decreases and damping increases, which can elevate long-wavelength emissivity even when carrier concentration is nearly unchanged [29]. In polar materials, anharmonic interactions broaden resonance linewidths and shift phonon frequencies, causing Reststrahlen bands to drift with temperature [30, 31]. By contrast, environment-induced effects arise from oxidation, volatilization, hydration, interdiffusion, or non-stoichiometry drift; these processes alter carrier concentration, defect chemistry, and surface/interface structure more abruptly and often irreversibly, becoming dominant in oxidizing or reactive atmospheres. Distinguishing these two contributions is essential because the same material may exhibit stable intrinsic thermal broadening in inert conditions but rapid optical degradation once surface chemistry is activated.

Near-surface regions are particularly important under high-temperature operation. For good conductors, mid-infrared electromagnetic fields penetrate only within the skin depth $\delta \left( \omega \right) \approx \sqrt {2/\left( {\mu_{0} \omega \sigma } \right)}$ [32], As a practical guideline, once oxide scale thickness or characteristic roughness approaches the skin depth, typically from tens to hundreds of nanometers in the MIR for highly conductive materials, specular reflectance may decrease markedly and hemispherical emissivity may increase, even if bulk conductivity is largely retained [33, 34]. Pore formation, microcracking, or poorly adherent surface scales further amplify scattering and accelerate optical degradation [35, 36].

Microstructure determines whether intrinsic optical properties can be preserved at the engineering scale [37, 38]. Pores, grain boundaries, and rough interfaces introduce additional scattering and absorption, thereby degrading transmittance or reflectance [39, 40]. Their influence depends strongly on their characteristic size relative to the operating wavelength. For this reason, microstructural control should be considered not as a secondary processing issue [41], but as a direct part of mid-infrared performance design under thermal exposure [42].

Intrinsic absorption sets the physical limits of achievable performance. For highly conductive Drude-like materials in the long-wavelength limit, the Hagen–Rubens relation provides a useful approximation linking low-frequency reflectance or emissivity to electrical conductivity [43]. This approximation becomes unreliable when conductivity is reduced, carrier localization or disorder is strong, or interband and defect-assisted absorption contribute appreciably. In polar dielectrics, long-wavelength cutoffs are instead imposed by multi-phonon absorption [44], which cannot be eliminated but can only be shifted through phase selection and bonding characteristics [45]. Structural photonic strategies remain valuable, but only when the underlying material preserves chemical and mechanical integrity at temperature [46].

Within this mechanistic framework, high reflectance, high absorptance, and high transmittance are not isolated categories, but temperature- and atmosphere-dependent operating regimes governed by the same parameters: carrier dynamics, phonon response, defect stability, microstructure, and near-surface evolution. Meaningful comparison across materials therefore requires consistent reporting of spectral range, angular condition, measurement geometry, atmosphere, exposure time, and optical property drift, so that intrinsic absorption can be distinguished from scattering and interface-induced losses.

## High-Temperature-Resistant Mid-Infrared High-Reflectance (Low-Emittance) Materials

**Fig. 3:**
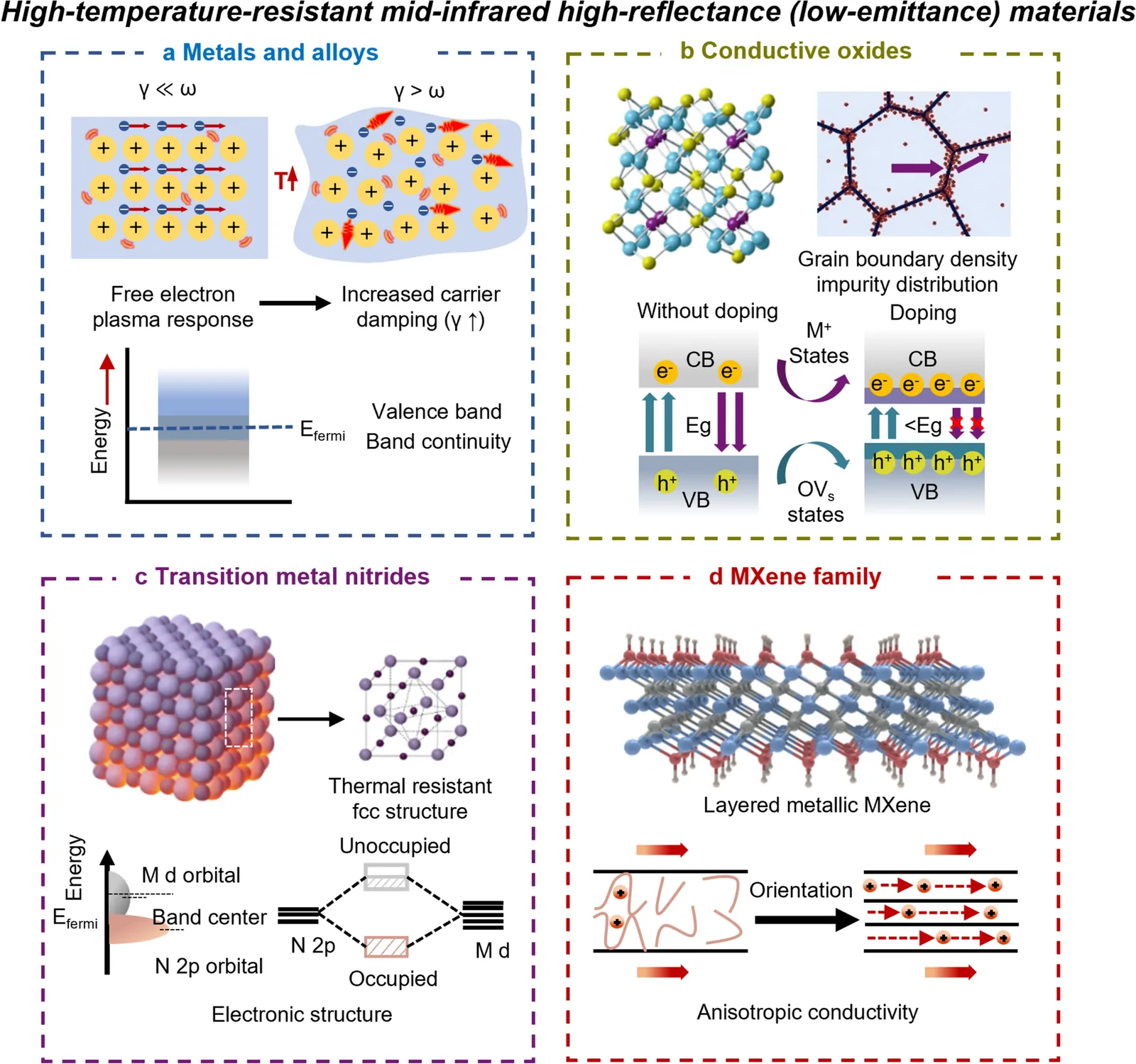
Classification and physical mechanisms of four intrinsic high-temperature-resistant mid-infrared high-reflectance (low-emittance) material families. **a** Metals and alloys: free-carrier plasma response, high carrier density, and temperature-enhanced damping. **b** Conductive oxides: plasma-edge tuning through doping, oxygen vacancies, and microstructural control. **c** Transition metal nitrides: refractory bonding coupled with metallic d-band plasmonic response. **d** MXene family: layered metallic architecture enabling anisotropic and orientation-dependent electron transport

High-temperature-resistant mid-infrared high-reflectance (low-emittance) materials achieve MIR performance when the screened plasma frequency lies above the target band and carrier damping remains sufficiently low. Rather than exhaustively covering all reported reflectors, this section focuses on four representative material families that define the main performance limits and degradation pathways under high-temperature conditions (Fig. 3). Metals and alloys provide the highest carrier density and minimal interband loss in the MIR, setting the benchmark for reflectance in vacuum or inert environments, but their stability in air is limited by oxidation, volatile scales, and roughening-induced diffuse loss [47, 48]. Conductive oxides, including transparent conducting oxides and metallic oxides, offer more atmosphere-tolerant reflectance through tunable plasma edges, although vacancy- and defect-induced damping must be controlled. Transition metal nitrides combine partially filled *d*-bands with refractory bonding, yielding a balanced window of plasmonic response, hardness, and thermal stability, but oxidation and nitrogen-vacancy scattering can raise optical loss [49–51]. MXenes contribute layered, high carrier architectures with anisotropic conductivity, yet their usable temperature window is constrained by surface termination evolution and oxygen-sensitive degradation [52]. Across these families, selection should therefore consider not only initial hemispherical or directional reflectance in the intended band, but also atmosphere-dependent drift in damping, surface roughness, and interfacial chemistry.

### Metals and Alloys

Metals and alloys deliver high mid-infrared reflectance through dense delocalized electrons and band structures in which interband transitions mainly lie outside the MIR window; thus, their optical response is dominated by low-loss free carriers, while temperature mainly increases damping through electron–phonon and impurity scattering [37, 53]. Refractory bcc metals such as W, Ta, Nb, and Mo offer high melting points, strong bonding, and large carrier densities, but oxygen-bearing flows promote oxide scale growth, volatile oxide evaporation, and surface roughening, thereby increasing apparent emissivity [54]. Noble and noble-like metals such as Ir and Pt better resist oxidation and maintain smoother conductive surfaces during heating, although density and cost restrict broader use. Alloy design therefore focuses on suppressing oxygen ingress, stabilizing microstructure, and limiting optically lossy surface phases [55, 56]. Additions that form adherent, slow-growing scales, such as Al- or Si-rich oxides in suitable matrices, can reduce subscale voiding and help preserve near-metallic optical constants within the electromagnetic skin depth. Importantly, roughness-induced emissivity depends on the ratio between roughness scale and MIR wavelength: subwavelength roughness remains largely specular, whereas roughness approaching the 3–12 μm MIR range promotes a specular-to-diffuse transition and geometric light trapping. In inert or vacuum environments, polished refractory metals can sustain very low emittance far above 1000 °C [48, 57, 58], whereas in oxidizing atmospheres the decisive limitation becomes scale growth, volatility, and optical loss [59]. During oxidation, reflectance may decrease slowly when the oxide remains thin and semi-transparent, but degradation accelerates once the scale thickens toward or beyond the skin-depth range and becomes absorbing, densified, or compositionally altered. Thus, effective high-temperature metallic reflectors require both a stable free-carrier response and surface chemistry that suppresses roughness, absorbing scales, and cyclic-stress-induced scattering.

**Fig. 4:**
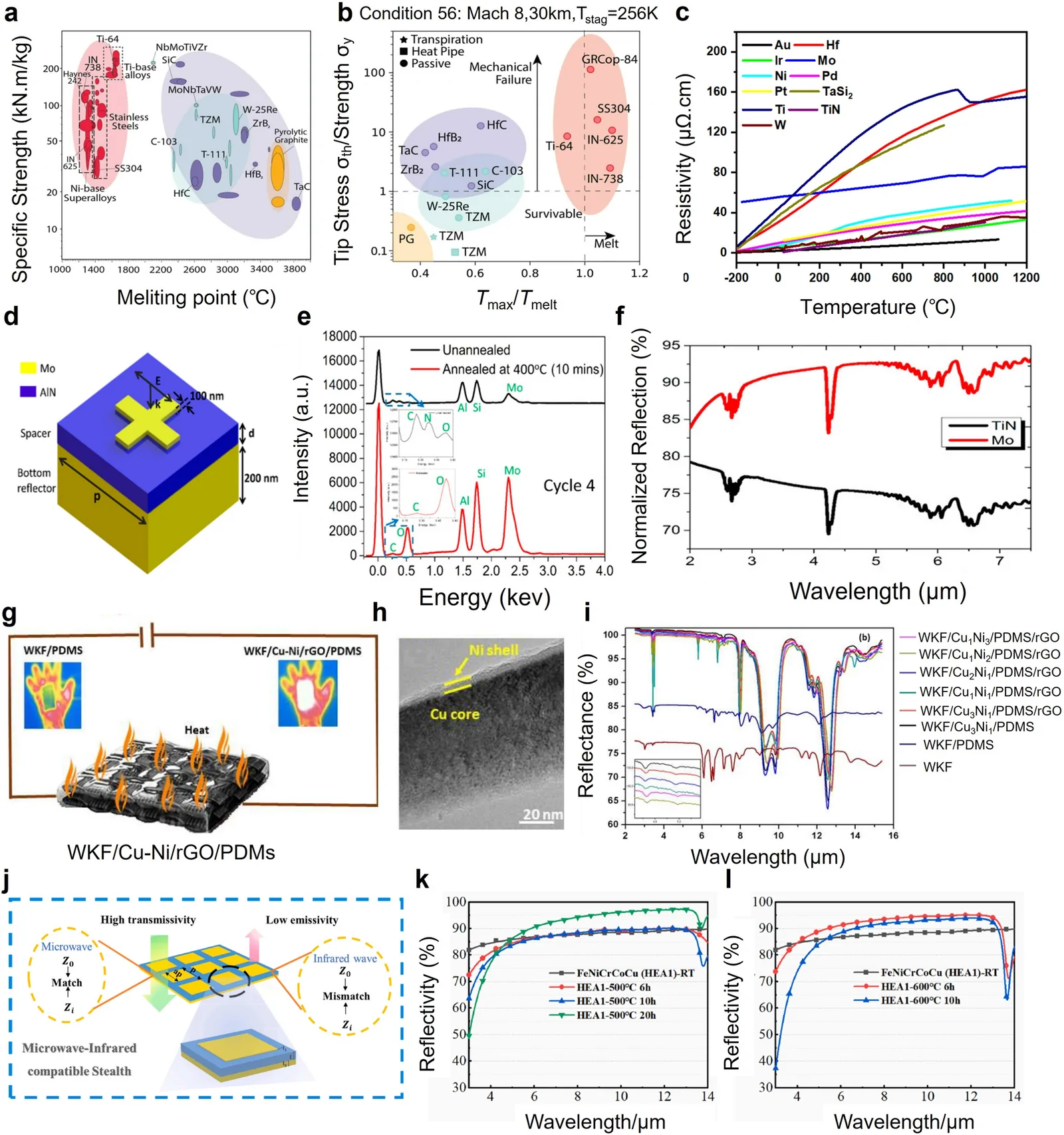
High-temperature-resistant MIR metals and alloys. **a** Ashby map of material trade-offs. **b** Stress-temperature Ashby plot. Reproduced with permission [48]. Copyright 2024, Springer Nature. **c** Temperature-dependent resistivity of metals. Reproduced with permission [57]. Copyright 2024, Springer Nature. **d** Mo-AlN-Mo structure. **e** SEM–EDS of AlN after cycling. **f** XPS and TEM of AlN films. Reproduced with permission [60]. Copyright 2017, American Chemical Society. **g** WKF-based reflector design. **h** TEM image of Cu-Ni nanowires on WKF. **i** FTIR reflectance spectra of composites. Reproduced with permission [61]. Copyright 2018, American Chemical Society. **j** Schematic of microwave-infrared compatible stealth coating. **k-l** Reflectance of FeNiCrCoCu after annealing. Reproduced with permission [37]. Copyright 2023, Elsevier

Recent studies define this design space from material screening to engineered reflector architectures. Ashby-type maps by Peters et al. correlate melting point, thermal conductivity, and emissivity to identify thermally resilient alloy families (Fig. 4a, b) [48], while resistivity-temperature relations reported by Jariwala et al. emphasize the role of phonon- and defect-mediated carrier scattering in high-temperature MIR damping (Fig. 4c) [57]. These system-level insights are reinforced by thin-film analyses that probe interfacial robustness and spectral response. For example, the Mo–AlN–Mo absorber investigated by Lee et al. (Fig. 4d) maintains compositional integrity and stable optical response under repeated thermal cycling up to 400 °C (after thermal equilibration), with only a minor decrease in resonance intensity (~ 10%) and negligible wavelength shift (~ 25 nm). Although FTIR-based reflectivity was used, the reported optical quantities are not explicitly defined (e.g., spectral or hemispherical). Meanwhile, comparative spectra of Mo and TiN films reveal a ∼20% MIR reflectance disparity at matched thickness (Fig. 4f) [60]. Together, these studies converge on a critical message: high-temperature MIR performance is governed not only by intrinsic electronic structure, but equally by how microstructure, interfaces, and defect populations evolve under thermal cycling.

Expanding beyond monolithic refractory metals, nanostructured conductors and multilayers show how geometry and interfaces can preserve reflectance under severe conditions. Vertically aligned Cu-Ni nanowires integrated into woven Kevlar fabrics form a hierarchical reflector architecture (Fig. 4g). TEM images confirm the vertical alignment and uniform morphology of Cu–Ni nanowires on WKF (Fig. 4h). FTIR spectra show MIR reflectance approaching 98.47% after rGO incorporation (Fig. 4i) [61]. FeNiCrCoCu-based metal–dielectric–metal structures provide a complementary route for microwave-infrared compatible stealth coatings (Fig. 4j). Correspondingly, the reflectance spectra of FeNiCrCoCu films annealed at 500 °C for 6–20 h (Fig. 4k) and 600 °C for 6–10 h (Fig. 4l) demonstrate a clear annealing-dependent evolution in infrared reflectance [37]. Overall, refractory metals and alloys remain most competitive above ~ 1000 °C in vacuum or inert atmospheres because of their high carrier density and stable plasma response; in oxidizing environments, oxide growth and roughening make them less suitable than conductive oxides for long-term low-emittance operation.

### Conductive Oxides (Transparent Conducting Oxides and Metallic Oxides)

Conductive oxides combine plasmonic-like free-carrier response with intrinsically oxidized lattices, making them more tolerant of oxygen-bearing environments than most nitrides, carbides, or base metals while retaining MIR spectral control [16]. Transparent conducting oxides, such as In_2_O_3_:Sn, ZnO:Al, ZnO:Ga, and SnO_2_:F, rely on heavy doping and controlled non-stoichiometry to raise carrier density without excessive compensation, whereas metallic oxides such as SrRuO_3_, LaNiO_3_, RuO_2_, IrO_2_, and ReO_3_ derive metal-like dielectric behavior from itinerant transition metal bands [62–64]. Phase-transition oxides such as VO_2_ provide an additional route for tunable MIR regulation, because their thermally or electrically driven metal–insulator transition can reversibly modulate free-carrier density, infrared reflectance, and emissivity [65, 66]. Their stability depends on whether carrier density can be maintained without increasing damping. In TCOs, dopant activation, oxygen vacancy concentration, grain boundaries, impurity segregation, porosity, and second phases jointly determine mobility and MIR loss [67–69]. Oxygen vacancies therefore play a dual role: moderate vacancy concentrations generate carriers and shift the plasma edge toward the MIR, whereas excessive or spatially inhomogeneous vacancies act as scattering centers, reduce mobility, increase damping, and convert carrier generation into optical loss [70]. At elevated temperature, oxygen chemical potential resets defect equilibria; oxidizing atmospheres may annihilate vacancies and depress carrier density, whereas overly reducing conditions can induce sub-oxide formation or cation volatility. Metallic oxides follow a related constraint, but their reflectance depends more strongly on the stability of the correlated metallic state, resistance to cation diffusion, and oxygen stoichiometry. SrRuO_3_ and RuO_2_ benefit from strong metal–oxygen bonding and reduced scale growth [71, 72], while LaNiO_3_ is more sensitive to A-site stoichiometry, interfacial diffusion, and annealing atmosphere [73–75] and annealing atmosphere decisive. Across both subfamilies, durable low-emittance performance requires a redox stable carrier reservoir, dense microstructure, and smooth surface/interface evolution.

**Fig. 5:**
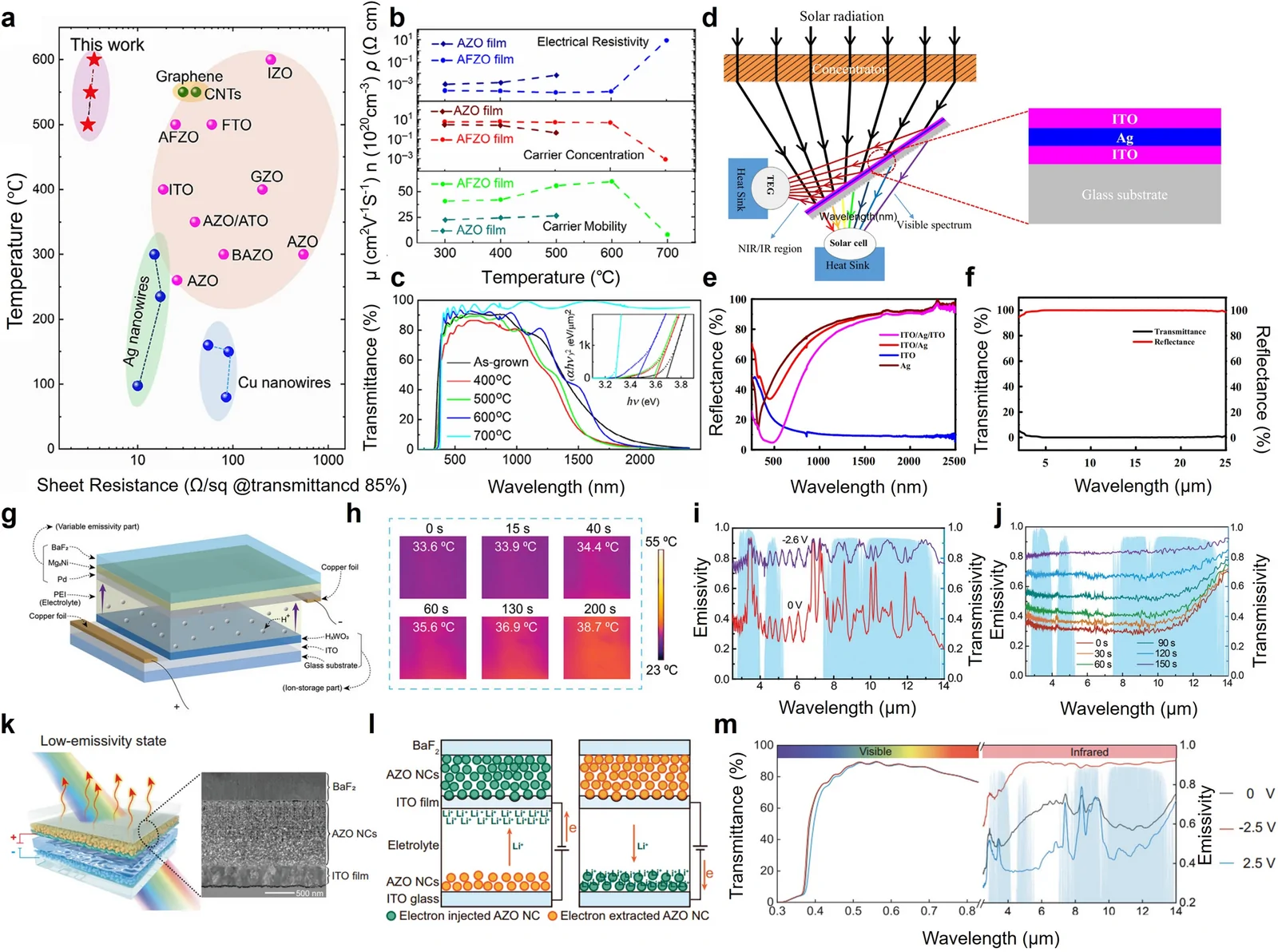
High-temperature-resistant MIR conductive oxides. **a** Conductivity vs. thermal stability of oxides. **b** Carrier transport vs. annealing temperature. **c** Transmission after annealing. Reproduced with permission [76]. Copyright 2024, John Wiley and Sons. **d** ITO/Ag/ITO structure. **e** Reflectance spectra of single and multilayer Ag/ITO on glass. **f** FTIR reflection and transmission spectra of multilayer system. Reproduced with permission [77]. Copyright 2017, Elsevier. **g** Dynamic emissivity device structure. **h** Infrared images of the flexible device at different times. **i** Emissivity curves of the flexible device. **j** Voltage-driven emissivity switching of the device. Reproduced with permission [78]. Copyright 2023, John Wiley and Sons. **k** Schematic of TDIE regulator structure. **l** Schematic of Li⁺ ion charge balancing in TDIE regulators. **m** Voltage-dependent optical response. Reproduced with permission [79]. Copyright 2023, Springer Nature

Building on this mechanistic understanding, several studies have quantified the translation of these principles into high-temperature mid-infrared performance. Liu et al. systematically evaluated a broad range of transparent conducting oxides, including ZnO, AZO, ITO, FTO, GZO, IZO, BAZO, ATO/AZO, and AFZO, by correlating sheet resistance at approximately 85% visible transmittance with maximum operational temperature to delineate their practical thermal windows (Fig. 5a) [76]. Temperature-dependent analysis of AFZO and AZO revealed how resistivity, carrier concentration, and mobility evolve under air annealing, emphasizing the importance of thermal processing for maintaining plasmonic response (Fig. 5b). Corresponding transmission spectra confirm AFZO’s stability after 400–700 °C air anneals (typically for several hours), highlighting the roles of dense microstructures and controlled doping in preserving mid-infrared optical properties (Fig. 5c). Similarly, Barshilia et al. designed an ITO/Ag/ITO (IAI) spectral beam splitter for hybrid solar conversion (Fig. 5d), demonstrating selective reflection in the visible-near-infrared and full mid-infrared reflection (Fig. 5e) with minimal transmittance (Fig. 5f) [77]. These examples illustrate that conductive-oxide systems can reliably achieve static high-reflectance performance while resisting high-temperature degradation in oxygen-rich environments.

Extending toward active thermal management, Liu et al. fabricated a dynamic emissivity device on BaF_2_ (Fig. 5g), sequentially depositing Mg_x_Ni alloys with a 5 nm Pd layer. Applying -2.6 V induced a transition from low to high emissivity, achieving central temperature shifts of 5.1 °C and full switching within 150 s (Fig. 5h), with emissivity changes of 0.48 and 0.43 in the 3–5 and 7.5–14 μm bands, respectively (Fig. 5j) [78]. Further, a dielectric-insulator-emitter (DIE) regulator employing AZO nanocrystals modulates plasmonic absorption during electron extraction (Fig. 5k), maintaining high infrared reflectivity in the ITO capping layer and a low-emissivity state. The TDIE structure and Li^+^-ion charge-balancing mechanism are illustrated schematically (Fig. 5l)**,** while voltage-dependent visible transmittance and infrared emissivity spectra (with atmospheric windows highlighted) confirm the tunable optical response (Fig. 5m) [79]. Overall, conductive oxides are among the most promising low-emittance candidates in oxidizing environments up to ~ 800–1000 °C because of their oxidation tolerance and tunable carrier density; at higher temperatures, carrier depletion, defect-driven damping, and cation diffusion can restrict their stability compared with refractory metals in inert atmospheres.

### Transition Metal Nitrides

Transition metal nitrides achieve high mid-infrared reflectance through metallic d-band occupancy and refractory rock salt lattices that sustain dense free-carrier populations while delaying microstructural coarsening. TiN, ZrN, and HfN exemplify this coupling: partially filled d-states yield negative real permittivity across the MIR, with interband transitions positioned away from the working band [80, 81], while strong metal–nitrogen bonding helps preserve polished surfaces within the electromagnetic skin depth under cyclic heating. Performance stability hinges on how non-stoichiometry, grain boundaries, and oxygen ingress evolve. Nitrogen vacancies have a dual effect: moderate vacancy concentrations increase free-carrier density and strengthen the plasmonic response, whereas excessive vacancies act as defect-scattering centers, increase the damping parameter γ, reduce mobility, and convert carrier regulation into optical loss. Columnar grains formed during reactive deposition can accelerate oxygen transport, and boundary grooving introduces Mie-scale features that convert specular reflection into hemispherical loss. Oxidation chemistry sets the environmental ceiling: TiN tends to form porous TiO_2_ scales with rapid reflectance loss, whereas ZrN and HfN form denser zirconia and hafnia that slow but do not eliminate oxide thickening under sustained oxygen potential. Alloying can moderate this trade-off: Al additions improve oxidation resistance through adherent Al_2_O_3_ but may dilute the metallic network, while Ta or Nb can tune vacancy formation energies and carrier mobility. Residual stress, thermal expansion mismatch, and impurity segregation further influence cracking, delamination, and optical loss. Under inert or vacuum conditions, dense TiN, ZrN, and HfN coatings can maintain low emittance well beyond [80]; in moderately oxidizing atmospheres, stable performance requires tight stoichiometry control, stabilized texture, and passivating scales thinner than the skin depth. Thus, transition metal nitrides offer a practical balance of plasmonic response, refractory bonding, and process compatibility for MIR reflectors under demanding thermal loads.

**Fig. 6:**
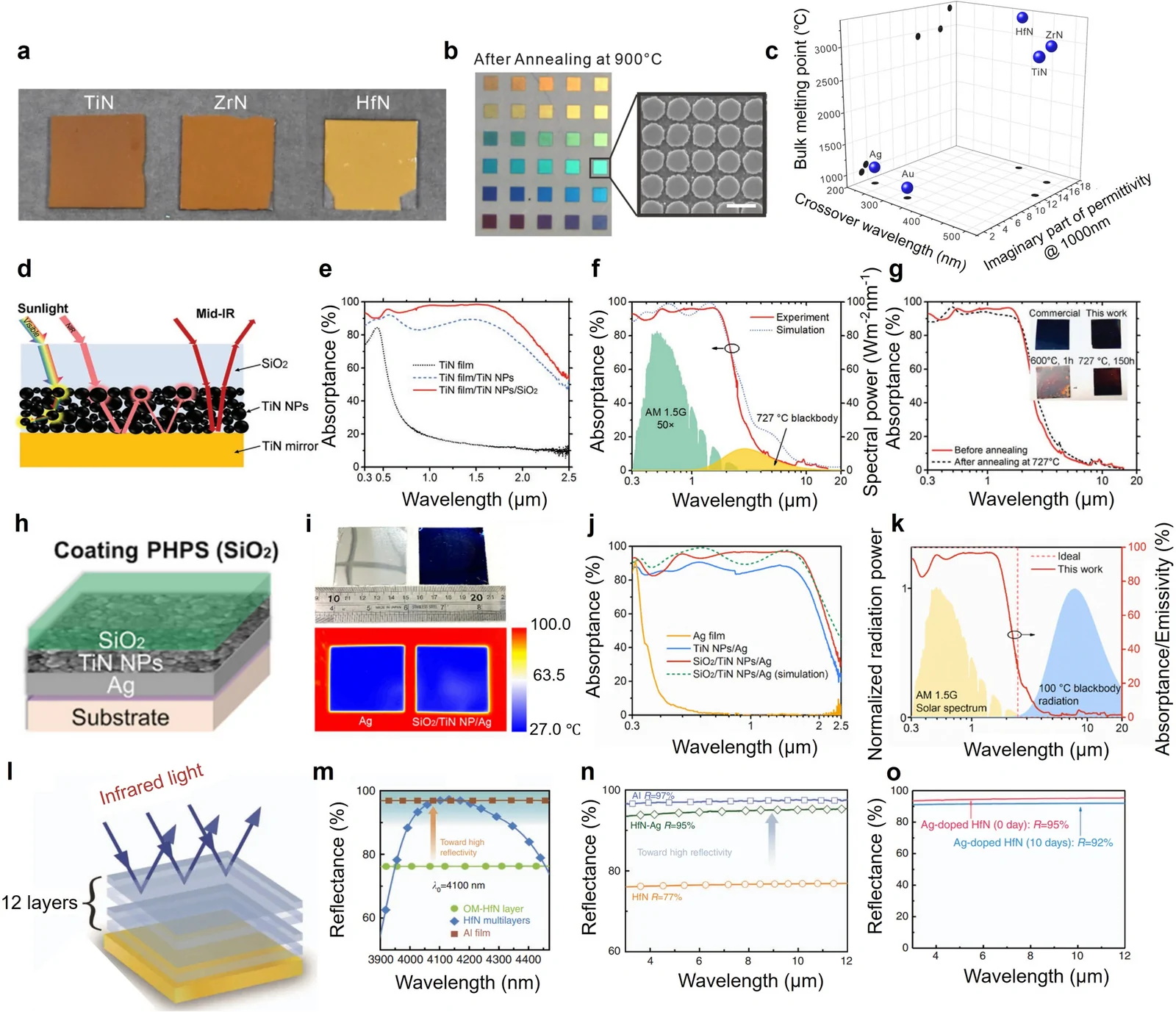
High-temperature-resistant MIR transition metal nitrides. **a** TiN, ZrN, HfN films on sapphire. **b** HfN morphology after annealing. **c** Plasmonic resonance and high-temperature stability of TiN, ZrN, and HfN. Reproduced with permission [81]. Copyright 2022, De Gruyter. **d** TiN nanoparticle absorber design. **e** Absorptance spectra of TiN films with TiN nanoparticles and SiO_2_ coating. **f** UV–visible-IR absorptance spectra of SSA compared with simulations and solar/blackbody spectra. **g** Structural stability of absorbers after thermal cycling. Reproduced with permission [82]. Copyright 2020, John Wiley and Sons. **h** Fabrication process of the NoM-PMA. **i** Optical and infrared imaging of thermal shielding performance. **j** Measured and simulated absorptance of NoM-PMA. **k** Absorptance/emissivity of SiO_2_/TiN NP/Ag structure. Reproduced with permission [83]. Copyright 2022, American Chemical Society. **l** Design schematic of multilayer hafnium-nitride-only films. **m** Reflectance spectra of single-layer and multilayer films. **n** Reflectance spectra of pure and doped HfN films. **o** Reflectance of Ag-doped HfN before and after environmental exposure. Reproduced with permission [84]. Copyright 2017, Springer Nature

Transition metal nitrides, leveraging refractory rock salt lattices and metallic *d*-band carriers, have been extensively explored as high-temperature mid-infrared reflectors. Lu et al. deposited TiN, ZrN, and HfN films on sapphire substrates via RF reactive magnetron sputtering at 800 °C **(**Fig. 6a**)**, demonstrating that HfN nanodisk arrays retain their morphology and spectral reflectance under near-normal incidence even after annealing up to 900 °C **(**Fig. 6b**)**. The optical response, obtained from microspectrophotometer-based reflection measurements under ambient conditions, highlights their superior thermal stability compared to noble metals such as Ag and Au **(**Fig. 6c**)** [81]. Building on this advantage, Huang et al. fabricated a plasmonic selective absorber by assembling ultrathin (~ 120 nm) TiN nanoparticles onto a TiN mirror via a solution-processed route (Fig. 6d) [82]. Spectral measurements reveal enhanced absorption compared to bare TiN, and a SiO_2_ encapsulation layer further modulates the broadband response (Fig. 6e). The TiN-based selective solar absorber suppresses mid-IR emission to below ~ 3%, as derived from spectrally averaged emittance at long wavelengths **(**Fig. 6f**)**, and maintains stable absorptance during thermal cycling up to 727 °C over extended durations, demonstrating robust low-emissivity operation at elevated temperatures **(**Fig. 6g**)**. In parallel, a non-metallic plasmonic meta-absorber (NoM-PMA) combining Ag ink, TiN colloidal nanoparticles, and a SiO_2_ precursor achieves effective thermal-radiative shielding **(**Fig. 6h**)**, maintaining apparent temperatures near room temperature despite an actual temperature of 100 °C **(**Fig. 6i**)**. The measured and simulated spectral absorptance show good agreement, validating the optical design of the TiN nanoparticle-based absorber **(**Fig. 6j**)**. Based on this architecture, the SiO_2_/TiN NP/Ag metamaterial exhibits high spectrally averaged solar absorptance (~ 94%) together with extremely low integrated thermal emissivity (~ 2%), approaching the performance of an ideal solar absorber **(**Fig. 6k**)** [83].

Further exploiting nitride tunability, Hu et al. engineered HfN-only multilayer stacks (OM/(LT/HT)_z_) with carefully designed layer thicknesses **(**Fig. 6l**)** and refractive indices to satisfy mid-infrared interference conditions (*λ*_0_ = 4100 nm) [84], substantially enhancing reflectivity of the outermost layer **(**Fig. 6m**)**. Comparative studies show that Ag-doped HfN achieves ~ 95% average reflectance across 3–12 µm and exhibits minimal loss (~ 3%) after thermal exposure (Fig. 6n**)**, indicating that controlled doping stabilizes MIR optical constants without compromising refractory performance (Fig. 6o). Overall, transition metal nitrides provide a balanced trade-off between plasmonic response and thermal stability, enabling operation around ~ 900–1000 °C in moderately oxidizing environments; however, progressive nitride-to-oxide transformation limits long-term stability compared with conductive oxides in air and refractory metals in inert conditions.

### MXene Family

MXene materials, exemplified by Ti_3_C_2_T_x_, Nb_2_C, and Mo_2_C derivatives from layered MAX phases, couple high in-plane electrical conductivity with strong dielectric anisotropy and can express MIR reflectance when films are dense, smooth, and highly oriented [85, 86]. Their response originates from partially filled metallic d-band states, while surface terminations (T=O, OH, F) regulate carrier concentration, scattering, work function, and plasmonic damping relative to the MIR band. Structural stability under heat depends on lamellar alignment, porosity, termination chemistry, and atmosphere [87, 88]. Aligned lamellae reduce interflake resistance and confine current within conductive basal planes, whereas densification suppresses Mie-scale scattering and apparent emissivity increase [89]. Termination chemistry is the dominant high-temperature variable: F and OH groups desorb or transform first, shifting surfaces toward O termination; this may reduce disorder-related resistance in inert atmospheres but can accelerate oxidation along interlayer pathways in oxygen-bearing environments [90, 91]. Titanium-based MXenes illustrate the main trade-off: moderate inert heating can heal etch-induced defects and improve conductivity [92–94], whereas prolonged oxygen exposure drives oxycarbide or oxide formation, roughening, and reflectance loss. Grain coarsening and interlayer sintering may initially reduce junction resistance and enhance specular reflectance [95], but excessive coalescence introduces surface undulations that convert mirror-like reflection into hemispherical loss. Therefore, effective high-temperature MXene reflectors require stable termination populations, dense basal-plane texture, and protected atmospheres; their processing flexibility is attractive, but their atmosphere window remains narrower than that of conductive oxides or noble-metal systems [96, 97].

**Fig. 7:**
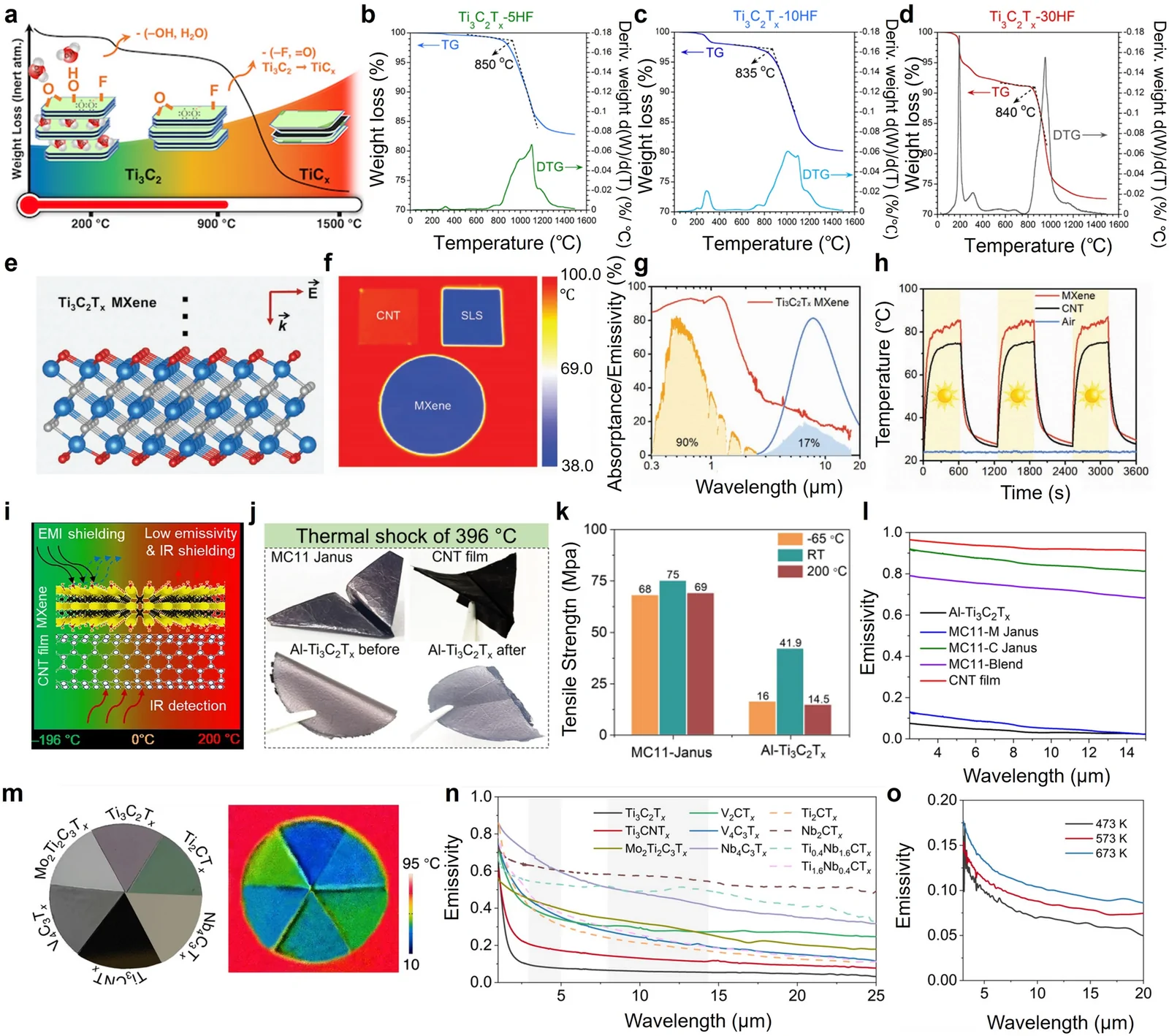
High-temperature-resistant MIR MXene family. **a** Phase evolution of Ti₃C₂ under inert heating. **b**-**d** TG-DTG under different etching conditions. Reproduced with permission [98]. Copyright 2019, American Chemical Society. **e** Layered molecular structure of bulk Ti_3_C_2_T_x_ film. **f** Infrared images of CNT, SLS, and MXene films. **g** Absorptance/emissivity spectra of different absorber systems. **h** Temperature–time response under illumination. Reproduced with permission [11]. Copyright 2021, John Wiley and Sons. **i** Structure of Janus MXene-based composite. **j** Morphology before and after thermal shock. **k** Tensile strength of MXene-based composites. **l** Emissivity spectra of MXene-based films. Reproduced with permission [52]. Copyright 2024, Springer Nature. **m** Visible/IR images of MXene coatings. **n** Emissivity spectra of different MXene compositions. **o** Temperature-dependent emissivity of Ti_3_C_2_T_x_ coatings. Reproduced with permission [99]. Copyright 2023, Elsevier

Building on these structural and chemical principles, several studies have systematically examined the high-temperature MIR performance of MXenes. Gogotsi et al. demonstrated that post-etching chemistry, rather than etching severity, governs the mid-infrared response and thermal stability of Ti_3_C_2_T_x_ films (Fig. 7a). Free-standing Ti_3_C_2_T_x_ films derived from Ti_3_AlC_2_ etched with 5–30 wt% HF retain structural integrity under inert heating up to ~ 740 °C, accompanied by gradual dissociation of surface terminations, whereas pronounced degradation occurs above ~ 830 °C. This behavior is supported by thermal stability and phase evolution analysis of Ti_3_C_2_, together with TG-DTG curves of Ti_3_C_2_T_x_ etched with 5 (Fig. 7b), 10 (Fig. 7c), and 30 wt% HF (Fig. 7d)**,** which reveal comparable thermal responses despite varying etching concentrations [98]. Huang et al. visualized the layered architecture of bulk Ti_3_C_2_T_x_ films and demonstrated that dense ~ 15-µm Ti_3_C_2_T_x_ absorbers simultaneously achieve high solar absorptance and low mid-infrared absorption (Fig. 7e), resulting in enhanced solar-to-thermal conversion efficiency and a surface temperature rise of 62 °C, outperforming carbon nanotube (CNT) absorbers. This performance advantage is corroborated by infrared images of selective layered structures (SLS), CNT, and MXene films on a 100 °C hot plate (Fig. 7f), together with absorptance/emissivity spectra benchmarked against the solar and blackbody spectra (Fig. 7g), and the temperature–time profiles of CNT and MXene absorbers under 1 sun illumination (Fig. 7h) [11]. Koo et al. further reinforced MXene mechanical robustness by combining Ti_3_C_2_T_x_ with CNT films in a Janus architecture (Fig. 7i). Under Δ396 °C thermal shock (Fig. 7j), the MC11-Janus composite preserved flexibility and tensile strength significantly better than Al- Ti_3_C_2_T_x_ films (Fig. 7k). Room temperature emissivity spectra of CNT, Al–Ti_3_C_2_T_x_, MC11-C, MC11-M, and MC11-Blend films further highlight that hybrid lamination provides an effective route to stabilize MXenes under extreme environments (Fig. 7l) [52].

Gogotsi et al. report that the MXene family of two-dimensional carbides and carbonitrides exhibits a broad range of infrared emissivity values accompanied by diverse colors, which vary with MXene composition and structure. This diversity is visually summarized in a six-segment MXene “color palette” displayed on PET, providing a convenient representation of composition-dependent IR and optical properties (Fig. 7m). Infrared emissivity spectra of MXene coatings, comparing solid-solution Mxenes (Fig. 7n). Temperature-dependent infrared emissivity of Ti_3_C_2_T_x_ coatings measured from 473 to 673 K, confirming the high-temperature operational window with sustained low emittance (Fig. 7o) [99]. Together, these studies show that termination and microstructural control enable MXenes as tunable MIR-reflective coatings, while mechanical reinforcement improves their robustness. However, their stability is atmosphere-sensitive: Ti_3_C_2_T_x_-based MXenes can remain stable at ~ 700–800 °C in inert environments, but oxidation and termination evolution in air cause rapid reflectance degradation. Thus, MXenes are best suited to moderate temperature or protected conditions, whereas metals are favored in inert environments, conductive oxides in oxidizing atmospheres, and nitrides in intermediate refractory regimes.

## High-Temperature-Resistant Mid-Infrared High-Absorptance (High-Emittance) Materials

**Fig. 8:**
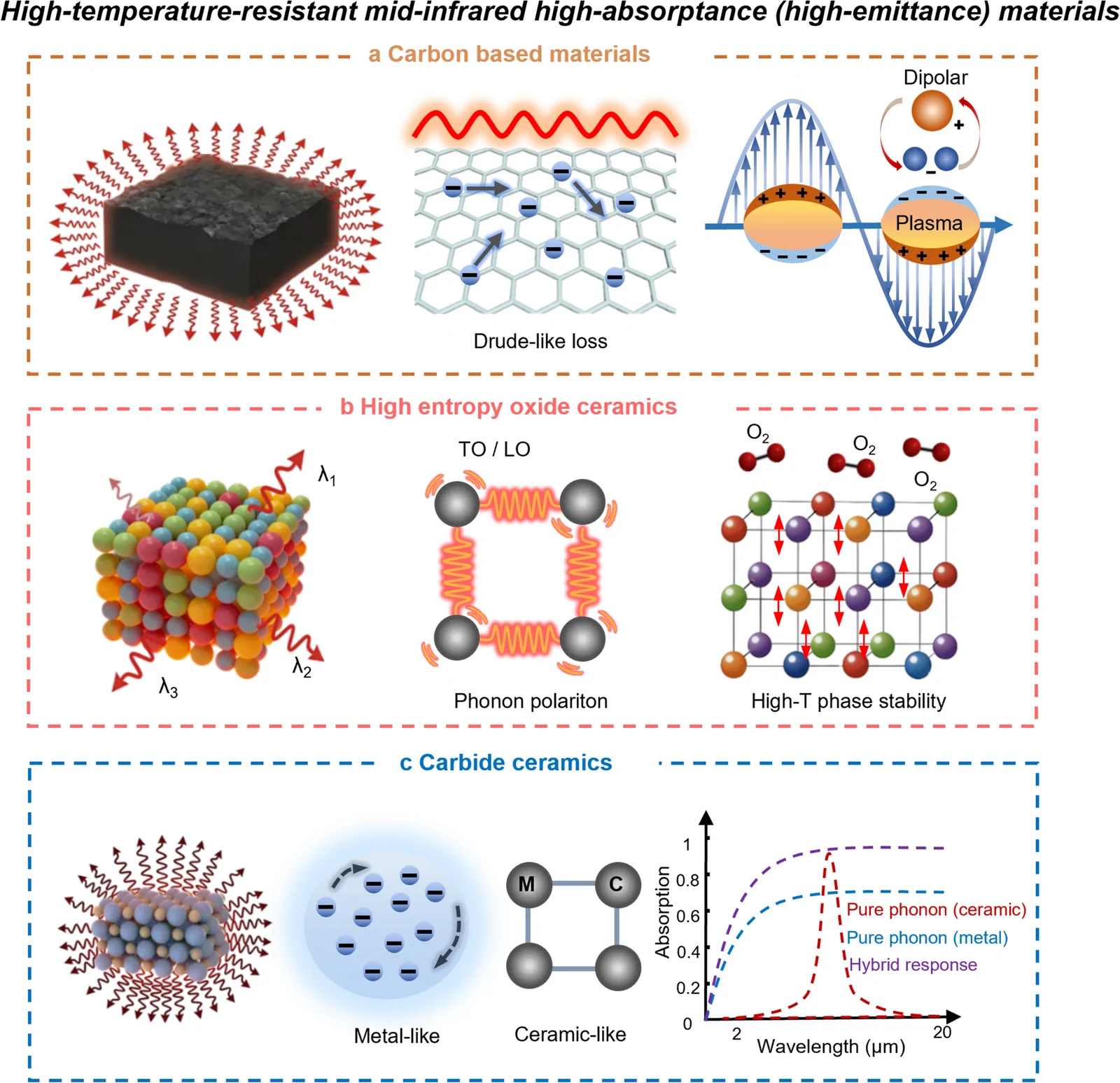
High-temperature-resistant mid-infrared high-absorptance (high-emittance) materials. **a** Carbon-based materials: free-carrier and defect-assisted loss for near-gray broadband emittance. **b** High-entropy oxide ceramics: polar-lattice phonon modes and Reststrahlen-related selective emission. **c** Carbide ceramics: hybrid metallic/ceramic absorption for wide-band high-temperature radiation

High-temperature-resistant mid-infrared high-absorptance/high-emittance materials enable radiative heat exchange by enhancing MIR loss channels while retaining structural and chemical stability at service temperature (Fig. 8). Rather than providing an exhaustive catalog, this section focuses on representative families that capture the main emission mechanisms and degradation limits under realistic thermal and environmental conditions. Two archetypal mechanisms dominate: conductive systems, including carbons and some ceramics, convert free-carrier scattering into broadband emittance governed by carrier density, mobility, and near-surface defect scattering [100], whereas polar lattices in oxide and carbide ceramics concentrate oscillator strength into Reststrahlen windows through phonon–polariton coupling [101]. Their durability depends on defect equilibria, phase stability, oxidation kinetics, porosity, and surface evolution, because these factors can shift emissivity away from the designed spectral window or introduce uncontrolled broadband loss [102, 103]. Practical selection should therefore couple the target 3–5 and 8–12 μm windows with atmosphere and thermal shock constraints, prioritizing dense microstructures, stable loss mechanisms, and chemistries that resist conversion into insulating or glassy scales. Accordingly, carbon-based materials are emphasized for near-gray broadband emittance, oxide ceramics for stable emission in oxidizing environments, and carbide ceramics for ultrahigh-temperature radiators under inert or controlled atmospheres [104, 105]. Meaningful comparison should consider not only spectral emissivity, but also whether the underlying loss mechanism remains stable during thermal exposure.

### MXene Family

Carbon-based materials provide high MIR absorptance through delocalized *π*-electron conduction, vibrational loss, and hierarchical light-trapping structures [106, 107]. Graphite represents the intrinsic mechanism: stacked sp^2^ layers support in-plane free-carrier damping at MIR frequencies, while weaker out-of-plane transport introduces anisotropic apparent emittance [108, 109]. Turbostratic carbons, glassy carbon, porous foams, felts, and carbon nanotube assemblies further increase broadband absorptance through defect scattering, phonon coupling, multiple scattering, and extended optical paths [110, 111]. Their high-temperature durability originates from strong covalent bonding and high sublimation temperature under inert or vacuum conditions, but the high surface area that enhances absorption also accelerates oxidation in air, causing mass loss, roughening, and uncontrolled emissivity drift [112–114]. Therefore, stable carbon emitters require graphitic ordering, controlled porosity, low reactive-edge density, and surface features smaller than the MIR wavelength to preserve designed spectra during thermal cycling [115–117]. Overall, carbon-based systems are most suitable as broadband high-emittance coatings in inert or vacuum environments, whereas sustained operation in oxidizing atmospheres requires oxidation protection or hybrid ceramic architectures.

**Fig. 9:**
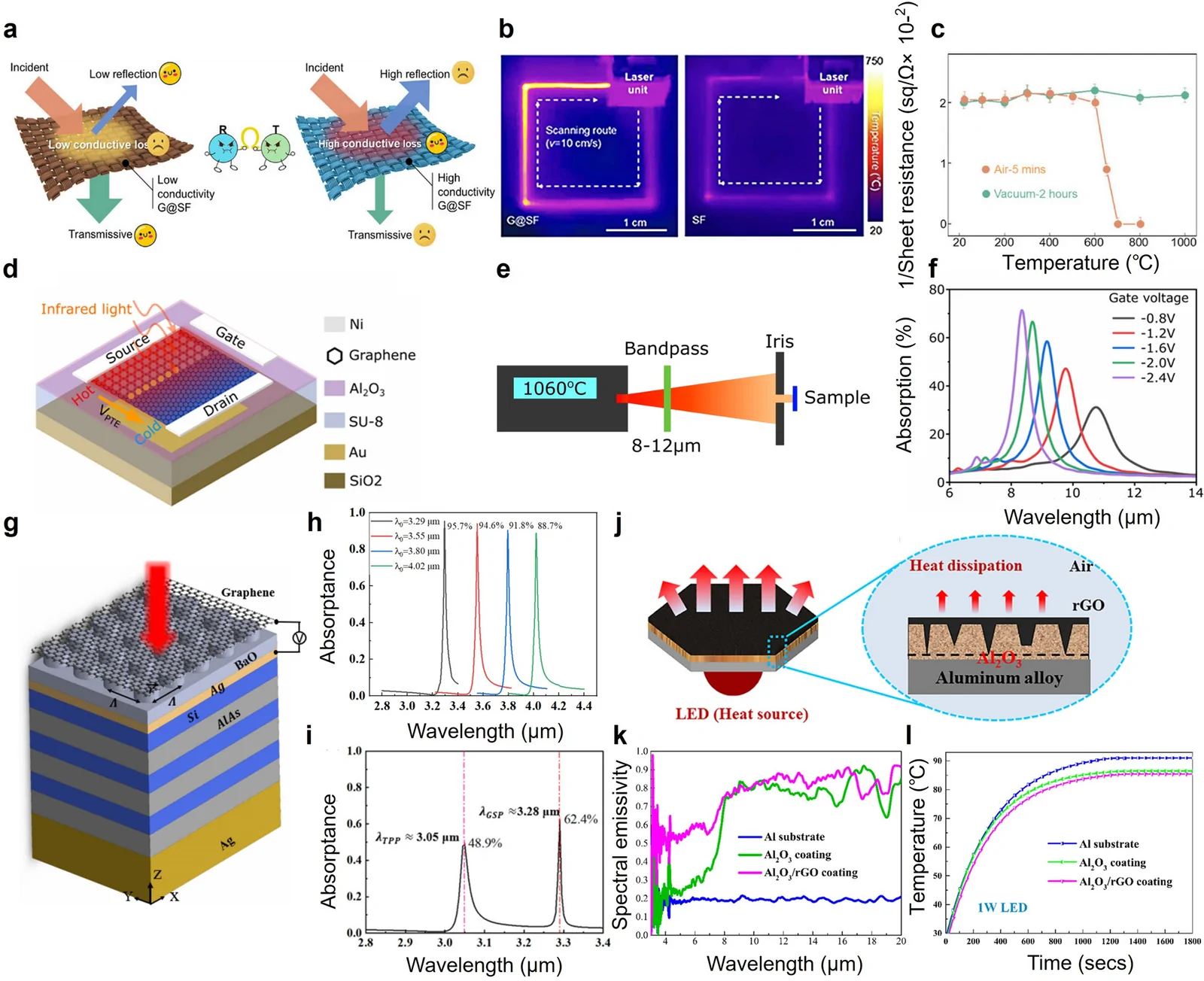
High-temperature-resistant MIR high-absorptance carbon-based materials. **a** Conductive‐loss and reflectance trade-off as a function of G@SF conductivity. **b** Infrared imaging of G@SF and SF during laser-induced erasing. **c** Sheet resistance of G@SFM after thermal treatments in vacuum and air. Reproduced with permission [12]. Copyright 2025, John Wiley and Sons. **d** Schematic of photothermoelectric detector generating VPTE under infrared illumination. **e** Optical measurement setup using a blackbody radiation source. **f** Simulated absorption spectra of patterned graphene. Reproduced with permission [116]. Copyright 2024, American Chemical Society. **g** Schematic of the proposed resonant absorber structure. **h** Absorption spectra at different wavelengths. **i** Absorbance spectra of the proposed design under multiple operating conditions. Reproduced with permission [118]. Copyright 2025, Elsevier. **j** Schematic of LED with double-layer thermal/optical coating. **k** Infrared emissivity of Al alloy, Al_2_O_3_ layer, and Al_2_O_3_/rGO coating. **l** Temperature response of coated systems. Reproduced with permission [119]. Copyright 2018, Elsevier

Building on these mechanistic insights, several experimental studies have explored the mid-infrared performance of carbon-based materials under high-temperature conditions. Liu et al. developed flexible graphene-on-silica-fabric (G@SF) architectures through controlled pyrolysis and carbon precursor reorganization (Fig. 9a), demonstrating localized heating up to 700 ± 25 °C and thermal robustness under spatially non-uniform temperature fields (Fig. 9b) [12]. Thermal treatments from 20 to 1000 °C in vacuum and air further revealed structural stability in inert atmospheres and the onset of oxidation-driven degradation in air (Fig. 9c). Chanda et al. fabricated a large-area long-wave infrared (LWIR) detector using CVD-grown graphene, demonstrating voltage-controlled tunability of mid-infrared detectivity (D*) and gate-adjustable absorption across 6–14 µm (Fig. 9d). The photothermoelectric detector generates a voltage (VPTE) under infrared illumination, as schematically illustrated, and its optical response was characterized using a blackbody radiation source (Fig. 9e). Simulated absorption spectra of gate-tuned cavity-coupled graphene with hexagonal hole arrays provide theoretical insight into the wavelength-selective absorption behavior. (Fig. 9f) [116]. These works confirm that graphene-based platforms can provide actively tunable MIR functionality while maintaining structural integrity, highlighting their potential as adaptive radiative elements.

Extending toward engineered MIR absorption and practical thermal management coatings, Ren et al. integrated graphene-BaO cylinder arrays with Ag-DBR stacks (Fig. 9g), achieving wavelength-selective absorption peaks up to 96% and elucidating transverse photonic-plasmonic (TPP) mode excitation (Fig. 9h, i) [118]. Similarly, Zhou et al. constructed dual-layer Al_2_O_3_/rGO composites (Fig. 9j) to enhance mid-infrared emissivity (Fig. 9k), demonstrating substantial radiative output and improved heat dissipation on 1 W LED chips, reducing operating temperature by 5.6 °C relative to bare aluminum (Fig. 9l) [119]. Together, these studies show that carbon-based and graphene-derived materials offer broadband MIR absorptance, tunable spectral response, and high-temperature durability for radiative cooling and adaptive thermal management. They are most effective as high-emittance coatings in inert or vacuum environments up to ~ 1000 °C, where stable covalent networks sustain radiative loss. In air, however, rapid oxidation and structural degradation limit long-term stability, making oxide or carbide ceramics more suitable for sustained high-temperature operation.

### High-Entropy Oxide Ceramics

High-entropy oxide (HEO) ceramics introduce an additional design paradigm for high-temperature mid-infrared materials by exploiting multi-cation mixing to stabilize single-phase lattices through large configurational entropy [120, 121]. The random distribution of multiple cations on equivalent lattice sites suppresses long-range diffusion, slows grain coarsening, and elevates activation barriers for phase separation, collectively enhancing structural integrity at extreme temperatures [122]. This entropic stabilization also reduces oxygen vacancy mobility, thereby improving oxidation resistance and maintaining consistent optical response in oxygen-rich environments where conventional oxides often undergo volatilization or sub-oxide formation. In the mid-infrared regime, HEOs retain strong phonon–polariton resonances characteristic of polar lattices; however, the multi-cation disorder broadens phonon density-of-states distributions, enabling tunable [123], and often broadened, Reststrahlen bands. This broadening can enhance spectral coverage for thermal emission or, conversely, be engineered for narrowband applications through targeted cation selection. Furthermore, suppressed thermal conductivity in HEOs reduces near-surface temperature gradients and mitigates thermally induced defect evolution, contributing to spectral stability during cyclic heating. Together, these features position high-entropy oxides as a promising class of materials for high-temperature MIR emission, selective radiative cooling, and thermally stable MIR transmission components, offering a robust foundation for the subsequent discussion of state-of-the-art research in this domain [124, 125].

**Fig. 10:**
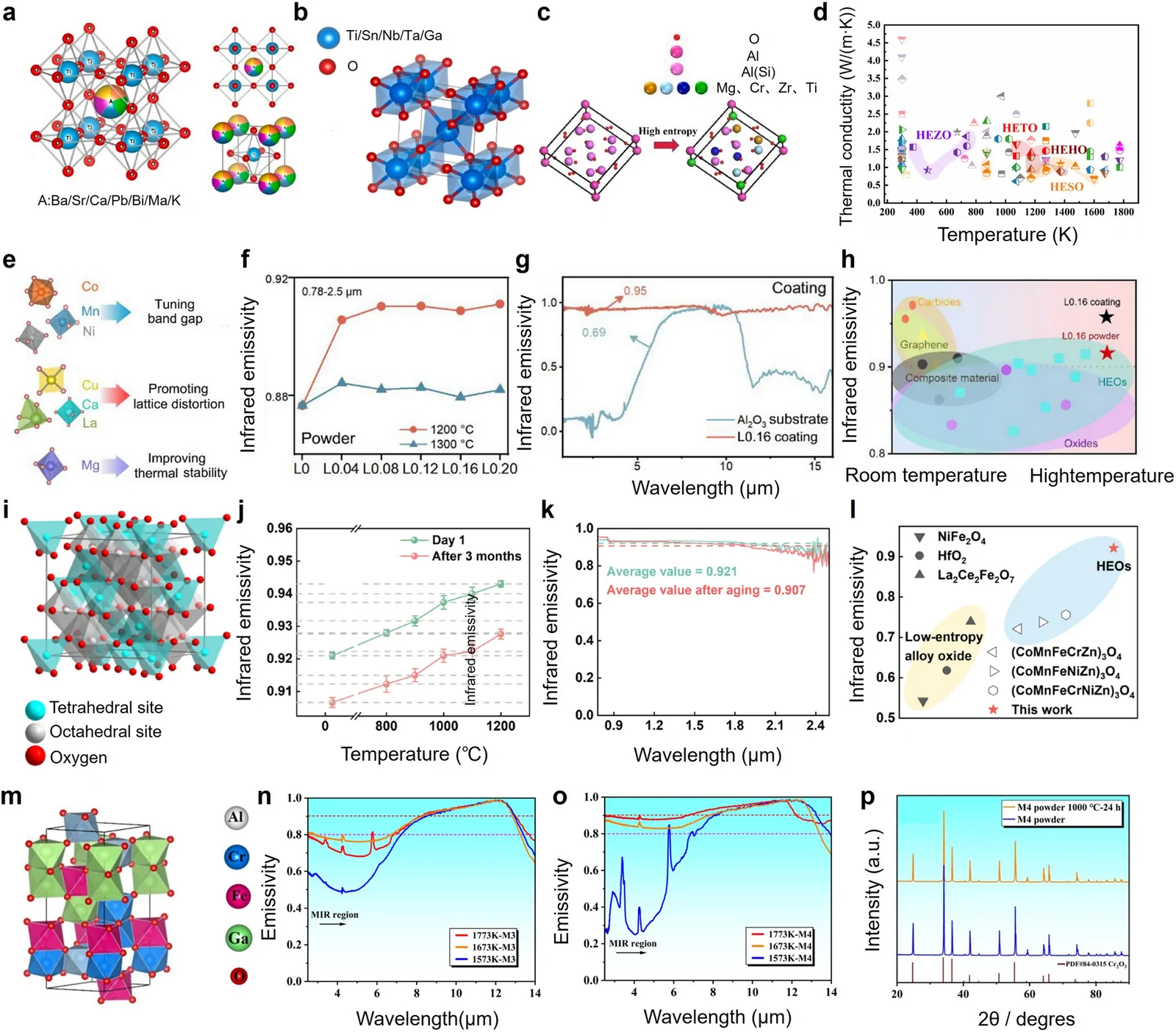
High-temperature-resistant MIR high-absorptance high-entropy oxide ceramics. **a** Unit-cell structure of perovskite. **b** Crystal structure of rutile. **c** Crystal structure of mullite. **d** Thermal conductivity comparison across ceramics with diverse crystal structures. Reproduced with permission [123]. Copyright 2025, Elsevier. **e** Cation-selection strategy for band gap control and lattice stabilization. **f** Emissivity evolution with synthesis conditions. **g** Infrared spectra of oxide coatings. **h** Broadband emissivity comparison with leading emitter materials. Reproduced with permission [126]. Copyright 2025, John Wiley and Sons. **i** Lattice framework of spinel GheOs. **j** Temperature-dependent infrared emissivity of spinel GheOs. **k** Infrared emissivity of spinel GheOs. **l** Emissivity comparison between spinel GheOs and various metal oxides. Reproduced with permission [127]. Copyright 2025, AAAS. **m** DFT-calculated crystal structures of the M4 composition. **n** MIR emissivity spectra of M3 synthesized at different temperatures. **o** MIR emissivity spectra of M4 synthesized at different temperatures. **p** XRD patterns before and after annealing. Reproduced with permission [50]. Copyright 2024, Elsevier

Building on these entropic-stabilization principles, several recent studies have translated high-entropy oxide design into experimentally realized mid-infrared performance. Qiao et al. illustrated the perovskite lattice framework supporting multi-cation substitution and constructed high-entropy rutile ceramics, forming thermally robust platforms suitable for MIR applications (Fig. 10a, b) [123], while classical mullite structures provided comparative context for thermal transport behavior (Fig. 10c, d). Gao et al. introduced transition metal ions (Co, Ni, Mn) to engineer electronic structures (Fig. 10e) and enhance MIR emissivity, achieving values from 0.85 to 0.91 within the 0.78–2.5 µm range (Fig. 10f, g), with slight decreases at higher synthesis temperatures due to interconnected porosity promoting multiple scattering (Fig. 10h) [126]. Zhou et al. extended these strategies to spinel-type HEOs (Fig. 10i), demonstrating that multi-cation disorder preserves the spinel framework while stabilizing infrared emissivity at elevated temperatures (Fig. 10j). This behavior is evidenced by the temperature-dependent infrared emissivity of spinel HEOs and their emissivity spectra in the 0.75–2.5 µm range (Fig. 10k)**,** confirming robust optical performance under thermal stress (Fig. 10l) [127]. These results confirm that high-entropy oxide composition and lattice design jointly govern emissivity, thermal stability, and structural integrity, providing a robust foundation for practical MIR materials.

Further diversifying the high-entropy oxide portfolio, Shao et al. investigated Al_2_O_3_-, Cr_2_O_3_–, Fe_2_O_3_–, and Ga_2_O_3_-derived compositions (Fig. 10m), achieving MIR emissivity of 0.933–0.957 in the 8–14 µm band at 1573–1773 K (Fig. 10n, o) [50]. XRD confirmed phase stability after 1000 °C annealing for 24 h, demonstrating excellent high-temperature resilience (Fig. 10p) [50]. Together, these studies highlight that multi-cation disorder not only broadens phonon modes and enhances radiative loss channels but also stabilizes mid-infrared optical properties, establishing high-entropy oxides as promising candidates for high-temperature MIR emission, selective radiative cooling, and thermally robust optical components. Overall, high-entropy oxide ceramics are most promising for high-emittance applications in oxidizing environments up to ~ 1500–1700 °C, where their entropy-stabilized lattice suppresses phase degradation and maintains emissivity stability. However, their broadened phonon response limits spectral selectivity, making them less suitable than engineered photonic structures for applications requiring narrowband emission control.

### Carbide Ceramics

Carbide ceramics deliver high mid-infrared absorptance through metallic or strongly hybridized electronic structures coupled with ultrahigh-temperature covalent-ionic lattices that preserve near-surface loss channels under severe thermal load [128]. Transition metal carbides such as HfC, ZrC, TiC, and TaC exhibit dense conduction bands with appreciable free-carrier damping, which produces broadband emittance that remains effective as temperature rises because carrier scattering increases and interband activity stays largely outside the working window [129, 130]. Silicon carbide provides a complementary case in which strong polar bonding concentrates oscillator strength into Reststrahlen features while residual carriers and defect states add a controllable broadband background [131, 132]. Their durability arises from high melting points, high elastic moduli, and sluggish diffusion, which suppress creep, grain coarsening, and roughening within the optical skin-depth region. Stoichiometry and impurity control remain critical: carbon vacancies or oxygen contamination may increase electronic loss but can also promote embrittlement, substoichiometric domains, and rough oxide scales during exposure. Oxidation chemistry defines the usable atmosphere window: HfC and ZrC form refractory oxides that slow further reaction but may become porous and scattering, TiC generates mixed TiO_x_ and volatile species that degrade surface quality, whereas SiC forms silica that can stabilize emission until volatilization or stress cracking occurs in fast-flowing or low-pressure environments [133, 134]. Dense microstructures, suppressed second phases, narrow grain-size distributions, and polished surfaces are therefore required to convert intrinsic loss into stable high emittance while limiting Mie scattering and oxide-driven roughness growth. Under inert or controlled atmospheres, these attributes yield near-gray, high-emittance radiators that tolerate rapid cycling and extreme heat flux; in oxidizing environments, reliable operation requires compact, stable oxide scales so that the optical response remains governed by the carbide substrate rather than by a lossy scattering overlayer [101, 135, 136].

**Fig. 11:**
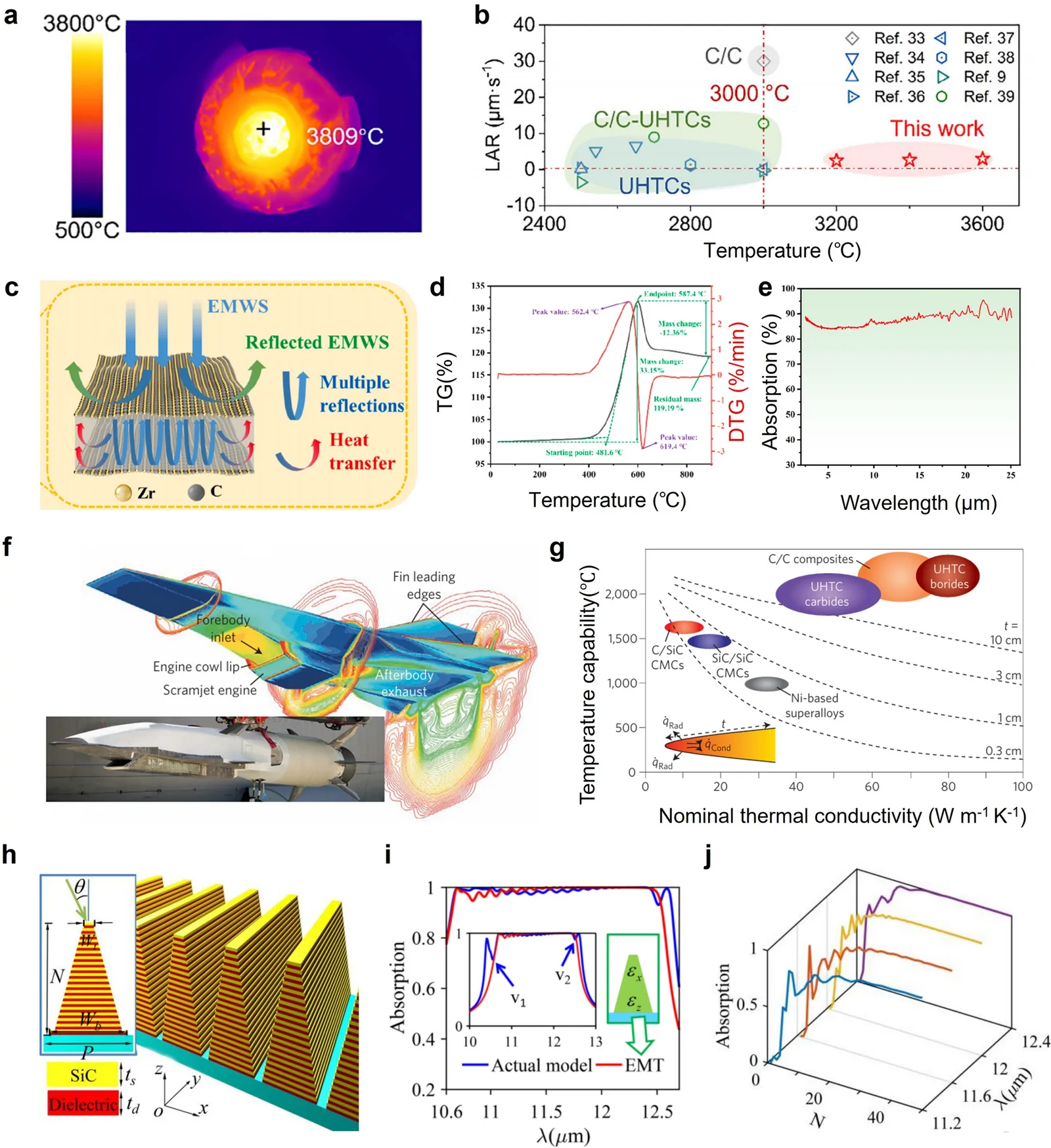
High-temperature-resistant MIR high-absorptance carbide ceramics. **a** Temperature–time evolution during laser oxidation measured by an IR thermometer. **b** Oxidation resistance comparison between HEC-W and other ultrahigh-temperature materials. Reproduced with permission [130]. Copyright 2025, John Wiley and Sons. **c** Schematic of the electromagnetic shielding mechanism. **d** Thermogravimetric and phase analysis of ZrC. **e** Infrared absorption characteristics of ZrC. Reproduced with permission [104]. Copyright 2025, Elsevier. **f** CFD simulation of hypersonic thermal environment. **g** Ashby map of temperature capability and thermal conductivity. Reproduced with permission [135]. Copyright 2016, Springer Nature. **h** Geometry of multilayer absorber structure. **i** Absorption spectra of metamaterial absorbers. **j** Effect of material selection on absorption behavior. Reproduced with permission [101]. Copyright 2025, MDPI

Building on these mechanistic insights, several studies have explored high-temperature MIR performance of carbide ceramics. Chu et al. synthesized multicomponent (Hf,Ta,Zr,W)C high-entropy carbides (HECs) [137], achieving remarkably low oxidation rates of 2.7 μm s^−1^ up to 3600 °C (Fig. 11a), demonstrating that severe lattice distortion and sluggish diffusion preserve carbide integrity under extreme heat flux. Their comparison of HEC-W with conventional ultrahigh-temperature ceramics, C/C composites, and hybrid C/C-UHTC structures (Fig. 11b) highlights the superior durability of entropy-engineered carbides [130]. Chen et al. analyzed ZrC films, proposing a carrier damping-dominated MIR absorption mechanism **(**Fig. 11c**)**. Thermogravimetric analysis revealed active oxidation transitions at 481.6 and 587.4 °C **(**Fig. 11d**)**, indicating the onset of oxidation without detailed kinetic rates. The ZrC films exhibit strong spectral absorptance over the 2.5–25 μm range, with an average of ~ 86% **(**Fig. 11e**)**, confirming their suitability for broadband MIR emission under harsh conditions [104]. Padture et al. mapped operational thermal scenarios for hypersonic flight, emphasizing carbides high thermal capability relative to superalloys and oxide CMCs (Fig. 11f, g) [135], while Qing et al. designed SiC/dielectric multilayer metamaterial absorbers achieving 53.3%–98.0% absorption efficiency via interface-guided cavity modes and phonon–polariton resonances (Fig. 11h), illustrating photonic-architected strategies for extreme environments (Fig. 11i, j) [101]. Together, these studies show that carbide ceramics couple electronic-structure-driven MIR absorption with exceptional thermal stability, while oxidation resistance, microstructure, and photonic design jointly determine performance. Overall, carbide ceramics are most suitable for ultrahigh-temperature high-emittance applications above ~ 1500 °C in inert or controlled atmospheres, where strong bonding and high melting points preserve radiative performance. In air, their long-term reliability depends on the compactness and stability of surface oxide scales, making oxide ceramics more reliable for sustained oxidizing operation.

## High-Temperature-Resistant Mid-Infrared High-Transmittance Materials

**Fig. 12:**
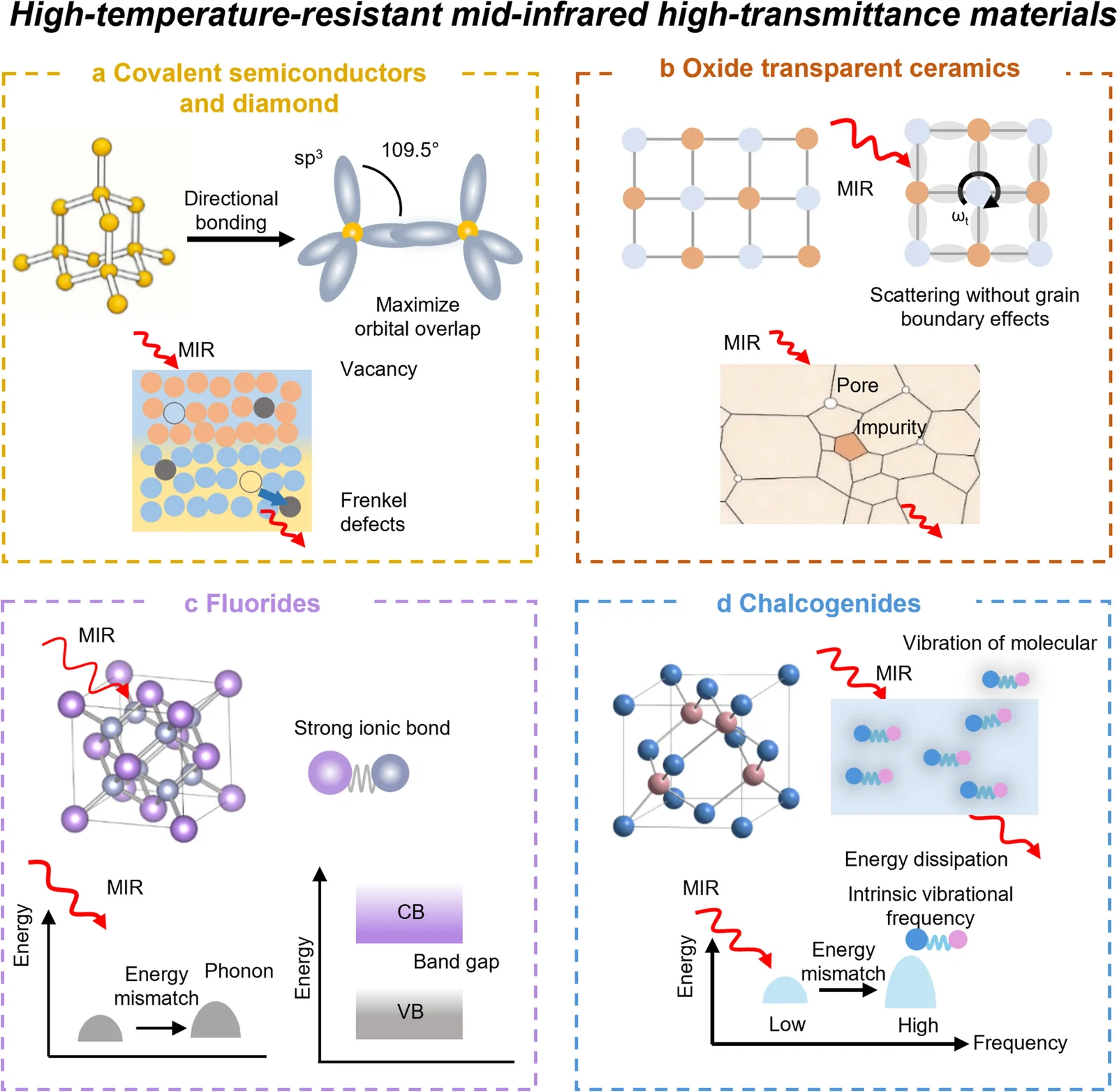
High-temperature-resistant mid-infrared high-transmittance materials. **a** Covalent semiconductors and diamond: strong bonding and high thermal transport for high-heat-flux MIR optics. **b** Oxide transparent ceramics: dense microstructures and oxidation resistance for 3–5 μm transmission. **c** Fluorides: extended MIR windows limited by temperature-humidity stability. **d** Chalcogenides: broad 8–12 μm transmission constrained by thermomechanical durability

High-temperature-resistant mid-infrared high-transmittance materials must suppress intrinsic absorption and extrinsic scattering while preserving mechanical integrity as temperature and atmosphere vary (Fig. 12). Rather than covering all transparent materials, this section focuses on representative systems that define the main transmission limits and degradation pathways under high-temperature operation [138]. The governing limits arise from multi-phonon absorption that sets the long-wavelength cutoff, free-carrier absorption from dopants and defects, and Mie-scale scattering from pores, inclusions, and rough surfaces. Reliable transmission therefore requires weak multi-phonon overlap in the target band, low free-carrier populations, near-theoretical density, clean grain boundaries, and surfaces resistant to oxidation, hydration, volatilization, and thermal shock-induced roughening. Within this framework, covalent semiconductors and diamond offer strong bonding and high thermal transport for high-heat-flux optics [139]; Oxide transparent ceramics provide robust 3–5 μm transmission and oxidation resistance; fluorides extend toward longer MIR windows but require controlled temperature-humidity conditions [13, 19, 140]; and chalcogenides cover the 8–12 μm window where long-wave sensitivity is prioritized but thermomechanical limits must be managed [141]. Across these classes, the dominant limitation often shifts from intrinsic absorption to microstructure- and defect-induced scattering at elevated temperature, making processing quality and environmental stability as important as intrinsic optical constants.

### Covalent Semiconductors and Diamond

Covalent semiconductors and diamond sustain MIR transmission through strong directional bonding, suppressed multi-phonon overlap in the 3–5 μm window, and band structures that keep interband absorption away from the MIR range [142, 143]. Silicon combines an indirect band gap with high thermal conductivity, but long-wave transmission is limited by multi-phonon absorption and temperature-induced free carriers [144]. Germanium extends transmission toward longer wavelengths because of lower phonon energies, although its high refractive index and lower thermal conductivity require careful thermal management. GaAs provides useful 8–12 μm transmission with better mechanical robustness than many chalcogenides, but oxidation and arsenic volatility can introduce surface scattering at high temperature. Diamond is distinct because strong sp^3^ bonding, ultrahigh thermal conductivity, and low thermal expansion suppress thermal shock, optical figure distortion, and multi-phonon loss in the 3–5 μm window [145, 146]. Across this class, high purity, low dislocation density, near-theoretical densification, clean grain boundaries, and superpolished surfaces are required to prevent color centers, pore scattering, birefringence, and roughness-induced diffuse loss. Chemical compatibility defines the operating boundary: Si can form protective silica but may suffer silica volatilization at low pressure and high temperature; Ge oxidizes more readily; and diamond remains robust in many oxidizing conditions but may graphitize under catalytic environments [147]. When defect control, surface integrity, and atmosphere compatibility are aligned, covalent semiconductors and diamond preserve spectral fidelity and optical figure in demanding MIR windows.

**Fig. 13:**
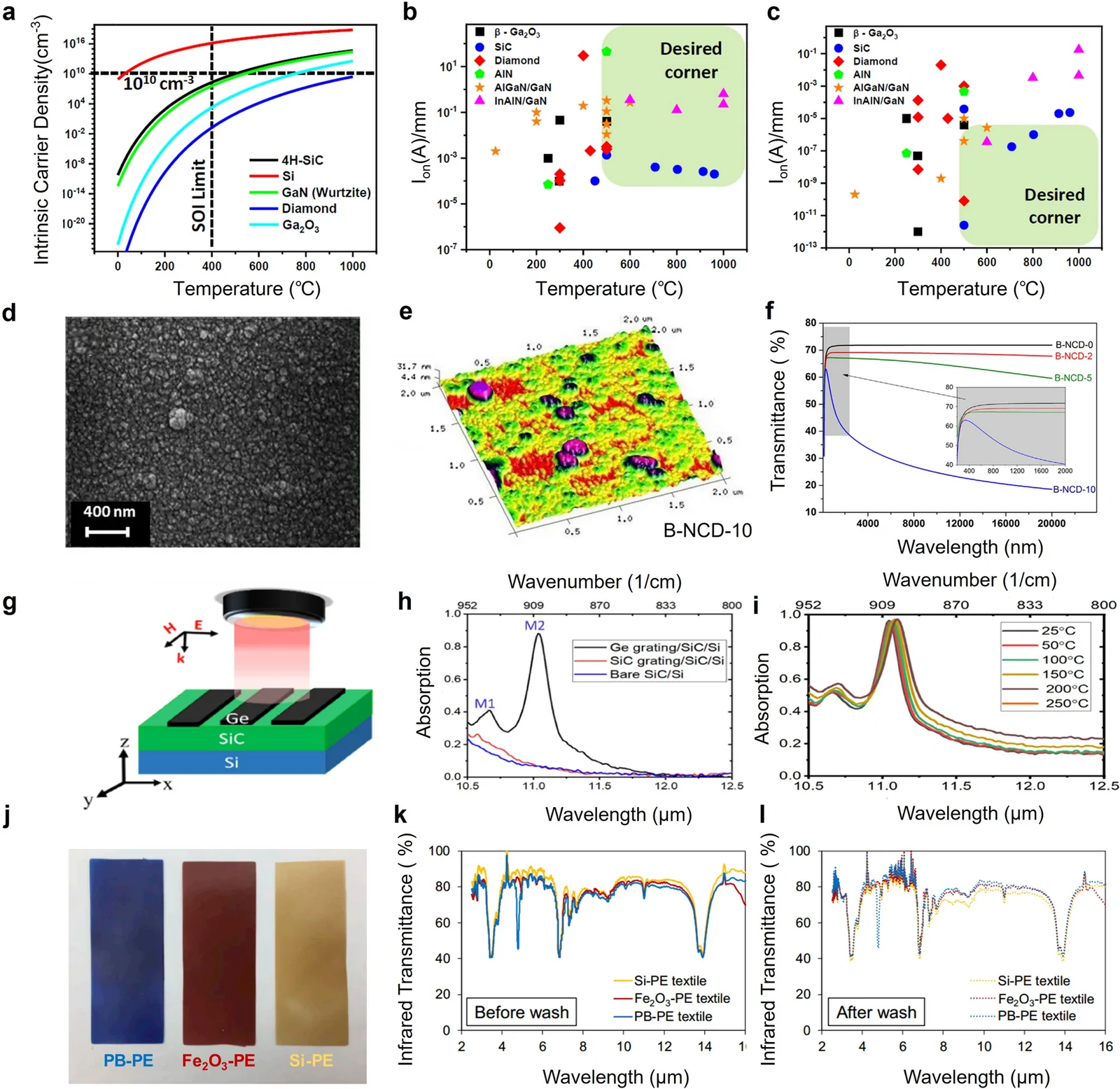
High-temperature-resistant MIR high-transmittance covalent semiconductors and diamond. **a** Temperature dependence of intrinsic carrier density in Si and wide-band gap semiconductors. **b** ON-state current densities of high-temperature logic devices. **c** OFF-state current densities of high-temperature logic devices. Reproduced with permission [57]. Copyright 2024, Springer Nature. **d** SEM morphology at 50k × magnification. **e** AFM topography of B-NCD films (2 × 2 μm^2^ region). **f** Broadband transmittance of diamond films Reproduced with permission [148]. Copyright 2015, Elsevier. **g** Ge-grating/SiC/Si multilayer structure. **h** Absorption resonances of photonic structure. **i** Temperature-evolving absorption spectra. Reproduced with permission [149]. Copyright 2025, AIP Publishing. **j** Structure of nanoparticle-polymer composite. **k** Transmittance spectra before cycling. **l** Transmittance spectra after cycling. Reproduced with permission [150]. Copyright 2019, Elsevier

Building on these mechanistic principles, experimental studies have explored how covalent semiconductors and diamond realize high-temperature MIR transparency. Jariwala et al. investigated wide-band gap semiconductors, showing that 4H-SiC maintains minimal band gap shrinkage above 850 °C (Fig. 13a), which suppresses free-carrier absorption and confirms SiC as a robust high-temperature platform (Fig. 13b, c) [57]. Sobaszek et al. deposited boron-doped nanocrystalline diamond (B-NCD) thin films, with SEM morphology at 50 k × magnification (Fig. 13d) and AFM topography over a 2 × 2 µm^2^ region revealing continuous (Fig. 13e), crack-free coatings. Despite nanoscale roughening from boron incorporation, the B-NCD films preserved mid-infrared transmittance from 200 nm to 20 µm, demonstrating that high-quality optical performance can be maintained alongside controlled doping (Fig. 13f) [148]. Extending photonic functionality, Iacopi et al. engineered Ge-grating/SiC/Si multilayers **(**Fig. 13g**)** supporting tunable MIR resonances (M1 at 10.6 µm, M2 at 11 µm). The devices exhibit spectral absorption peaks with directional and polarization-dependent characteristics over a range of incident angles, while maintaining thermally stable responses with only minor temperature-dependent variation from 25–250 °C **(**Fig. 13h, i**)** [149]. Complementarily, Cui et al. demonstrated colored mid-infrared-transmitting polymer composites by embedding nanoparticles such as Prussian blue, Fe_2_O_3_, or Si into polyethylene matrices (Fig. 13j). FTIR measurements of colored textiles before (Fig. 13k) and after (Fig. 13l) 120 washing cycles confirm that the composites maintain spectral stability, demonstrating robustness of the MIR-transmitting functionality under repeated laundering [150]. Overall, covalent semiconductors and diamond are best suited for 3–5 μm high-transmittance applications, while Ge and GaAs extend toward 8–12 μm; their use at elevated temperature requires control of oxidation, volatilization, and free-carrier generation.

### Oxide Transparent Ceramics

Oxide transparent ceramics sustain MIR transmission mainly in the 3–5 μm window by combining stable oxide lattices with microstructures engineered to suppress extrinsic scattering in oxidizing and thermal shock environments [151, 152]. Their intrinsic transmission limit is set by multi-phonon absorption, which increases sharply toward the long-wave band [153]; therefore, high transparency is achieved only when pores, grain-boundary phases, inclusions, and surface damage are reduced below this intrinsic loss baseline [154]. Crystal symmetry is also critical: cubic ceramics are optically isotropic and free of birefringence, whereas non-cubic systems introduce orientation-dependent grain-boundary scattering; thus, cubic spinel, ALON, garnet, and yttria generally transmit better than non-cubic ceramics at comparable density and purity [155, 156]. Sapphire’s uniaxial birefringence requires controlled orientation for imaging applications, while cubic spinel and ALON reduce polarization-related scattering but still require strict pore and inclusion control [157, 158]. Sintering aids, dopants, and surface treatments must also be optimized because excess additives, color centers, subsurface damage, or unstable coatings can introduce absorption, haze, or roughness during thermal cycling [159–161]. Chemically, alumina, spinel, ALON, YAG, and yttria resist oxidation and corrosive gases better than most non-oxide windows, although hydration or alkali attack can degrade MgO-rich compositions [162, 163]. When lattice symmetry, boundary chemistry, porosity, and surface preparation are jointly optimized, oxide transparent ceramics provide stable MIR transmission and optical figure retention for high-heat-flux apertures and viewports in oxidizing environments [164].

**Fig. 14:**
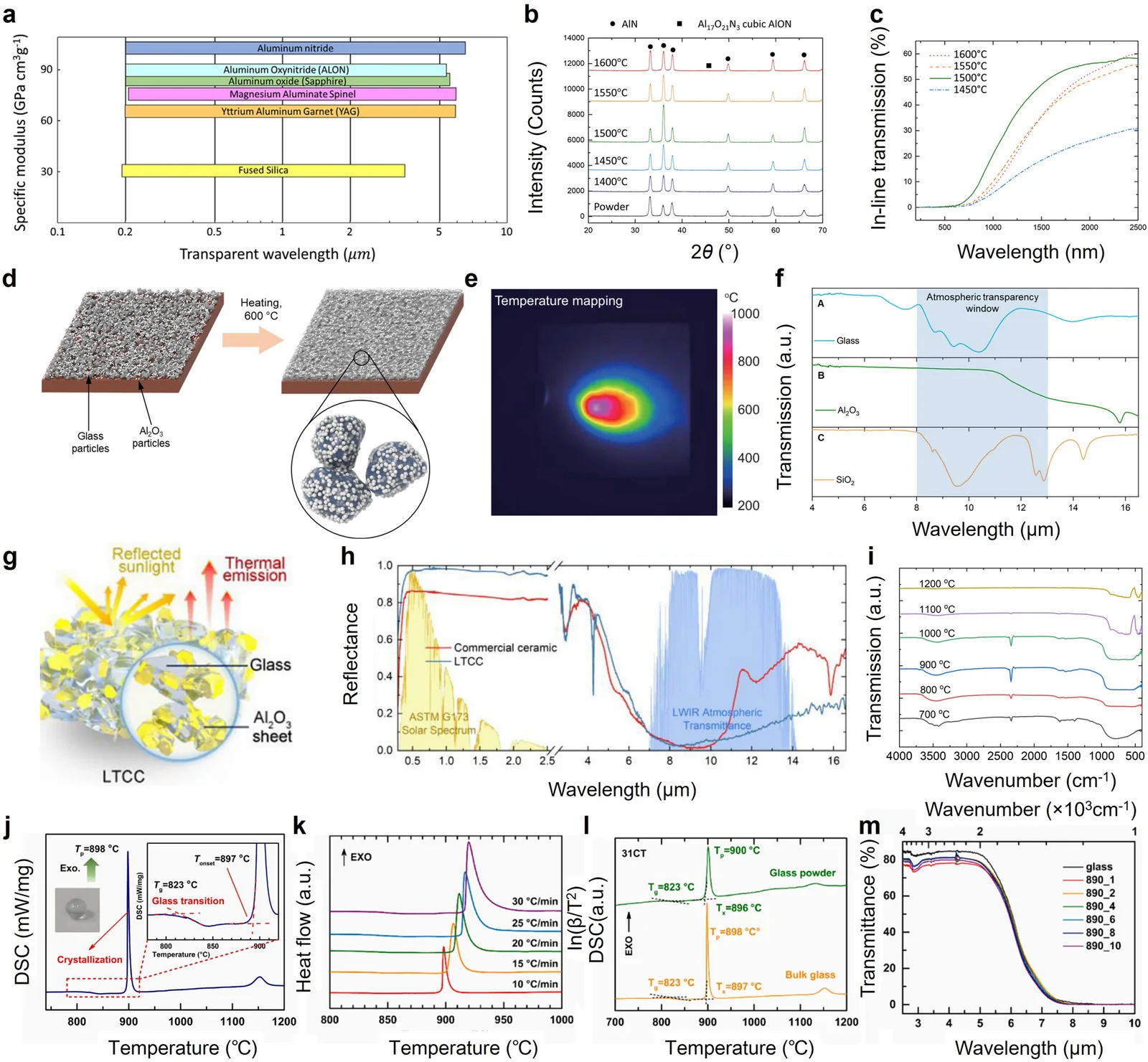
High-temperature-resistant MIR high-transmittance oxide transparent ceramics. **a** Transmission window and specific modulus of representative infrared-transparent materials. **b** XRD patterns of powders and bulk ceramics sintered. **c** Transmission of densified ceramics. Reproduced with permission [165]. Copyright 2022, Elsevier. **d** Schematic fabrication route for the radiative cooling glass coating. **e** Thermal shock exposure of the cooling glass coating under ~ 1000 °C flame. **f** FTIR spectra of oxide particles. Reproduced with permission [13]. Copyright 2023, AAAS. **g** Processing workflow and morphology of LTCC structures. **h** Spectral reflectance of ceramic laminates. **i** FTIR response of Al_2_O_3_-based sheets. Reproduced with permission [19]. Copyright 2025, John Wiley and Sons. **j** DSC analysis of glass precursor. **k** DSC profiles of glass samples under varying heating rates. **l** DSC comparison of powder and bulk samples. **m** Mid-infrared transmittance spectra. Reproduced with permission [166]. Copyright 2024, Elsevier

Building on these principles, recent studies have developed oxide ceramics with tailored densification, microstructure, and surface design for high-temperature MIR transparency. Garay et al. evaluated AlN and related window materials for thermal and optical applications **(**Fig. 14a**)**. Increasing sintering temperature from 1400 to 1600 °C under vacuum/controlled atmospheres reduced residual porosity and optimized grain-size distribution **(**Fig. 14b**)**. Directional MIR transmission measurements confirmed the resulting improvement in spectral transmittance, although the data were collected without an integrating sphere **(**Fig. 14c**)** [165]. Hu et al. fabricated a radiative cooling glass coating by heating low melting point glass microparticles to ~ 600 °C **(**Fig. 14d**)**. The resulting mesoporous network withstood short-duration flame exposure up to 1000 °C **(**Fig. 14e**)**. FTIR spectra confirmed multiple absorption/emission features in the 8–13 μm range together with stable solar reflectance and infrared emissivity under harsh conditions **(**Fig. 14f**)** [13]. Pang et al. developed SBS/glass/Al_2_O_3_ laminates, based on the morphology of LTCC structures (Fig. 14g), that transmit in the long-wave infrared (LWIR) atmospheric window while blocking solar radiation. Spectral reflectance measurements of the LTCC laminates, compared with commercial ceramics and solar/LWIR standards (Fig. 14h), confirm their selective optical performance, while structural integrity was maintained in MIL-96-derived Al_2_O_3_ sheets under thermal cycling (Fig. 14i) [19].

Extending functional design, Ruan et al. introduced CaO–Ta_2_O_5_–Al_2_O_3_ glass–ceramics (TGCs) embedding cubic α-CaTa₂O₆ nanocrystals. Differential scanning calorimetry (DSC) of 31CT glass spheres at 10 °C min^−1^ (Fig. 14j), DSC profiles under varying heating rates (Fig. 14k), and comparison between powdered and bulk 31CT glass confirmed the thermal behavior and crystallization characteristics (Fig. 14l). The TGCs were prepared via additive direct laser (ADL) processing with dual CO_2_ lasers, achieving uniform bulk crystallization and high transparency across the 0.5–5.5 µm spectral region (Fig. 14m) [166]. Overall, oxide transparent ceramics are the most reliable MIR window materials in oxidizing environments up to ~ 1000–1400 °C because of their chemical stability, hardness, and thermal shock tolerance, but their intrinsic phonon absorption limits long-wave 8–12 μm transmission relative to chalcogenides.

### Fluorides

Fluoride crystals such as CaF_2_, BaF_2_, MgF_2_, and LiF achieve extended MIR transmission because their highly ionic bonding and low phonon energies suppress multi-phonon absorption into the 8–12 μm region, while their relatively low refractive indices reduce Fresnel losses and simplify antireflection design [167, 168]. Their high-temperature use is limited less by intrinsic absorption than by environmental and mechanical stability. Hydration and hydroxyl incorporation can generate surface defects, micro-pitting, and scattering in humid flows, so reliable deployment requires dry atmospheres and surface chemistries that suppress hydroxyl uptake [169]. Thermal shock tolerance is also modest because thermal conductivity and fracture toughness are generally lower than those of oxide ceramics or covalent semiconductors, allowing steep gradients or particle impact to initiate microcracks and haze. Color centers formed by irradiation, stoichiometry drift, or halogen-vacancy aggregation introduce absorption tails, although controlled annealing can partially bleach these defects if grain growth and roughening are avoided [170, 171]. At elevated temperature, volatility and sublimation begin to matter for BaF₂ and LiF, while CaF_2_ offers better mass stability but still requires protection from alkali-assisted surface corrosion. Thus, fluorides are best used as LWIR-grade windows when atmospheres are dry, thermal gradients are limited, and defect chemistry is controlled to suppress hydration, color centers, and surface scattering [172, 173].

**Fig. 15:**
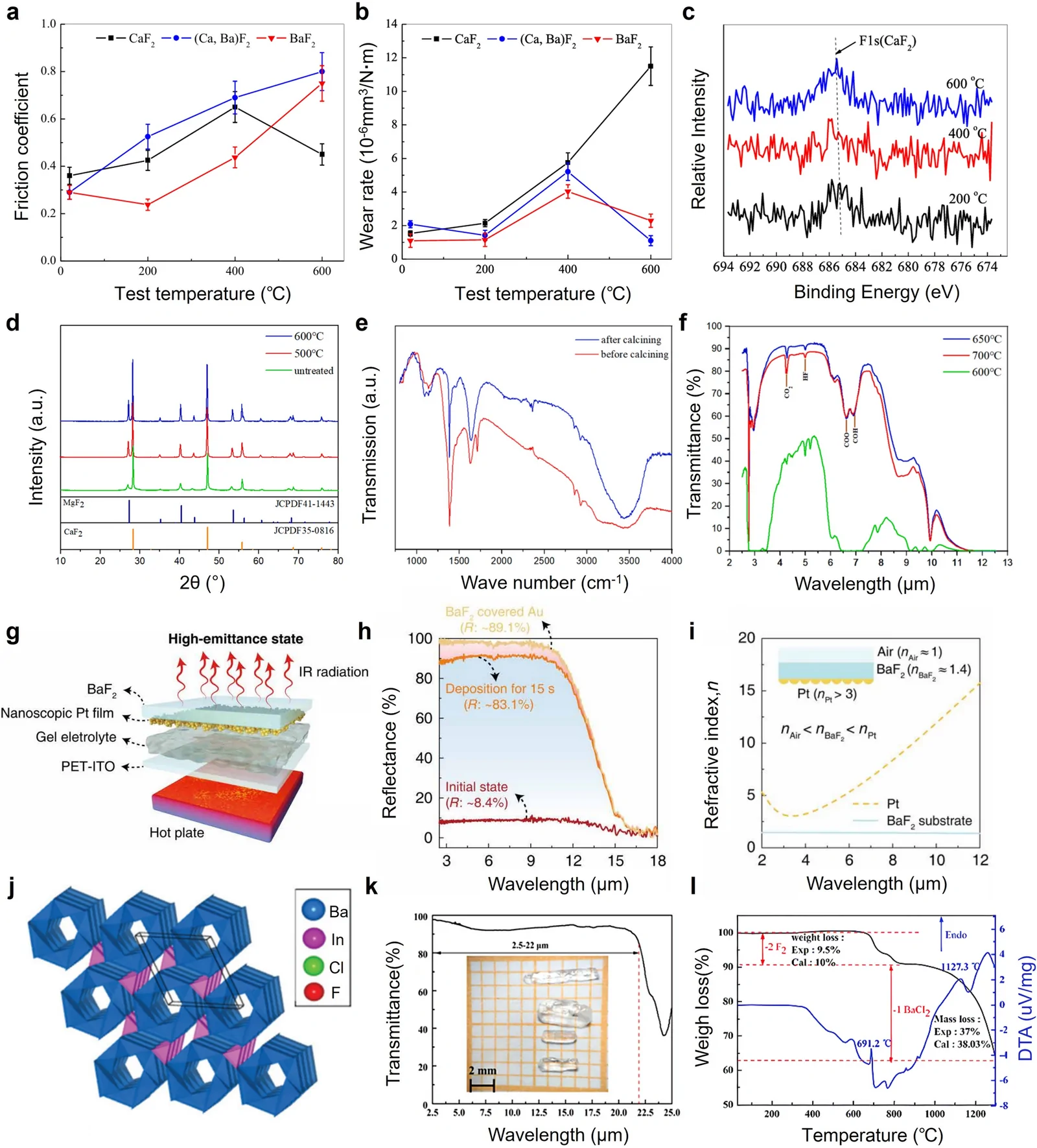
High-temperature-resistant MIR high-transmittance fluorides. **a** Temperature-dependent friction coefficients of the composites. **b** Wear-rate evolution of the composites with increasing temperature. **c** XPS F 1*s* spectra on worn (W_0.67_Al_0.33_)C_0.67_-Co/CaF_2_ surfaces at various temperatures. Reproduced with permission [172]. Copyright 2015, Elsevier. **d** XRD patterns of MgF_2_-CaF_2_ nanopowders calcined at different temperatures. **e** FTIR spectra before and after heat treatment. **f** Temperature-dependent transmittance spectra of HP-treated ceramics. Reproduced with permission [173]. Copyright 2022, Elsevier. **g** Structure of Pt/BaF₂-based device. **h** Infrared reflectance before and after modification. **i** Spectral refractive index of BaF_2_ and thin-film Pt. Reproduced with permission [174]. Copyright 2020, AAAS. **j** Crystal structure of In[Ba_3_Cl_3_F_6_]. **k** Infrared spectra of In[Ba_3_Cl_3_F_6_]. **l** TG–DTA analysis under inert atmosphere. Reproduced with permission [175]. Copyright 2020, Royal Society of Chemistry

Building on these intrinsic constraints, recent studies demonstrate how fluoride-based systems translate fundamental transparency into high-performance MIR optical components. Qiao et al. investigated the tribological behavior of (W_0.67_Al_0.33_)C_0.67_-Co/CaF_2_ composites, measuring temperature-dependent friction coefficients (Fig. 15a) and wear-rate evolution **(**Fig. 15b**)**. XPS F 1*s* spectra of worn surfaces at various temperatures revealed the evolution of surface chemistry, highlighting the critical role of fluoride stability in maintaining high-temperature tribological performance (Fig. 15c) [172]. Zhang et al. engineered MgF_2_/CaF_2_ nanocomposite ceramics (Fig. 15d), with XRD patterns characterizing nanopowders calcined at different temperatures and FTIR measurements assessing their response before and after 500 °C calcination (Fig. 15e). Temperature-dependent transmittance spectra of hot-pressed ceramics demonstrate that the optimized composites achieve > 90% transmittance in the 3–5 µm mid-infrared window, highlighting their potential for high-performance MIR optical applications (Fig. 15f) [173]. Extending functionality, Liu et al. fabricated adaptive thermal camouflage devices by integrating nanoscopic Pt films and Ag layers onto BaF_2_ substrates (Fig. 15g). Total infrared reflectance measurements of 3 nm Pt/BaF₂ before and after Ag electrodeposition (Fig. 15h), together with the spectral refractive index of BaF₂ and thin-film Pt, demonstrate that the multilayer architecture effectively modulates IR reflection and absorption across 3–14 µm (Fig. 15i) [174]. Yu et al. reported In[Ba_3_Cl_3_F_6_] (Fig. 15j), a metal-halide fluoride with a broad transparency window from 0.366 to 22 μm and confirmed thermal stability under prolonged heating (Fig. 15k). This stability is supported by TG–DTA analysis conducted under a N₂ atmosphere, demonstrating the material’s suitability for high-temperature infrared optical applications (Fig. 15l) [175]. Overall, fluoride materials provide extended MIR-to-LWIR transmission owing to low phonon energies, but their limited thermal shock, humidity, and chemical stability make them more suitable for moderate temperature, dry, or protected environments than for extreme oxidizing service.

### Chalcogenides

Chalcogenide materials, including II-VI semiconductors such as ZnSe and ZnS and multicomponent S-, Se-, or Te-based glasses [176, 177], enable LWIR transmission by combining low vibrational frequencies with high static polarizability, which shifts multi-phonon absorption to longer wavelengths and opens broad 8–12 μm windows [178, 179]. Crystalline ZnSe and multispectral ZnS provide low intrinsic absorption when stoichiometry, inclusions, and pores are controlled [180], while chalcogenide glasses offer broader forming flexibility for complex optics. Their main limitations are thermomechanical and environmental: lower hardness, fracture toughness, and thermal conductivity increase sensitivity to erosion [181], impact, and thermal shock, while structural relaxation, sub-Tg creep, photoinduced defects, moisture, halogens, and surface oxides can distort the optical figure or introduce absorption/scattering during cycling [182]. Surface quality and coating compatibility are therefore decisive, because roughness approaching MIR wavelengths, incompatible oxide coatings, or moisture-driven network modification can rapidly degrade LWIR transmission [141, 183]. Under conditions where humidity is controlled, thermal gradients are limited, and surface chemistry is stabilized, chalcogenides provide LWIR-capable windows and domes with wide passbands and manageable dispersion, occupying a space between the high mechanical robustness of oxide ceramics and the extreme long-wave transparency of fluorides.

**Fig. 16:**
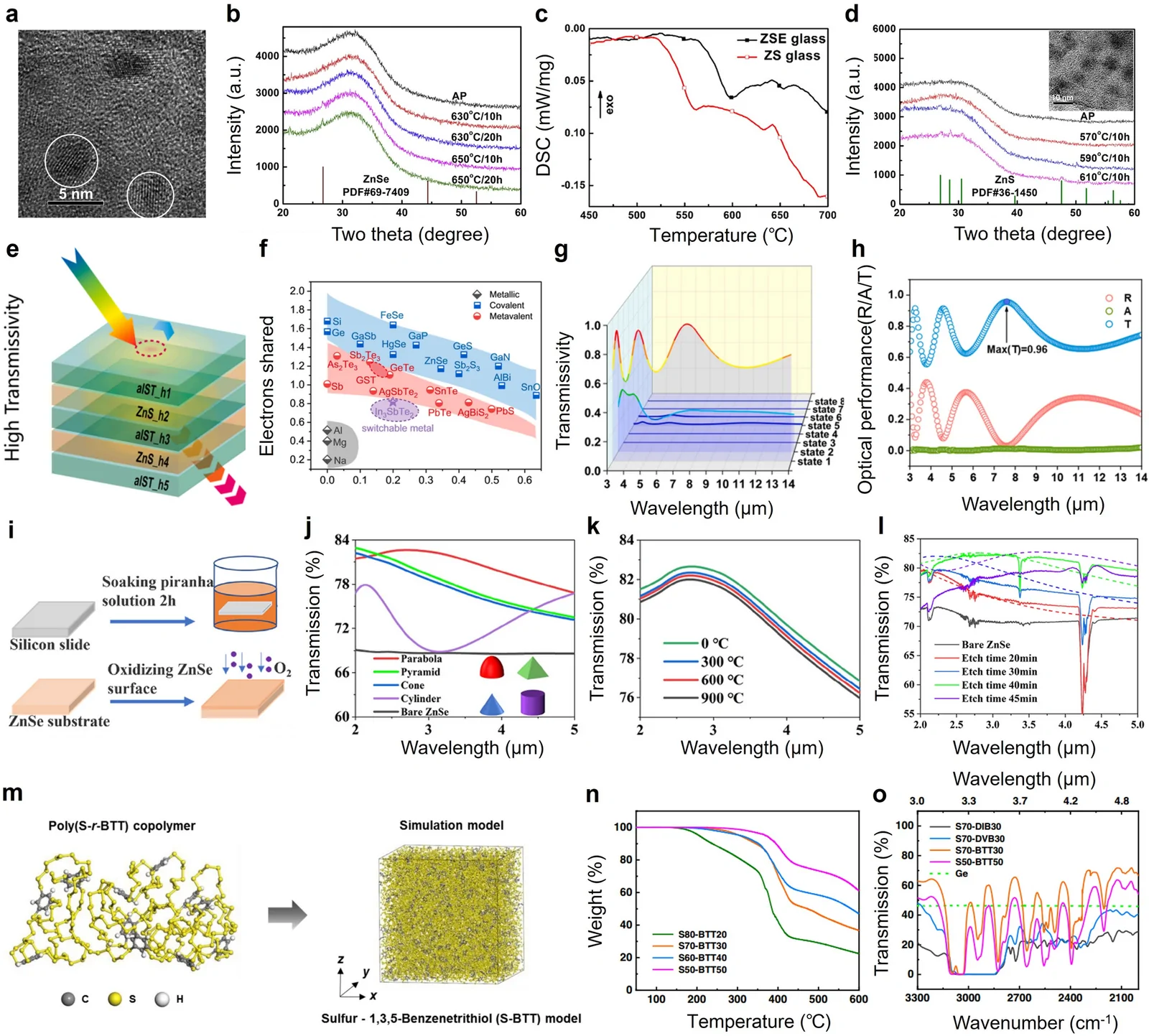
High-temperature-resistant MIR high-transmittance chalcogenides. **a** HR-TEM micrograph of the ZSE specimen annealed. **b** XRD patterns of ZSE before and after thermal treatment. **c** DSC profiles of as-prepared ZSE and ZS glasses. **d** XRD patterns and HR-TEM inset of ZS samples following heat treatment. Reproduced with permission [183]. Copyright 2015, Elsevier. **e** Schematic representation of infrared transmissivity. **f** Bonding characteristics in crystalline and metallic systems. **g** Mode-resolved transmissivity. **h** Optical characteristics under high-transmissivity operation. Reproduced with permission [184]. Copyright 2025, Elsevier. **i** Schematic of moth-eye nanostructures on a ZnSe substrate. **j** Transmittance spectra of MENS structures. **k** Temperature-induced variations in transmittance for MENS. **l** FTIR transmittance of MENS fabricated with different etching durations. Reproduced with permission [141]. Copyright 2023 Elsevier. **m** Chemical structure of the poly(S-rBTT) copolymer and its simulation model. **n** TGA curves and DSC-derived glass transition temperatures. **o** MWIR transmission spectra of polymer windows. Reproduced with permission [185]. Copyright 2023, Springer Nature

Building on these mechanistic considerations, recent studies have engineered chalcogenide systems for robust LWIR performance under high-temperature conditions. Liu et al. investigated ZnSe-based (ZSE) nanocrystals embedded in glass (Fig. 16a), with high-resolution TEM revealing nanocrystal formation after heat treatment at 650 °C for 10h (Fig. 16b) [183]. Differential scanning calorimetry revealed a glass transition temperature of ~ 574 °C for ZSE glass, whereas ZS glass exhibited an exothermic peak at 642 °C. Thermal treatment of ZS glass at 590–610 °C induced diffraction peaks consistent with hexagonal wurtzite ZnS nanocrystals (Fig. 16c), accompanied by spectral absorption and photoluminescence features as confirmed by XRD patterns and HR-TEM imaging of the heat-treated samples (Fig. 16d). Zhu et al. extended this approach through multilayer thin-film architectures using phase-change material (PCM) In_3_SbTe_2_ (Fig. 16e) [184], comparing the bonding characteristics in crystalline porous solids versus metals (Fig. 16f), highlighting how structural control influences optical behavior. Mode-resolved transmissivity measurements in the 3–14 µm infrared range reveal eight distinct structural modes (Fig. 16g), achieving transmissivity up to 0.96, demonstrating that layer engineering and phase tuning are critical for optimizing long-wave infrared (LWIR) transmission (Fig. 16h). Deng et al. enhanced transmission via surface nanostructuring, fabricating moth-eye nanostructures (MENS) on ZnSe substrates (Fig. 16i). MENS with 800 nm height exhibited significant spectral transmittance enhancement in the 2–5 μm range (Fig. 16j), with directional (angle-dependent) optical response over a wide incidence range, and minimal performance degradation up to 900 °C, indicating stable behavior under thermal variation **(**Fig. 16k**)**. Varying etching times further enabled fine control of the optical response **(**Fig. 16l**)** [141].

Complementing crystalline and thin-film approaches, polymer-based chalcogenide windows offer a versatile route to high-temperature LWIR optics. You et al. employed inverse vulcanization of 1,3,5-benzenetrithiol (BTT) to form poly(S-r-BTT) copolymers (Fig. 16m) [185]. Increasing BTT content enhanced thermal stability, char yield, and decomposition temperature (Fig. 16n), with the S70-BTT30 composition achieving over 60% transmission in the mid-wave infrared (MWIR) (Fig. 16o). Overall, chalcogenides are attractive for 8–12 μm windows, filters, and thermally adaptive optics because of their low phonon energy, phase/network tunability, and surface-structured antireflection designs. However, their soft bonding, low thermal conductivity, and sensitivity to oxidation, moisture, halogens, and sub-T_g_ structural relaxation make them more suitable for moderate temperature or protected LWIR service, whereas oxide ceramics and covalent semiconductors remain more reliable under oxidizing, mechanically demanding, or high-flux conditions.

## Structural Determinants of Mid-Infrared Performance in High-Temperature-Resistant Materials

**Fig. 17:**
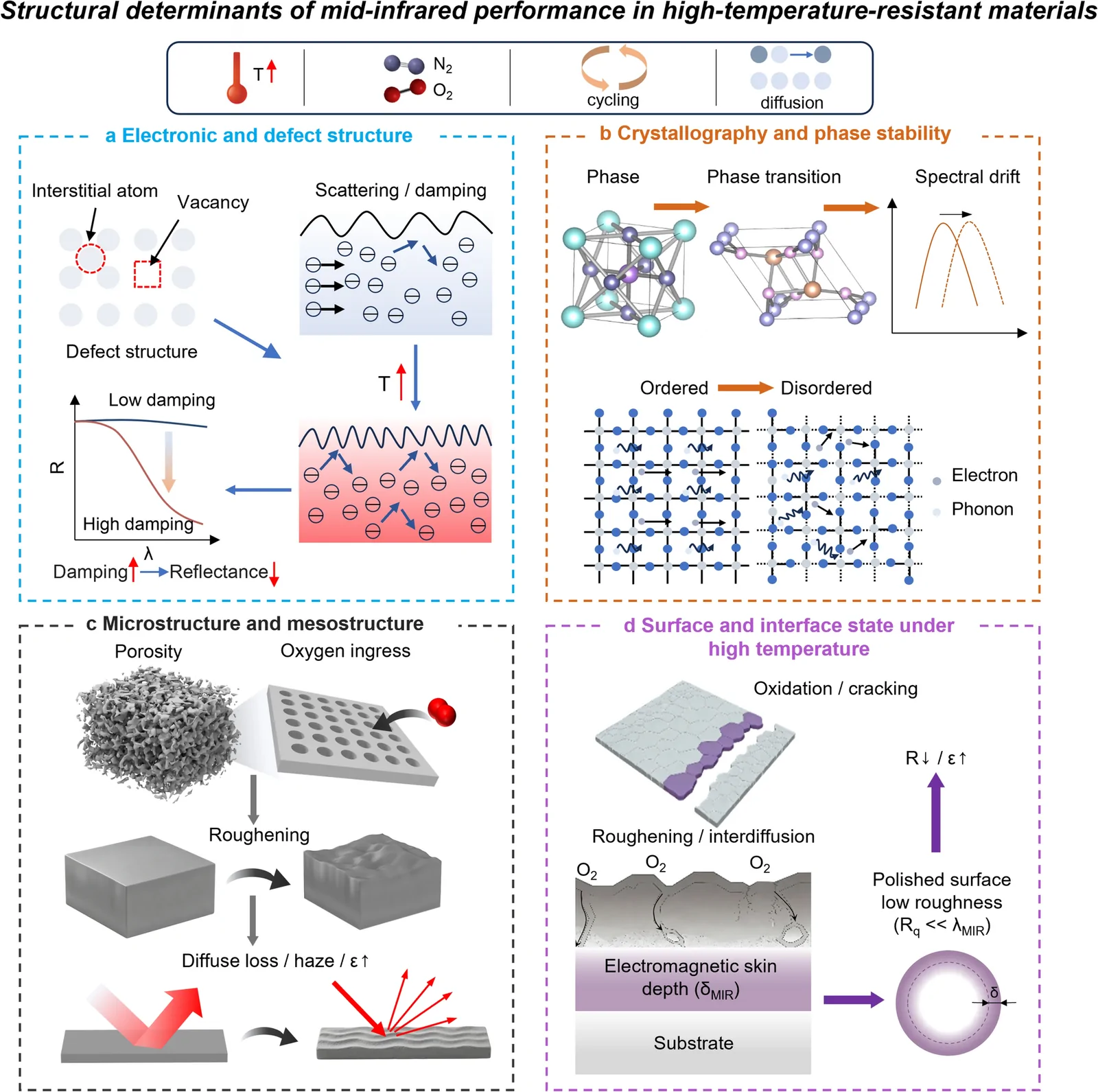
Structural determinants of mid-infrared performance in high-temperature-resistant materials. **a** Defects increase carrier scattering and damping, reducing reflectance. **b** Phase transitions and disorder induce spectral drift. **c** Porosity and roughening enhance scattering, leading to diffuse loss (haze) and increased emissivity. **d** Surface/interface evolution within the electromagnetic skin depth (δ_MIR_) governs reflectance and emissivity under high temperature

Mid-infrared performance at high temperature is governed not only by the nominal chemistry of a material but by how its internal structure evolves under load. The decisive variables concentrate into four hierarchies that act from electronic scale to surface scale and couple strongly to optical response (Fig. 17). Carrier populations, defect equilibria, and non-stoichiometry set the magnitude and damping of free-carrier and lattice contributions and therefore the baseline for reflectance, emittance, and transmittance [37, 49, 126]. Crystal symmetry, phase stability, and phonon anisotropy control the placement and temperature drift of interband and Reststrahlen features and determine whether selective or broadband behavior is feasible. Microstructural attributes such as densification, grain size, boundary character, porosity, and preferred orientation govern extrinsic scattering and the transport of species that drive aging [186]. Surface and interface states complete the picture because oxide scale growth, roughness evolution, and near-surface conduction within the electromagnetic skin depth often dominate the observed spectrum in service. The following subsections resolve these structure–property linkages and identify the mechanisms that preserve or degrade targeted mid-infrared functions as temperature and atmosphere vary [187].

### Electronic and Defect Structure

Electronic and defect structure defines the starting point for MIR response by setting carrier density, carrier mobility, and the population of scattering or absorption centers. Figure 17a summarizes how carrier density, damping, non-stoichiometry, and defect-induced absorption centers control MIR optical drift. In high-reflectance systems such as metals, nitrides, and conductive oxides, strong free-carrier screening requires high carrier density with limited damping; however, phonons, impurities, and point defects increasingly scatter carriers as temperature rises. The origin of defects is temperature-dependent: extrinsic dopants and processing-induced non-stoichiometry usually dominate at lower temperatures, whereas intrinsic thermal defects become increasingly important above a characteristic temperature and can strongly modify carrier concentration and mobility. Non-stoichiometry therefore has a dual effect [37, 79]. Nitrogen vacancies in transition metal nitrides and oxygen vacancies in conductive oxides can donate carriers and improve reflectance within a limited range, but excessive vacancy content increases disorder, raises damping, shifts the effective plasma edge, and may promote defect clustering [81, 82]. In conductive oxides, oxygen chemical potential reconfigures vacancy/interstitial populations and dopant charge states, while in metallic oxides even small cation or oxygen deviations can localize carriers and increase MIR loss [78, 79]. In absorptive carbides, carbon deficiency can enhance broadband loss but may also embrittle the lattice and accelerate oxidation [104]. In transmissive materials, unintentional donors, color centers, halogen vacancies, impurity complexes, Frenkel pairs, and dislocation-related traps introduce free-carrier absorption, MIR tails, or haze. Stable MIR performance therefore requires dopants with low diffusion and compensation tendency, host chemistries that limit subsurface vacancy injection, and post-annealing under controlled oxygen or nitrogen activity to lock the defect population into a functional window [79, 174].

### Crystallography and Phase Stability

Crystallography and phase stability govern MIR performance by defining symmetry-allowed electronic/vibrational states, anisotropic optical constants, and thermal transformation pathways. Crystal symmetry sets selection rules for interband transitions and phonon activity, determining whether a material exhibits broad metal-like response or sharp Reststrahlen features aligned with the 3–5 or 8–12 μm windows. Anisotropy further shapes directional response [123, 127]. Figure 17b illustrates how symmetry, phonon activity, phase transformation, and thermal expansion mismatch drive MIR spectral drift. Uniaxial systems such as sapphire or layered carbides exhibit different ordinary/extraordinary indices and unequal phonon lifetimes [188], whereas cubic nitrides, carbides, spinels, and garnets are more isotropic if the phase remains single and coherent [183]. Phase transitions, order-disorder transformations, oxygen/nitrogen uptake, cation off-stoichiometry, or grain-boundary complexion changes can shift plasma edges [189], broaden phonon bands, or convert metallic states into more lossy correlated/insulating phases [190, 191]. Thermal expansion anisotropy can also generate microcracks and birefringence drift; for example, sapphire shows anisotropic expansion of approximately α⊥c ≈ 8.5 × 10^–6^ K^−1^ and α//c ≈ 5.0 × 10^–6^ K^−1^, whereas AlN combines high thermal conductivity (~ 320 W m^−1^ K^−1^) with higher thermal shock resistance than alumina. The most durable responses arise when the operating window lies within a single-phase stability field with slow diffusion, limited volatile species, and phonon spectra that evolve smoothly rather than undergoing abrupt reconstruction [50, 173, 192].

### Microstructure and Mesostructure

Microstructure and mesostructure translate intrinsic optical constants into realized MIR performance by controlling scattering, diffusion, and mechanically driven aging. Figure 17c links pores, grain boundaries, grain-size evolution, and mesoscale defects to scattering, haze, and diffuse MIR loss [193]. Densification establishes the baseline: residual pores from hundreds of nanometers to several micrometers scatter strongly across the 3–12 μm MIR range and convert specular transmission or reflection into diffuse loss [194, 195]. Grain size and boundary chemistry then determine both optical loss and thermal stability [196]. Fine grains can suppress crack propagation and faceting, but excessive boundary density increases carrier/phonon damping and may host glassy or impurity-rich films; coarse grains reduce boundary scattering but can promote thermal grooving, roughness growth, and skin-depth-scale surface evolution. Boundary segregation of light elements, halogens, or dopants modifies local polarizability and can introduce color centers or low-melting films during cycling [165, 197]. Texture provides another lever: basal-plane alignment in graphitic or layered systems improves in-plane thermal transport [198], whereas cubic, weakly anisotropic textures in transparent oxides reduce polarization artifacts and stress birefringence [12, 116]. As semi-quantitative guidance, porous transparent ceramics follow an approximate scattering-loss trend in which transmittance decreases with porosity, pore size, and optical path length; Rayleigh-type scattering dominates for subwavelength pores, while Mie scattering becomes severe when pores or grains approach the operating wavelength [165, 166]. For conductive materials, grain-boundary scattering can be treated as an additional damping contribution, so increasing boundary density or decreasing grain size raises effective damping and thereby increases MIR emissivity. Microstructural design therefore converges on three rules: minimize connected porosity and impurity-rich boundaries, balance grain size against roughening kinetics, and preserve a mechanically robust surface zone whose evolution remains below the relevant optical length scale [199].

### Surface and Interface State under High Temperature

Surface and interface states often dominate high-temperature MIR behavior because optical fields probe shallow near-surface regions that are also most vulnerable to oxidation, roughening, interdiffusion, and stress damage. Figure 17d summarizes how oxide scales, roughness, interdiffusion, reaction layers, and skin-depth effects govern near-surface MIR degradation [200]. Oxide scale growth is a primary limitation. On metallic reflectors, nitrides, and carbides, oxygen ingress forms surface layers with dielectric properties different from the substrate; when scale thickness approaches the optical penetration depth or skin depth, specular reflectance can decrease and hemispherical emissivity can increase even if the bulk remains intact [130, 201]. This thickness criterion must be considered together with refractive index contrast: when the oxide and substrate have similar refractive indices, reflectance degradation can be partly mitigated even if the oxide exceeds the skin-depth scale, whereas strong mismatch accelerates optical loss even for thinner layers. Volatile oxides in Mo- or W-based systems further cause recession, voiding, and roughening, while passivating Al_2_O_3_ or SiO_2_ scales slow reaction but may add absorption after densification or crystallization. Surface roughness is the second major pathway [60]. Thermal grooving, creep faceting, erosion, and subsurface polishing damage convert specular reflection or transmission into diffuse scattering; roughness becomes optically significant when its characteristic scale approaches a non-negligible fraction of the operating wavelength, especially in the 3–12 μm MIR range [202]. Interfaces add instability through interdiffusion, reaction layers, residual stress, cracking, crazing, and delamination [203]. In transmissive materials, hydroxylation, adsorbates, alkali exposure, or moisture-induced restructuring introduce absorption tails and haze, particularly in fluorides, chalcogenides, and oxide ceramics [172, 183].

To provide semi-quantitative guidance, porous transparent ceramics can be described by scattering-induced transmittance loss $T \approx T_{0} \exp ( - f_{0} \mu_{s} t)$ [38], where ${\mu}_{s}$ increases with porosity, pore radius, and refractive index contrast,${\mu}_{s}\propto P{r}^{3}\mathrm{/}{\lambda }^{4}$, indicating that even low porosity can reduce in-line transmittance when pore radius r, porosityP, or optical path length t increases [204]. When pores or grains become wavelength-comparable ($r\sim \lambda$), Mie-type scattering dominates, leading to rapid loss of transmittance and specular reflectance [205]. For conductive materials, grain-boundary scattering can be treated as an additional damping contribution,${\gamma}_{\mathrm{eff}}\mathrm{=}{\gamma}_{0}\mathrm{+}{\gamma}_{\mathrm{gb}}$, with ${\gamma}_{\mathrm{gb}}\propto {v}_{F}\mathrm{/}d$ or increasing with grain-boundary density (d=${d}_{\mathrm{g}\mathrm{b}}$); the resulting increase in ${\gamma}_{\mathrm{eff}}$ enhances free-carrier loss and raises MIR emissivity [206]. Similarly, RMS roughness becomes optically significant when it reaches a non-negligible fraction of the operating wavelength, while oxide/reaction layers become critical when their thickness approaches the optical penetration depth or skin depth. These criteria indicate that high-temperature MIR stability requires low porosity, subwavelength roughness, minimized grain-boundary damping, and reaction layers thinner than the optical penetration depth unless refractive index matching reduces optical contrast [59, 81].

## Consolidated Dataset of High-Temperature-Resistant Mid-Infrared Materials

**Fig. 18:**
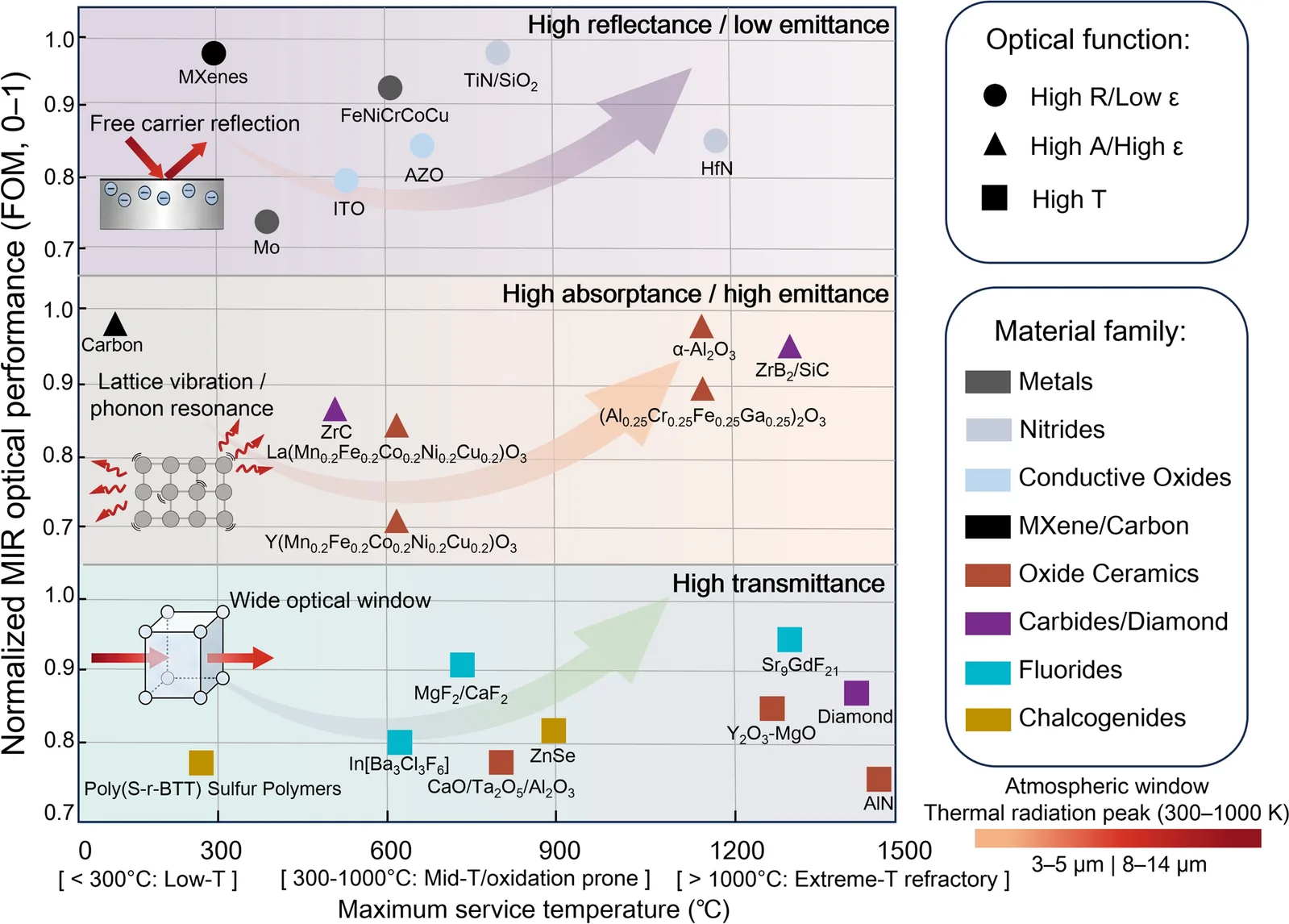
Visual classification map of representative MIR material systems discussed within the functional framework proposed in Fig. 1, including high-reflectance, high-absorptance/emittance, and high-transmittance categories

To improve the structural consistency of the review, Fig. 18 and Table 1 are presented as complementary extensions of the conceptual framework established in Fig. 1. Specifically, Fig. 18 provides a visual classification map of representative MIR material systems, while Table 1 summarizes the corresponding optical performance, temperature tolerance, and structural characteristics across different functional categories. In the reflectance class, metals and alloys set the upper bound when surfaces remain smooth and unoxidized: a Cu-Ni alloy entry reports *R*_2-15_ ≈ 0.9847 [61], BaF_2_/Pt/Ag reaches *R*_3-14_ ≈ 0.95 [174], and SiC/Ag maintains *R*_3-25_ ≥ 0.90 [101]; drift appears once oxide scales thicken or roughness grows. Conductive oxides occupy an intermediate tier but with superior tolerance to oxygen, consistent with carrier reservoirs stabilized by an already oxidized lattice; ITO- and AZO-based stacks retain high *R* where noble films degrade. Transition metal nitrides approach metallic performance with refractory bonding; representative TiN/SiO_2_ records very low effective emissivity (*ε* ≈ 0.03) with stability reported to about 727 °C [82], while HfN/ZrN sustain high *R* provided columnar pathways for oxidation are suppressed. MXene films show competitive room temperature optics (*ε* ≈ 0.06–0.17; one dense film lists *R* ≈0.938) yet usable windows center near 300–400 °C [11, 52], reflecting termination chemistry and interlayer diffusion. On the absorptance side, carbon architectures and carbide ceramics deliver near-gray high emittance that strengthens with temperature: carbon entries list *A*_8-12_ ≈ 0.97–0.997 but only 60–100 °C tolerance in air without protection [207], whereas SiC shows *A*_10.5–12_ ≈ 0.957-0.97 with operation well above 1,000 °C [101]; ZrB_2_/SiC is reported to ~ 1,300 °C [208]. Oxide ceramics provide selective phonon–polariton emission with *ε* ≈ 0.80–0.95 and atmosphere windows extending to 1,500 °C; high-entropy and complex perovskites cluster around *ε* ≈ 0.72–0.89 at 600–1,100 °C [126, 127], suggesting cation disorder can broaden or place emissive bands without phase collapse. Transmission trends mirror intrinsic phonon limits plus scattering control. Diamond anchors high-heat-flux apertures with *T*_3-5_ > 0.85; Si and Ge stacks show *T* ≈ 0.75–0.90 with durability set by oxidation management [209, 210]. Oxide transparent ceramics achieve robust mid-wave clarity when porosity and glassy boundaries are suppressed, with Y_2_O_3_-MgO and yttria near *T*_3-5_ ≈ 0.84 and service to ~ 1,300 °C; alumina, spinel, ALON, and YAG extend to ~ 1,500 °C [49, 165]. Fluorides and chalcogenides extend into the LWIR, e.g., MgF_2_/CaF_2_ with *T* ≈ 0.90 near 650 °C [173], ZnSe with *T* ≈ 0.826 near 900 °C, and an IST/ZnS record at *T* ≈ 0.96 under controlled humidity [141, 184]. Across categories, the most stable entries share three structural traits evident in the table: near-theoretical densification that eliminates Mie-scale pores, clean grain boundaries resistant to grooving and interdiffusion, and surfaces kept smoother than the optical skin depth through superpolishing or compatible texturing. These correlations indicate that retention of mid-infrared figures of merit is governed less by room temperature dielectric constants and more by the kinetics of oxidation, vacancy drift, and roughness evolution that modulate near-surface loss at high temperature.

**Table 1:** Consolidated dataset of representative MIR materials discussed in this review, organized according to the functional classification framework introduced in Fig. 1 and corresponding to the material categories summarized in Fig. 18

Structure/material	Structure design	*R*_high_/*ε*_low_	*A*_high_/*ε*_high_	*T*_high_	Temperature stability (°C)	Ref
Metals and Alloys	Mo	*R*_3-8_ > 0.70			400	[60]
	FeNiCrCoCu	*R*_3-14_ = 0.90			600	[37]
	PE/Fe_2_O_3_/Si/ZnO/Al	*R*_7-14_ = 0.90				[211]
	BaF_2_/Pt/Ag	*R*_3-14_ = 0.95				[174]
	SiC/Ag	*R*_3-25_ = 0.90				[101]
	Pt/graphene/Au/PE/Cu	*ε*_7.5–13_ = 0.07				[212]
	WKF/Cu-Ni/rGO/PDMS	*R*_2-15_ = 0.9847				[61]
Conductive Oxides	BaF_2_/AZO NCs/ITO	*ε*_7.5–13_ = 0.41				[79]
	Mg_x_Ni/Pd/PEI/H_x_WO_3_/ITO	*ε*_7.5–14_ = 0.43				[78]
	ITO/Ag/ITO	*R*_3-25_ > 0.09				[77]
Transition Metal Nitrides	HfN/Ag	*R*_3-12_ = 0.95				[84]
	SiO_2_/TiN NPs/Ag	*ε*_0.3–20_ = 0.02			100	[83]
	TiN/SiO_2_	*ε*_8-13_ = 0.03			727	[82]
MXene Family	MXene/CNT	*ε*_2.5–15_ = 0.09			300	[52]
	Ti_3_C_2_T_x_	*ε*_3-25_ = 0.06				[99]
	Ti_3_C_2_T_x_ Mxene	*ε*_2.5–16.7_ = 0.10–0.17			400	[11]
	Ti_3_C₍_2-_ᵧ₎NᵧT_x_ Mxene	*R*_1-25_ = 0.938			300	[213]
Carbon-Based Materials	Carbon		*A*_8-14_ = 0.97–0.997		60–100	[207]
	Al_2_O_3_/rGO		*ε*_8-20_ = 0.83			[119]
	Graphene/BaO/Ag		*A*_2.8–4.4_ = 0.96			[118]
	Ni/graphene/Al_2_O_3_/Au/SiO_2_		*A*_8−12_ = 0.70			[116]
High-entropy Oxide Ceramics	La(Mn_0.2_Fe_0.2_Co_0.2_Ni_0.2_Cu_0.2_)O_3_		*ε*_3-8_ = 0.855		600	[214]
	Y(Mn_0.2_Fe_0.2_Co_0.2_Ni_0.2_Cu_0.2_)O_3_		*ε*_3-8_ = 0.718		600	[214]
	La_0.16_(CaCuCoNiMg)_0.84_Mn_2_O_4_		*ε*_0.78–16_ = 0.95		800–1100	[126]
	(Ga_0.2_Ni_0.2_Co_0.2_Mn_0.2_Zn_0.2_)_3_O_4_		*ε*_2.5–16_ = 0.891		1300	[127]
	(Al_0.25_Cr_0.25_Fe_0.25_Ga_0.25_)_2_O_3_		*ε*_2.5–14_ = 0.90		1000	[50]
	(La_0.2_Y_0.2_Nd_0.2_Gd_0.2_Sr_0.2_)CrO_3_/SiO_2_		*ε*_0.7–15_ = 0.971		1573 K	[2]
Oxide Ceramics	α-Al_2_O_3_		*ε*_8-13_ = 0.95		1000	[13]
	Al_2_O_3_/Fe/Co/Mn/Al		*A*_1.6–3.2_ = 0.8		1500	[215]
	Al/Al_2_O_3_/CuO/MgO		*ε*_2.5–20_ = 0.94–0.95		400	[216]
	Gd_3_TaO_7_/GdFeO_3_		*ε*_2.5–14_ = 0.9		1500	[217]
	Ge/SiO_2_/Ge/SiO_2_		*ε*_5-8_ = 0.75		500	[149]
Carbide Ceramics	Ge Grating/3C-SiC/Si Metasurface		*ε*_10.5–12.5_ = 0.95		400	[101]
	SiC		*A*_10.6–12.7_ = 0.97			[101]
	SiC/Ag		*A*_10.5–12.5_ > 0.80			[218]
	ZrB_2_/SiC		*ε*_6-16_ = 0.70–1.00		1300	[208]
	ZrC		*A*_2.5–25_ = 0.86		481.6	[104]
Covalent Semiconductors and Diamond	Diamond			*T*_1-5_ = 0.85		[209]
	Ti/Ge/Ti			*T*_5-8_ > 0.9		[210]
	PB/Fe_2_O_3_/Si			*T*_7-14_ = 0.80		[150]
Oxide Transparent Ceramics	AlN			*T*_4.3_ = 0.73	1600	[165]
	CaO/Ta_2_O_5_/Al_2_O_3_			*T*_0.5–5.5_ = 0.80	890	[166]
	Y_2_O_3_-MgO			*T*_3-5_ = 0.84	1300	[49]
Fluorides	Sr_9_GdF_21_			*T*_3-12_ > 0.92	1300	[219]
	In[Ba_3_Cl_3_F_6_]			*T*_2.5–22_ > 0.80	650	[175]
	BaCuSF/SiO_x_			*T*_0.5–30_ > 0.50	100	[220]
	MgF_2_/CaF_2_			*T*_3-5_ = 0.90	650	[173]
Chalcogenides	IST/ZnS			*T*_3-14_ = 0.96		[184]
	ZnSe			*T*_2-5_ = 0.826	900	[141]
	Poly(S-r-BTT) Sulfur Polymers			*T*_3-14_ > 0.60	200	[185]

To improve cross-material comparability beyond optical figures of merit, Table 2 summarizes the experimental conditions and stability-related parameters of representative MIR materials, including atmosphere, measurement type, angular condition, exposure time, degradation behavior, and characterization methods [37, 60, 83]. The comparison shows that reporting remains uneven: atmospheres and exposure durations are often unspecified, degradation is frequently described qualitatively rather than kinetically, and angular or hemispherical measurement geometries are rarely clarified [77, 79]. These gaps indicate that discrepancies in reported high-temperature MIR performance arise not only from intrinsic material differences, but also from inconsistent testing and reporting practices; therefore, Table 2 provides a condition-aware reference for evaluating environmental stability, oxidation resistance, and microstructural evolution under realistic service conditions.

**Table 2:** Summary of experimental conditions and stability metrics of representative MIR materials

Material	Atmosphere	Measurement type	Angle	Exposure time	Degradation	Characterization	Ref
Mo-AlN-Mo		Spectral *R*			Resonance stability under heating	FESEM, XPS, FTIR	[60]
FeNiCrCoCu/Al_2_O_3_	Air	Spectral *R*; *ε*		20h	Oxidation at 700 °C	XRD, AFM, SEM, FTIR	[37]
ITO/Ag/ITO		Spectral *T*; Spectral *R*; Angular *R*	8°-68°			UV–Vis-NIR, FTIR, AFM, FESEM	[77]
Mg_x_Ni/Pd/PEI/H_x_WO_3_/ITO		Spectral *ε*; IR imaging; CV		150s; 3h	Cycling failure; oxidation; cracking	TEM, EDS, XPS	[78]
BaF_2_/AZO NCs/ITO		Spectral *T*; Spectral *ε*		< 600 ms; > 10^4^ cycles		SEM	[79]
SiO_2_/TiN NPs/Ag	Air	Spectral *A*; Spectral *ε*; Angular *A*	-60° to 60°		Stable to 727 °C	SEM, TEM, DLS	[83]
HfN/Ag	NaCl solution	Spectral *R*		1–180 min; 10 d	Corrosion	TEM, XRD, XPS, FTIR	[84]
Ti_3_C_2_T_x_ MXene	Air	Spectral *A*; Spectral *ε*; IR imaging				IR imaging	[11]
MXene/CNT		Spectral *ε*; IR detection			Thermal shock resistant		[52]
Ti_3_C_2_T_x_		Spectral *ε*				FTIR, XRD	[99]
Graphene/BaO/Ag		Spectral *A*	Angle-tunable			COMSOL	[118]
Al_2_O_3_/rGO	Ar	Spectral *ε*		2 h	Anti-corrosion	SEM, TEM, AFM	[119]
La_0.16_(CaCuCoNiMg)_0.84_Mn_2_O_4_	Air	Spectral *ε*		prolonged exposure	Stable at 900 °C	XRD, EBSD, TEM, EDS	[126]
(Ga_0.2_Ni_0.2_Co_0.2_Mn_0.2_Zn_0.2_)_3_O_4_		Spectral *ε*		24 h; 12 h × 5 cycles	Stable after 1300 °C aging and 1800 °C cycling	XRD, HRTEM, EDS, XPS, Raman	[127]
(Al_0.25_Cr_0.25_Fe_0.25_Ga_0.25_)_2_O_3_	Air	Spectral *ε*		2 h	Stable at 800 °C	XRD, SEM, TEM, XPS	[50]
ZrC	Air	IR stealth; EMI shielding		80 s	Oxidation onset at 480 °C	XRD, XPS, SEM	[104]
SiC		Spectral *A*	75°			Simulation	[101]
Ge Grating/3C-SiC/Si Metasurface		Spectral *ε*	Wide-angle				[149]
Glass/Al_2_O_3_ radiative cooling glass	High humidity; water; UV; soiling; high temperature	Solar *R*; LWIR *ε*			Stable after water/UV/soiling and 1000 °C flame shock		[13]
Al_2_O_3_/glass LTCC		Solar *R*; LWIR *ε*		> 2000 h	UV-resistant; > 1000 °C tolerant		[19]
CaO/Ta_2_O_5_/Al_2_O_3_	Air	Spectral IR *T*		1–10 h		XRD, MAS NMR	[166]
MgF_2_/CaF_2_	Vacuum	Spectral IR *T*		2 h		XRD, SEM, FTIR	[173]
In[Ba_3_Cl_3_F_6_]	N_2_	Spectral IR *T*; adsorption–desorption				Single-crystal XRD, PXRD, FESEM, EDS, FTIR	[175]
ZnSe		Spectral IR *T*; angular *T*	0°-60°		Stable to 900 °C	SEM	[141]
Poly(S-r-BTT) Sulfur Polymers		MWIR/LWIR *T*; refractive index		1 h	Thermally stable to ~ 200 °C	FT-IR, XRD, DSC, DMA	[185]

## Radiative Control in Extreme Energy Systems

Application relevance emerges when material–intrinsic spectra are reconciled with aerodynamic heating, radiation exchange, mechanical load, and chemical exposure specific to each mission. The domains considered here represent nonoverlapping system functions that translate reflectance, emittance, and transmittance into concrete performance metrics. Each domain imposes a distinct hierarchy of constraints that ranges from erosion resistance and birefringence control in optical domes to oxide scale management on radiative skins, from spectral selectivity and durability in thermophotonic emitters to haze suppression and fouling tolerance in industrial process windows [1, 3]. The following case studies align material families and structure-informed design rules with these operational envelopes, highlighting where present solutions already enable credible deployment and where structural or chemical stability still governs adoption.

## Infrared Windows and Domes for Hypersonics and Re-entry

For hypersonic and re-entry infrared windows and domes, material selection is governed by coupled optical, thermal, chemical, and mechanical constraints [221]. Key requirements include high MIR transmittance in the 3–5 and 8–12 μm bands, wavefront stability under steep thermal gradients, oxidation/erosion resistance, and tolerance to thermal shock, vibration, and impact damage [222]. Candidate materials should therefore be evaluated not only by spectral transmission, but also by thermal conductivity, fracture toughness, hardness, thermal shock resistance, interfacial stability, and environment-coupled degradation [144]. Covalent semiconductors and diamond are attractive under high heat flux because strong bonding and high thermal transport suppress thermal lensing and thermal shock damage [148]. Oxide transparent ceramics, including sapphire, ALON, spinel, YAG, and yttria, are more suitable for oxidizing flows because of their hardness, stiffness, and corrosion resistance, although phonon absorption generally limits their use to the 3–5 μm region [165]. Lower-phonon-energy materials such as ZnS, ZnSe, CaF_2_, and BaF_2_ can extend transmission toward 8–12 μm, but require controlled humidity, thermal-gradient, and stress conditions to avoid hydration, sublimation, creep, or haze [183]. The abrupt transmittance loss near service limits should be treated as threshold degradation, because phase transitions, second-phase precipitation, volatilization-induced roughening, and rapid defect accumulation can sharply increase absorption and scattering once a critical temperature or stress state is reached [223].

**Fig. 19:**
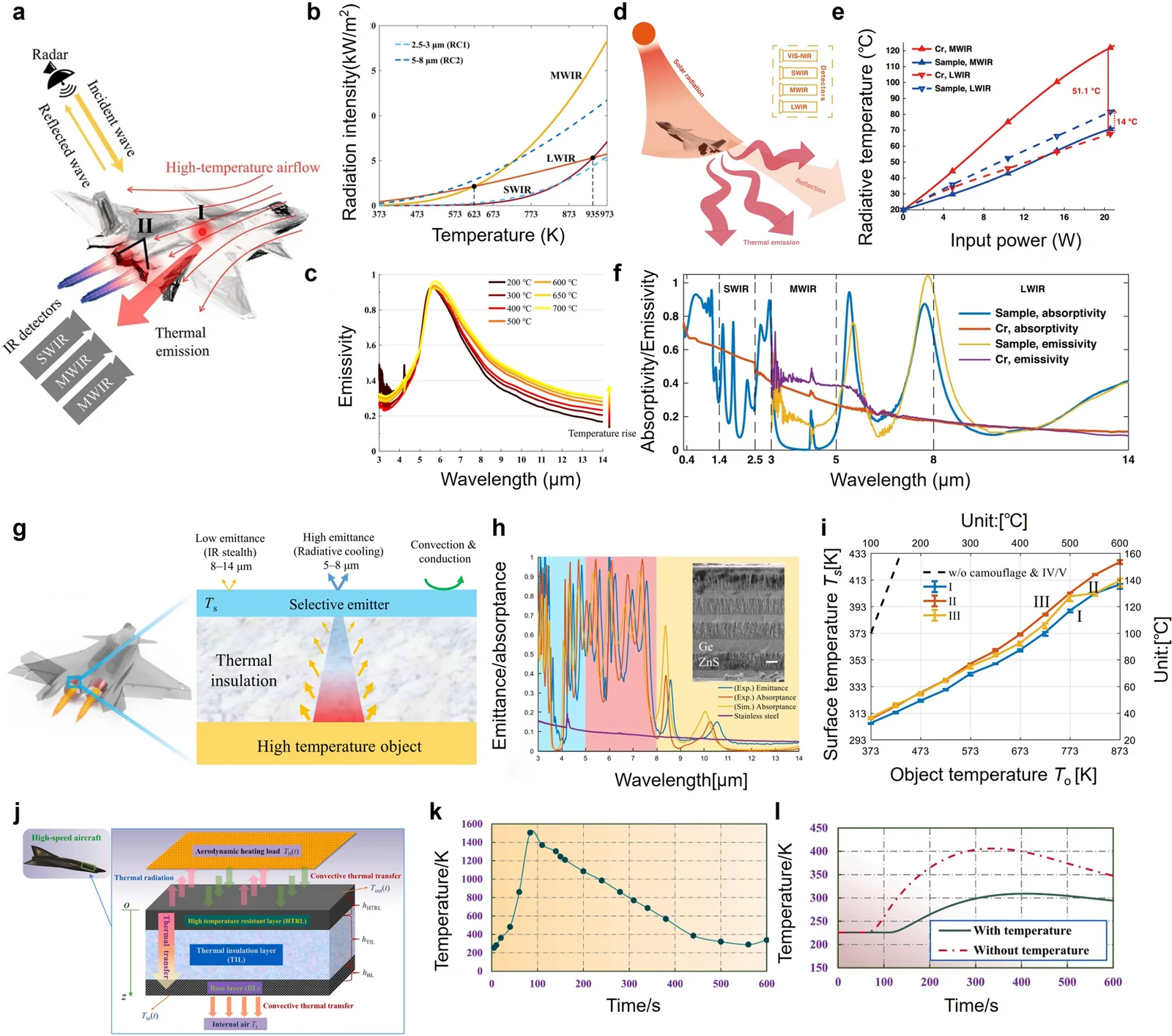
Infrared windows and domes for hypersonics and re-entry. **a** Schematic of high-temperature IR/microwave stealth integrated with thermal management. **b** Blackbody radiation intensity versus temperature in atmospheric and radiative heat bands. **c** Emissivity spectra of IR-selective emitter. Reproduced with permission [1]. Copyright 2025, Springer Nature. **d** Typical detection bands and primary signal sources from visible to LWIR. **e** Radiative temperature comparison of different surfaces. **f** Measured absorptivity and emissivity of sample and Cr reference. Reproduced with permission [224]. Copyright 2023, Springer Nature. **g** Multilayer emitter-insulator structure. **h** Spectral response of multilayer system. **i** Surface temperature under heating conditions. Reproduced with permission [225]. Copyright 2020, Springer Nature. **j** Schematic illustration of MTI structure. **k** Aerodynamic heating environment along flight trajectory. **l** Temperature responses of BL inner surface with and without thermal effects. Reproduced with permission [226]. Copyright 2024, Elsevier

Motivated by these requirements, several studies have explored materials and structures that combine MIR transmission or radiation control with high-temperature stability. Li et al. proposed an integrated high-temperature IR/microwave stealth and thermal management concept for multi-band detection scenarios (Fig. 19a) [1]. The defined RC1 and RC2 bands clarify how radiative cooling windows can be separated from atmospheric detection windows (Fig. 19b). Emissivity spectra from 3–14 μm at 200–700 °C confirm stable MIR radiation under steady thermal loading (Fig. 19c). However, this stability was mainly verified under steady-state heating, without direct evaluation of cracking, delamination, or thermal cycling reliability. Li et al. further demonstrated whole-infrared-band and visible-band camouflage compatible with radiative heat dissipation (Fig. 19d) [224]. MWIR radiative-temperature measurements showed a 51.1 °C reduction relative to a Cr reference under 20 W input power (Fig. 19e). Broadband absorptivity and emissivity spectra confirmed multi-band spectral regulation from the visible to MIR region (Fig. 19f). These results remain dominated by optical and thermal metrics, while microstructural evolution and interfacial stability under coupled loading are still insufficiently quantified.

To further optimize high-temperature IR performance, Li et al. developed a scheme combining a thermal insulator with a wavelength-selective emitter by depositing alternating Ge and ZnS multilayer films on a thin silica substrate (Fig. 19g), providing spectral selectivity in non-atmospheric windows (Fig. 19h) [225]. Under peak object temperatures of 873 K, the surface temperature of the selectively cooled sample was 16.9 K lower than that of the reference, confirming the effectiveness of radiative cooling in reducing thermal signature (Fig. 19i) [225]. Although effective thermal regulation is achieved, thermomechanical mismatch and long-term multilayer stability under repeated heating remain unresolved. Complementing these strategies, Yang et al. proposed a mid-infrared thermal insulation (MTI) architecture for hypersonic environments (Fig. 19j), demonstrating that the inner surface temperature remains stable (~ 320 K peak) under transient aerodynamic heating for ~ 100 s (Fig. 19k), highlighting the combination of thermal management and structural design (Fig. 19l) [226]. Overall, high-temperature IR windows and domes are shifting from single-material selection toward integrated designs combining spectral tuning, thermal insulation, interfacial engineering, and structural optimization. Future qualification should report transmittance retention together with atmosphere, exposure time, thermal cycling conditions, surface roughness/scale evolution, and post-test structural integrity, so that optical failure can be linked to chemical and mechanical degradation.

## Aerospace Thermal Protection and Radiative Surfaces

Aerospace thermal protection and radiative surfaces use MIR control to manage extreme heat loads while preserving structural margins [227]. Two application modes should be distinguished: sustained low-emittance/high-reflectance surfaces reduce radiative coupling with hot boundary layers, shock layers, or solar irradiation, whereas high-emittance surfaces act as radiators or selective emitters that require long-term spectral stability during heat rejection. Refractory metals and transition metal nitrides are suitable for low-emittance functions in inert or protected atmospheres [201], while conductive oxides and selected silicides are more relevant when oxygen activity cannot be avoided. Carbide and oxide ceramics provide broadband or phonon-assisted emission at elevated temperature [228], while polar lattices with Reststrahlen features enable narrower emission bands matched to mission requirements [229, 230]. Practical deployment further depends on oxidation resistance, ablation tolerance, thermal cycling stability, and resistance to erosion, vibration, and surface roughening [37, 50]. Therefore, microstructural density, adherent scales, clean interfaces [231], and mechanically compatible coatings are essential for maintaining emissivity targets and attachment reliability under flight-relevant loads [232].

**Fig. 20:**
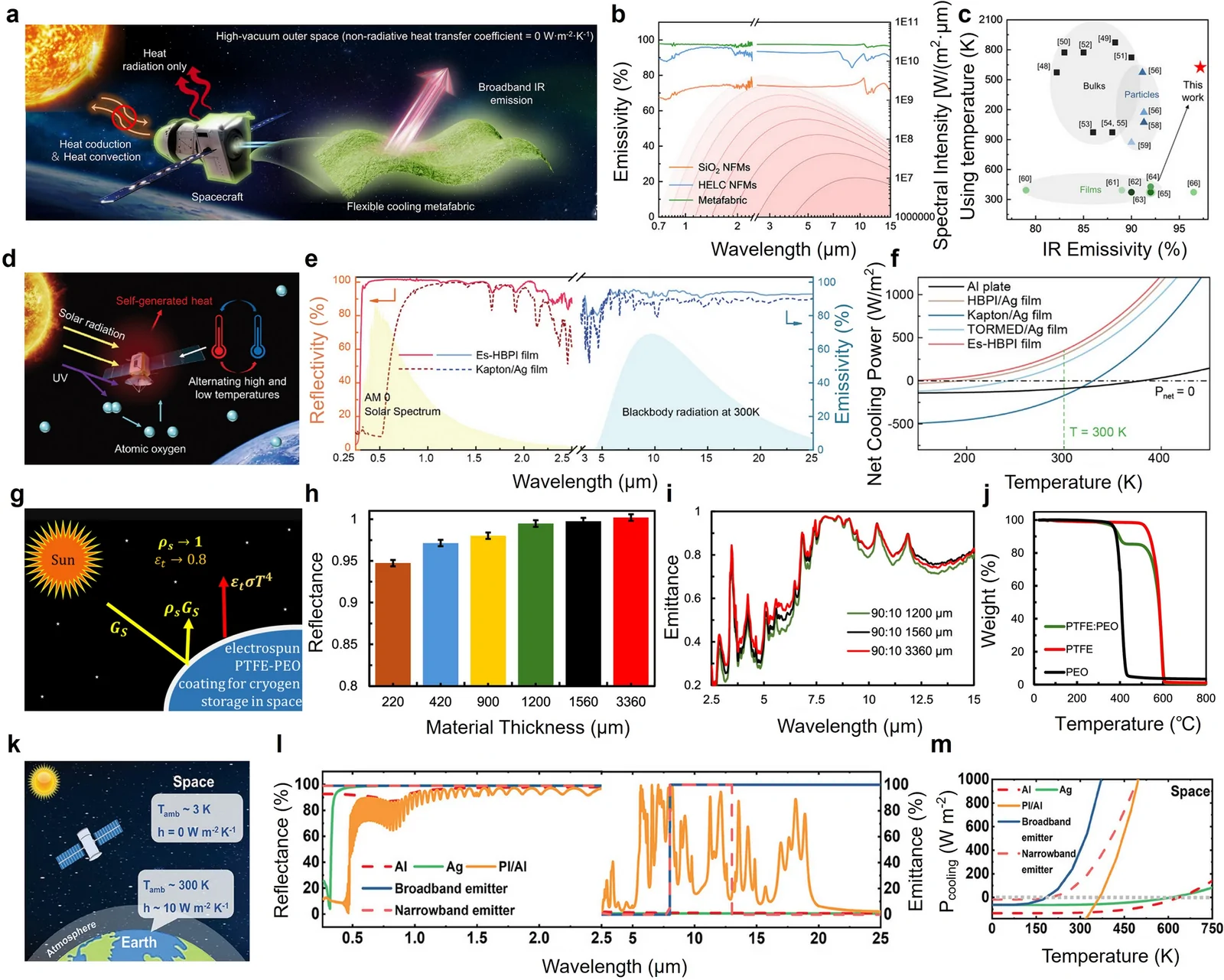
Aerospace thermal protection and radiative surfaces. **a** Spacecraft thermal management concept. **b** Spectral emissivity of SiO_2_ NFMs, HELC NFMs, and metafabric. **c** Comparative IR emissivity versus temperature for bulk, particle, and film samples. Reproduced with permission [2]. Copyright 2025, John Wiley and Sons. **d** Schematic of spacecraft thermal environment under extreme space conditions. **e** Reflectivity and emissivity of cooling films. **f** Net radiative cooling power of the studied films. Reproduced with permission [234]. Copyright 2024, John Wiley and Sons. **g** Radiative heat exchange of polymer nanofibers. **h** Effect of material thickness on average solar reflectance. **i** Thermal emittance of nanofiber assemblies. **j** TGA curves of PEO powder, PTFE particles, and electrospun PTFE:PEO nanofibers. Reproduced with permission [231]. Copyright 2024, American Chemical Society. **k** Schematic of environmental conditions on Earth and in space. **l** Spectral reflectance and emissivity of radiative materials. **m** Predicted cooling performance under different environments. Reproduced with permission [235]. Copyright 2024, John Wiley and Sons

Guided by these principles, several radiative surfaces and nanofibrous assemblies have been developed for aerospace thermal regulation [233]. Si et al. designed a metafabric for spacecraft thermal management under high-vacuum and extreme thermal conditions (Fig. 20a) [2]. Its MIR emissivity exceeds that of conventional SiO_2_ and HELC nanofibrous materials (Fig. 20b). Comparative thermal evaluation confirms stronger temperature regulation in both film and particulate forms (Fig. 20c). The hierarchical fiber architecture may buffer stress, but systematic thermomechanical failure and long-term environmental degradation remain insufficiently evaluated. Building on nanofibrous approaches, Fan et al. fabricated a radiative cooling film (RCF) based on electrospun polyimide nanofibers, optimized at molecular and microscale levels for extreme space conditions (Fig. 20d) [234]. Spectral measurements show enhanced solar reflectivity and thermal emissivity relative to a Kapton/Ag reference (Fig. 20e). The calculated net cooling power at 300 K demonstrates effective passive thermal management under space-relevant irradiation (Fig. 20f). Its reported resistance to UV exposure, atomic oxygen, and temperature cycling strengthens the link to outer-space radiative cooling, but longer coupled thermal–mechanical aging tests are still needed.

Further extending these strategies, Narayan et al. explored PTFE polymer nanofibers for passive spacecraft temperature control (Fig. 20g) [231]. The study investigated the influence of material thickness and composition on solar reflectance (Fig. 20h), showing that PTFE:PEO ratios of 90:10, 85:15, and 80:20 systematically modulate optical properties (Fig. 20i). Thermal emittance measurements at 300 K indicate that thicker nanofiber assemblies maintain high emissivity, enabling effective radiative heat rejection in orbit (Fig. 20j). UV/atomic-oxygen exposure, SEM observation, and tensile testing provide useful indirect mechanical evidence, although high-temperature thermomechanical coupling is not fully addressed. Wan et al. developed a universal silica aerogel radiative cooler from regenerated quartz fiber membranes (Fig. 20k) [235]. Spectral data show ultralow solar absorptance and near-ideal MIR emittance across key radiative bands (Fig. 20l). Theoretical calculations confirm superior cooling performance under both space and terrestrial boundary conditions (Fig. 20m). Overall, aerospace radiative surfaces should be selected according to whether the mission requires persistent low emittance to suppress heat input or stable high emittance to reject stored heat. Future qualification should pair emissivity retention with thermal cycling, adhesion, cracking/delamination resistance, erosion tolerance, and coupled environmental aging.

## High-Temperature Thermophotovoltaics and Thermal Photonics

High-temperature thermophotovoltaics and thermal photonics convert stored or waste heat into electrical or radiative work by shaping MIR spectra at temperatures where surfaces, interfaces, and lattices evolve rapidly [236, 237]. Selective emitters are required when photovoltaic cells accept a narrow energy window, whereas broadband emitters or radiators are preferred for heat rejection, calibration, or thermal management [238, 239]. Spectral recycling further requires high-reflectance mirrors or filters that return nonconvertible photons to the hot source; refractory metals and transition metal nitrides are effective in controlled atmospheres, while conductive oxides are more suitable in oxidizing environments [57, 81, 240]. Long-term performance is governed not only by initial spectral selectivity but also by oxidation, volatile sub-oxide formation, roughness growth, interdiffusion, and stress relaxation, which shift emissive peaks, raise out-of-band loss, and reduce usable photon flux [3, 241]. Therefore, deployable TPV and thermal-photonic systems require dense microstructures, compatible interfaces, redox stable stoichiometry, and thermal-expansion matching, together with device-level control of gap distance, view factor, and low-loss optical pathways [242, 243].

**Fig. 21:**
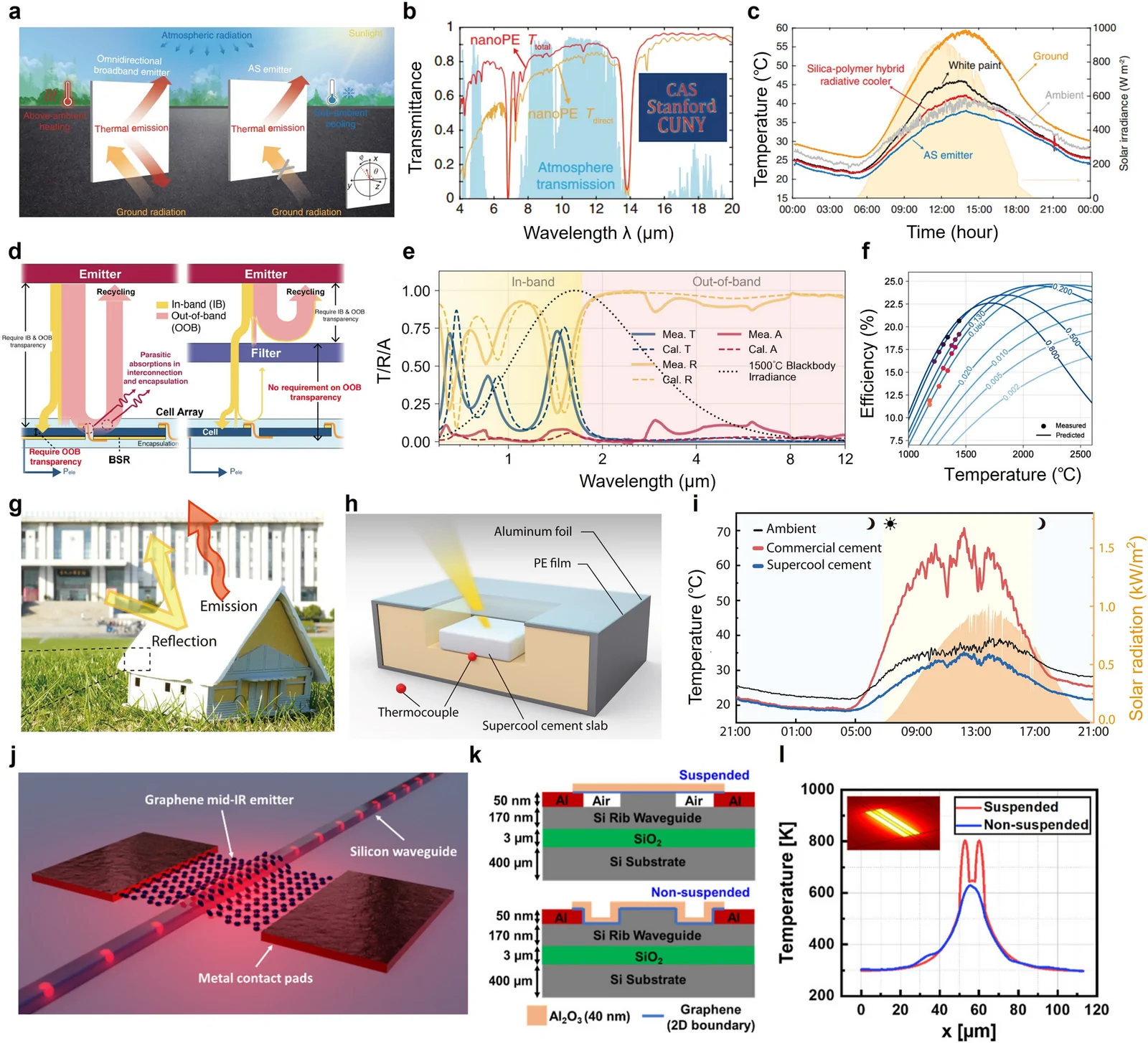
High-temperature thermophotovoltaics and thermal photonics. **a** Angular and spectral design of thermal emitter. **b** Infrared transmittance of nanoPE film. **c** Full-day solar irradiance, sample temperature, and deviation from ambient. Reproduced with permission [3]. Copyright 2024, AAAS. **d** Spectral management comparison in TPV systems using BSR versus filter. **e** Optical response of multilayer filter. **f** Conversion efficiency under different conditions. Reproduced with permission [244]. Copyright 2025, American Chemical Society. **g** Structure of radiative cooling cement. **h** Setup for in-field radiative cooling performance measurements. **i** Temperature response under solar irradiation. Reproduced with permission [245]. Copyright 2025, AAAS. **j** Graphene MIR emitters integrated with photonic waveguides for gas sensing. **k** Electro-thermal modeling of emitter structures. **l** Temperature distribution in graphene emitters. Reproduced with permission [246]. Copyright 2024, American Chemical Society

Several studies have translated these material-level principles into functional thermal-photonic devices. Li et al. analyzed vertically oriented broadband thermal emitters and showed that restricted sky access reduces daytime cooling efficiency (Fig. 21a) [3]. The nanoPE film preserves direct and total MIR transmittance, enabling angularly asymmetric emission (Fig. 21b). Full-day outdoor testing confirms temperature reduction under realistic solar irradiation (Fig. 21c). These results validate optical design under realistic radiative conditions, but prolonged polymer stability under combined thermal and environmental exposure remains unclear. Building on emitter design for thermophotovoltaic applications, Zhao et al. implemented back surface reflectors (BSRs) to recycle out-of-band radiation. Near-perfect mid-infrared reflectance was achieved up to 2.75 μm, with 96.7% measured reflectivity (Fig. 21d, e) [244]. Efficiency assessments under emitter temperatures of 1184–1441 °C and varying view factors confirmed significant improvements in conversion efficiency (Fig. 21f). However, the effect of prolonged high-temperature operation on multilayer integrity and interfacial stability remains insufficiently explored.

Complementary strategies focus on large-area and integrated photonic platforms for thermal management. She et al. demonstrated supercool cement that combines high solar reflectance with elevated MIR emission (Fig. 21g) [245]. Field testing under natural conditions (Fig. 21h) recorded peak temperature reductions of 5.4 °C relative to ambient and 26.0 °C relative to commercial cement under ~ 850 W m^−2^ solar irradiance (Fig. 21i), evidencing both high-temperature durability and effective radiative cooling. Compared with coating-based systems, the bulk-integrated cement matrix may better resist surface degradation and mechanical wear during outdoor service. Lemme et al. explored graphene-based mid-infrared emitters integrated with photonic waveguides (Fig. 21j) [246]. Electro-thermal simulations revealed that suspended graphene sheets reached 803 K under 185 mW power, whereas substrate-supported graphene reached 629 K (Fig. 21k), highlighting the role of support in thermal stability and emissive performance (Fig. 21l). Overall, TPV and thermal-photonic systems are progressing from material-level spectral tuning toward integrated control of emission, recycling, and thermal pathways. Future work should report spectral drift, interfacial stability, oxidation state, and cycling-induced degradation under extended high-temperature service.

## Harsh-Environment Industrial Infrared Optics and Process Monitoring

Harsh-environment industrial infrared optics and process monitoring require stable MIR transmission, calibrated radiance, and reliable imaging inside furnaces, reactors, and high-temperature manufacturing lines [247]. The main service constraints are thermal shock, corrosive vapors, particulate erosion, fouling, and repeated heating/cooling. Oxide transparent ceramics such as sapphire, ALON, spinel, YAG, and yttria remain preferred for 3–5 μm viewports because they combine oxidation resistance, hardness, and chemical durability [165, 248]. Silicon and CVD diamond are useful when high heat flux requires simultaneous optical transmission and heat spreading, while fluorides and Zn-based chalcogenides extend monitoring toward longer wavelengths but require dry purge streams and moderated thermal gradients to avoid hydration, sublimation, creep, or haze [173, 183]. Anti-reflection and anti-fouling surfaces must resist soot, alkali salts, sulfates, crystallization, and crazing; therefore, compatible dielectric stacks, replaceable sacrificial coupons, or subwavelength texturing are favored when they preserve a smooth optical surface. Beyond windows, graphitic and carbide emitters provide near-gray pyrometry standards, while conductive oxides and nitrides can serve as low-emittance shields against stray heating [37, 116]. Reliable deployment therefore depends on matching material chemistry, surface engineering, mounting design, purge flow, and thermal profile to the specific industrial environment.

**Fig. 22:**
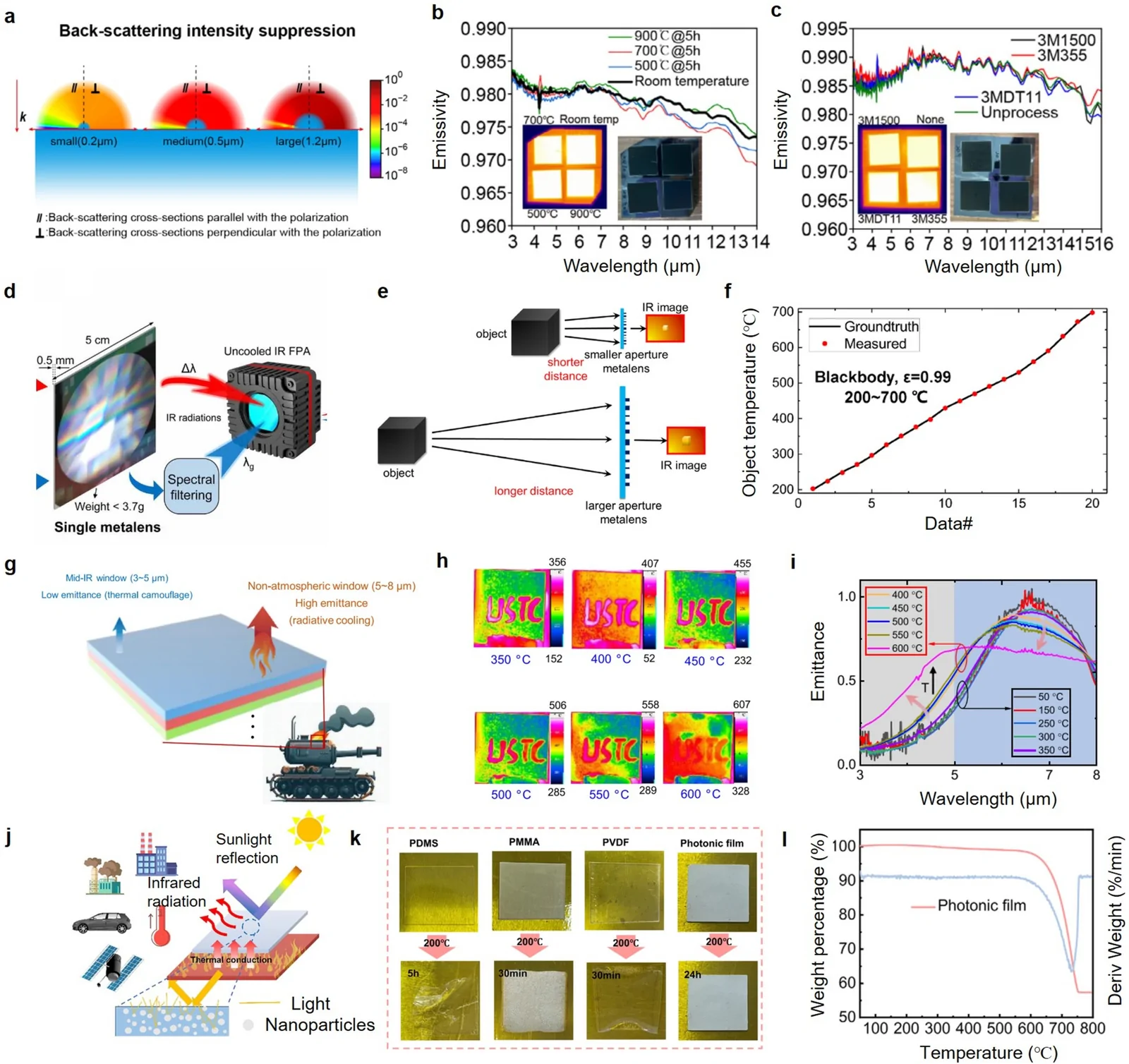
Harsh-environment industrial infrared optics and process monitoring. **a** Light-scattering behavior of nanostructured surfaces. **b** Thermal stability of emissive coatings. **c** Mechanical stability after surface treatment. Reproduced with permission [4]. Copyright 2025, John Wiley and Sons. **d** Metalens-based thermographic imaging system. **e** Long-distance infrared imaging configuration. **f** Temperature measurement comparison with reference. Reproduced with permission [14]. Copyright 2024, AAAS. **g** Multiband thermal camouflage and cooling design. **h** Infrared imaging at elevated temperature. **i** Measured emissivity of structure 2 under varying temperatures. Reproduced with permission [249]. Copyright 2024, De Gruyter. **j** Structure of high-temperature photonic film. **k** Thermal resistance of polymer-based films. **l** Thermal analysis of photonic materials. Reproduced with permission [238]. Copyright 2025, John Wiley and Sons

Building on these mechanistic insights, several groups have developed materials and devices capable of retaining mid-infrared performance under extreme industrial conditions. Sun et al. used ultrafast laser direct irradiation to form planar blackbody surfaces on doped silicon without a separate coating layer, reducing interfacial failure risk (Fig. 22a) [4]. Thermal exposure at 500–900 °C confirmed short-term emissivity retention after heating and cooling (Fig. 22b). Tape-stripping produced only minor emissivity variation, indicating short-term mechanical robustness of the textured surface (Fig. 22c). Importantly, the demonstrated 23 × 23 mm^2^ samples and 60 × 60 mm^2^ monolithic area indicate practical scalability, but lifetime validation should still include repeated thermal cycling, prolonged high-temperature aging, fouling/cleaning tests, and emissivity mapping after service. Yi et al. developed a lightweight, metalens-based thermographic camera (MTC) with a single 0.5-mm-thick, 3.7-g mass-producible metalens (Fig. 22d), which enhanced resolving power and enabled long-distance imaging (Fig. 22e) [14]. Validation against blackbody references from 200–700 °C confirmed measurement reliability (Fig. 22f). The monolithic nanopillar architecture provides structural continuity, although its durability under prolonged thermal loading and environmental exposure is not explicitly quantified. These works highlight how engineered surfaces and compact optical architectures preserve mid-infrared clarity, emissivity, and imaging fidelity under harsh thermal and mechanical stresses.

Extending mid-infrared functionality to ultrahigh-temperature applications, Ye et al. implemented a material informatics-based inverse design framework to achieve combined thermal camouflage (low emittance in 3–5 μm) and radiative cooling (high emittance in the 5–8 μm non-atmospheric window) (Fig. 22g) [249]. Thermal imaging at temperatures exceeding 350 °C (Fig. 22h) and corresponding emittance measurements across multiple temperature points validated both functional performance and design strategy (Fig. 22i). Despite effective spectral control, long-term multilayer stability under thermal mismatch and environmental exposure remains to be clarified. Hou et al. developed a high-temperature-resistant radiative cooling film by integrating 2D hexagonal boron nitride nanosheets into a melt-processable PFA polymer matrix, achieving optimized thermal transport and mechanical integrity (Fig. 22j) [238]. Heat-resistance tests confirmed superior thermal durability relative to conventional polymer films (Fig. 22k), and TGA/DSC analyses indicated decomposition above 600 °C and melting at 313 °C (Fig. 22l). Overall, harsh-environment MIR optics are moving from passive windows toward integrated surfaces that combine emissivity control, imaging, thermal regulation, and contamination resistance. For industrial deployment, future studies should verify scalable fabrication, repeated thermal cycling, high-temperature dwell stability, adhesion or abrasion resistance, fouling/cleaning durability, and post-test spectral/emissivity mapping.

## Summary, Challenges, and Perspectives

**Fig. 23:**
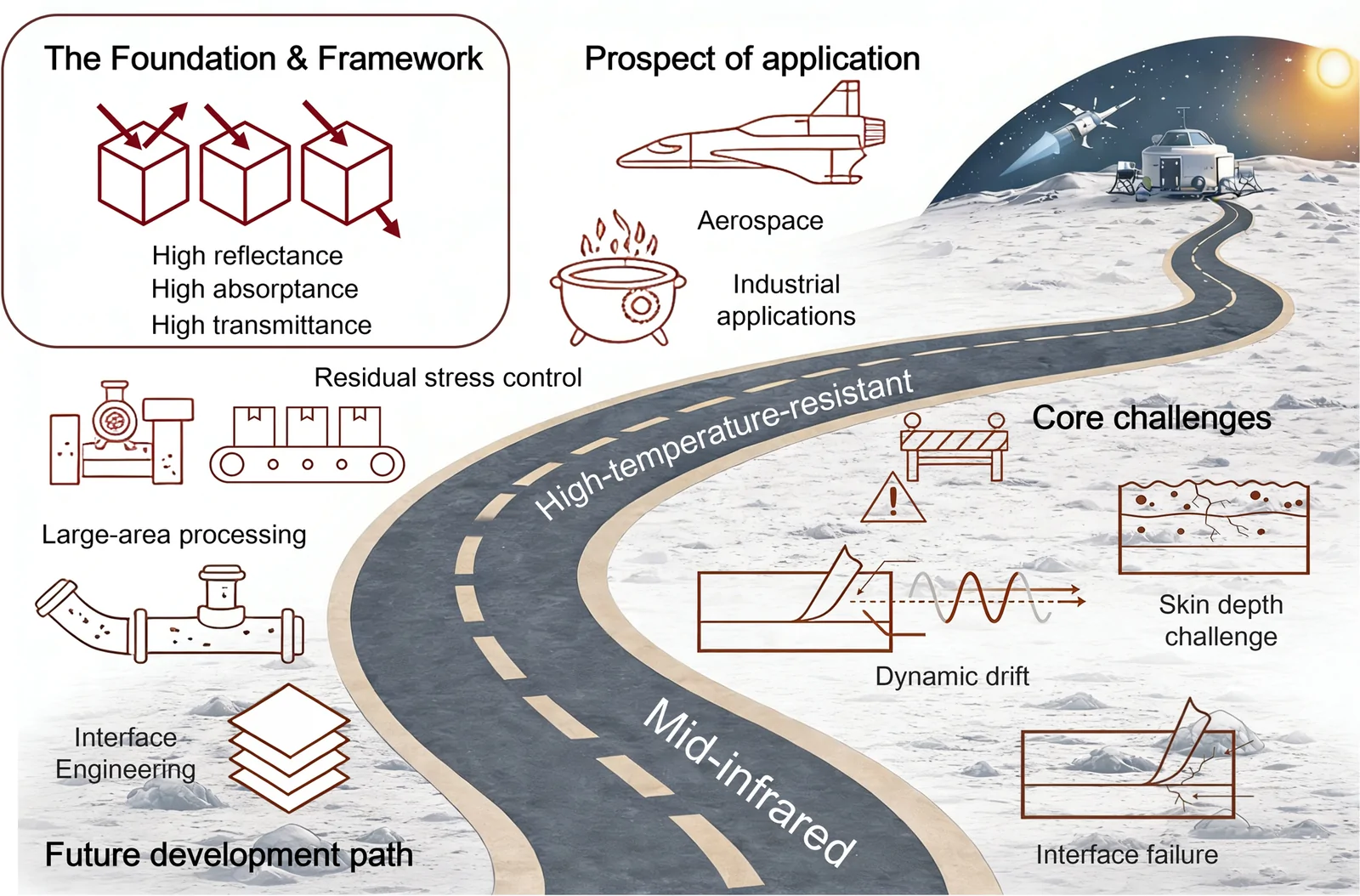
Challenges and pathways for high-temperature mid-infrared materials toward system-level deployment

This review consolidates a fragmented body of research into a structure-informed framework that connects intrinsic material chemistry and structure to durable mid-infrared function under coupled thermal, chemical, and mechanical loads. By resolving materials into three functional classes-high reflectance, high absorptance/emittance, and high transmittance-and interpreting their behavior through electronic/defect structure, crystallography/phase stability, microstructure/mesostructure, and surface/interface evolution, this review clarifies how targeted MIR spectra can be achieved and sustained in demanding environments. The synthesis of material classes, spectral metrics, and verified temperature limits enables more meaningful comparison across systems and shows why room temperature optimization alone cannot predict in-service performance. Collectively, these insights reposition high-temperature MIR capability from a static materials attribute to a system-level design lever, establishing a foundation for assessing challenges, opportunities, and future pathways **(**Fig. 23**)**.

- (1)A central challenge is that many high-temperature MIR properties are governed by the near-surface region, where oxide scale growth, roughness evolution, and defect segregation directly modify reflectance, emissivity, or transmittance. This issue mainly originates from surface/interface evolution and microstructural instability, especially when roughness, cracks, or oxide scales approach the MIR optical penetration depth. Future studies should co-measure optical spectra with oxide thickness, surface roughness, and near-surface chemistry under controlled atmospheres, with practical targets such as limiting emissivity or reflectance drift to Δ*ε* or Δ*R/R*_*0*_ ≈ 0.05–0.10 during relevant thermal exposure.
- (2)High-temperature MIR performance is also sensitive to carrier concentration, vacancy density, and phonon lifetime, which drift with temperature, atmosphere, and stress. This challenge is primarily linked to electronic/defect structure and phase stability, and it affects high-reflectance conductors, tunable emitters, and polar materials by shifting plasma edges, broadening phonon resonances, or increasing optical loss. Future work should establish temperature-atmosphere-defect maps using operando optical, electrical, and structural probes, with testable criteria such as <10% spectral drift after >100h exposure at 800-1000 °C, or above 1000 °C for refractory candidates in representative atmospheres.
- (3)In practical components, optical degradation often initiates at coatings, diffusion barriers, bonding layers, or protective scales rather than in the bulk. This problem reflects surface/interface instability coupled with microstructural evolution, and it can degrade all three optical functions by increasing scattering, absorption, or interfacial reflection loss. Interface design should therefore be integrated into materials selection, with targets such as maintaining interface-induced emissivity increase below Δ*ε* ≈ 0.05 during thermal cycling while tracking roughness, diffusion, and phase evolution.
- (4)Many high-temperature MIR materials are still validated using small specimens or simplified thermal tests, whereas large-area apertures, thick windows, conformal radiative skins, and multilayer stacks introduce porosity, texture, residual stress, and coating nonuniformity. This scale-up challenge is mainly associated with microstructure/mesostructure, but it also amplifies defect heterogeneity and interface failure. Future qualification should combine thermal cycling, reactive atmospheres, erosion or mechanical loading, and operando optical diagnostics, with the goal of keeping component-scale reflectance, emissivity, or transmittance within ~10% of laboratory-scale values under comparable service conditions.

Beyond the challenges discussed above, several practical directions can make future research more actionable.

- (1)In situ and operando characterization should be selected according to optical function. High-reflectance materials require temperature-dependent ellipsometry and infrared reflectance to track carrier damping and plasma-edge shifts; high-emittance systems require emissivity mapping coupled with in situ X-ray diffraction or Raman spectroscopy to connect phonon/phase evolution with spectral drift; high-transmittance materials require in situ transmission and haze/scattering analysis to distinguish absorption from pore-, crack-, or surface-induced scattering.
- (2)Spectral drift should be quantitatively correlated with measurable structural descriptors. For reflectors, emissivity or reflectance drift should be linked to oxide scale thickness, roughness amplitude, carrier mobility, and vacancy concentration; for emitters, peak position or bandwidth changes should be related to phase evolution, phonon broadening, and interfacial reactions; for windows, transmittance loss should be separated into absorption, scattering, and surface reflection contributions. These datasets would support stability maps that define oxidation-limited temperatures, allowable defect windows, and failure thresholds for key candidate materials.
- (3)Qualification protocols should better reproduce coupled service environments. Instead of relying only on room temperature spectra or end-point tests after heating, future studies should combine heat flux, controlled oxygen or nitrogen partial pressure, thermal cycling, dwell aging, erosion, vibration, and post-test optical/structural mapping. Such protocols would help distinguish intrinsic optical degradation from surface roughening, defect drift, interface failure, or scale-up-induced nonuniformity.

In summary, future progress should move from discovering materials with attractive room temperature spectra toward engineering radiative behavior that remains stable during operation. By focusing on quantitative stability windows, operando characterization, robust interfaces, scalable processing, and coupled service testing, high-temperature MIR materials can progress from promising demonstrations to dependable components for aerospace, energy, and harsh-environment technologies.
